# Sustainable Design for the Direct Fabrication and Highly Versatile Functionalization of Nanocelluloses

**DOI:** 10.1002/gch2.201700045

**Published:** 2017-09-13

**Authors:** Samson Afewerki, Rana Alimohammadzadeh, Sinke H. Osong, Cheuk‐Wai Tai, Per Engstrand, Armando Córdova

**Affiliations:** ^1^ Department of Natural Sciences Mid Sweden University Holmgatan 10 851 70 Sundsvall Sweden; ^2^ Department of Chemical Engineering Mid Sweden University Holmgatan 10 851 70 Sundsvall Sweden; ^3^ Department of Materials and Environmental Chemistry The Arrhenius Laboratory Stockholm University 106 91 Stockholm Sweden

**Keywords:** click chemistry, heterogeneous catalysis, nanocellulose, organocatalysis

## Abstract

This study describes a novel sustainable concept for the scalable direct fabrication and functionalization of nanocellulose from wood pulp with reduced energy consumption. A central concept is the use of metal‐free small organic molecules as mediators and catalysts for the production and subsequent versatile surface engineering of the cellulosic nanomaterials via organocatalysis and click chemistry. Here, “organoclick” chemistry enables the selective functionalization of nanocelluloses with different organic molecules as well as the binding of palladium ions or nanoparticles. The nanocellulosic material is also shown to function as a sustainable support for heterogeneous catalysis in modern organic synthesis (e.g., Suzuki cross‐coupling transformations in water). The reported strategy not only addresses obstacles and challenges for the future utilization of nanocellulose (e.g., low moisture resistance, the need for green chemistry, and energy‐intensive production) but also enables new applications for nanocellulosic materials in different areas.

Cellulose is the most abundant and most versatile biopolymer on earth.^1^, ^2^ This renewable, nontoxic, and hydrophilic polysaccharide has excellent material properties, which have made it very useful to society. Cellulose is also a perfect material for the development of a bio‐based and circular economy. The partial depolymerization of cellulosic materials can lead to the fabrication of nanocellulose.^3^, ^4^, ^5^, ^6^, ^7^, ^8^, ^9^, ^10^, ^11^ There is significant research activity on future applications of this cellulose‐derived nanomaterial by scientists in both industry and academia. For example, nanocelluloses could be used in functional coatings, barrier coatings, strength additives, films, emulsions, foams, optical devices, adhesives, composites, biomedical engineering materials, cement, packaging, fillers, nonwoven materials, textiles, and separation membranes.^3^, ^4^, ^5^, ^6^, ^7^, ^8^, ^9^, ^10^, ^11^ Two types of nanocellulose^12^ can be derived from the cellulose in wood: nanofibrillated cellulose (NFC) and nanocrystalline cellulose (NCC). NFC is spaghetti‐like in structure, long and flexible, composed of fibers less than 100 nm in width and several micrometer in length, containing both crystalline and amorphous regions. NCC consists mostly of rod‐like crystals (no amorphous regions) with diameters in the range of 10–20 nm and lengths of a few hundred nanometers. Therefore, different chemical methods have been developed for their production. NCC is made by hydrolysis, generally using inorganic acids (e.g., sulfuric acid and phosphoric acids), and produces highly crystalline and rigid nanoparticles.^13^, ^14^, ^15^, ^16^ Since the amorphous region of the cellulose fibril is degraded in the process, the yield is low (30–50% yield, <7 MJ kg^−1^ energy consumption of the nano conversion). NFC fabrication from wood‐derived pulp fibers was first achieved by mechanical agitation (high energy consumption, generally >30 000 kWht^−1^, 700–1400 MJ kg^–1^)^7^, ^13^, ^14^ and chemical–mechanical methods, e.g., the carboxymethylation route (high energy, generally >30 000 kWht^−1^, 700–1400 MJ kg^−1^).^7^, ^15^ The 2,2,6,6‐tetramethylpiperidine‐1‐oxyl (TEMPO)‐sodium hypochlorite (NaClO) oxidation‐homonigenization production method for NFC requires significantly less energy (<7 MJ kg^−1^).^7^, ^16^ However, the initial primary alcohol oxidation pretreatment requires a radical mediator and a chlorine‐based oxidant. Enzymatic hydrolysis–homogenization treatment routes for NFC production also require little energy, but biocides must be added to maintain enzyme stability.^17^ Life cycle analyses have shown that all these fabrication processes exhibit prominent environmental advantages over other nanomaterials such as carbon nanotubes.^18^, ^19^ Among the fabrication processes, the enzymatic pretreatment route and mechanical agitation exhibit the lowest environmental impact. However, enzyme stability and the energy required for mechanical agitation are important cost factors. With respect to chemical treatment methods, the (TEMPO)–NaClO oxidation–homogenization route has a lower impact than the carboxymethylation route. However, the use of an active radical initiator and a nonselective chlorine‐based oxidant is a disadvantage in large‐scale production.

Cellulose and nanocellulose materials are highly moisture‐sensitive, and their surfaces are difficult to modify under environmentally benign conditions. In fact, the direct modification of heterogeneous cellulose using nonactivated reagents is challenging, and it is therefore commonly dissolved prior to modification.^20^ In 2005, Hafrén and Córdova reported the direct functionalization and hydrophobization of heterogeneous lignocelluloses using organocatalysis.^21^, ^22^ These approaches have also recently been applied to the direct surface functionalization of nanocellulose.^23^, ^24^ The introduction of a specific functional group on the cellulose allows the further modification of its surface using “click chemistry”,^25^, ^26^, ^27^, ^28^, ^29^ a concept introduced by Sharpless and co‐workers.^25^ Here, we describe the direct fabrication and catalytic functionalization of nanocellulose under environmentally benign conditions.

Inspired by the above challenges in nanocellulose research, we designed a new concept for their selective production and fabrication. Here, the organic acid‐mediated hydrolysis of wood pulp is combined with modular surface engineering using organic catalysis and “click” chemistry (**Figure** **1**).^30^ The “organoclick” chemistry functionalization in our strategy should allow the selective surface modification of nanocellulose for different applications. Like the enzyme pretreatment production route for NFCs, it has the advantage of being mild, but it should also be more robust, and the organic acid could be readily recycled. Currently, however, organic acids such as formic acid are generally employed for the complete or significant depolymerization of wood biopolymers to monomeric units (e.g., monosaccharide and aryl units).^31^, ^32^ Thus, the literature suggests that a potential NFC fabrication process might be hard to control and result in low yields. This possibility is further supported by the recent production of NCC in 42–70% yield from bleached chemical hardwood pulp (silver birch (*Betula pendula*)) using formic acid in combination with HCl.^33^ Despite this information, we began to investigate NFC fabrication using an organic acid process.^30^, ^34^ For recent oxalic acid pretreatment of kraft bleached Eucaluptus pulp in combination with disk milling for fabrication of NFC, see ref. 34.

**Figure 1:**
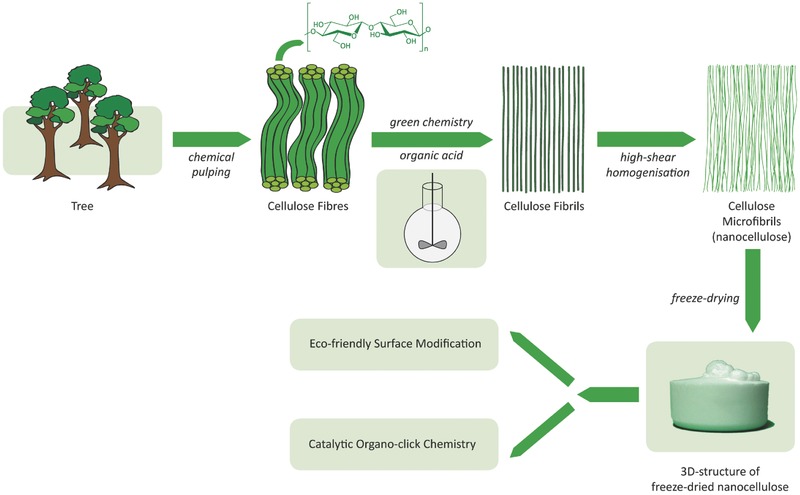
Eco‐friendly design: The direct acid‐mediated route to NFC and the “organoclick” surface functionalization of nanocellulose.

Bleached sulfite softwood dissolving pulp (10 g dry mass of pulp (70% Norway spruce (*Picea abeis*) and 30% Scots pine (*Pinus sylvestris*))) were pretreated with formic acid at various temperatures to give white milky dispersions in clear, colorless spent liquors. This step was followed by homogenization. To our delight, we discovered that we could produce NFC in high yields (97–99%, dry weight) at relatively low temperatures (70–90 °C). Transmission electron microscopy (TEM) studies of the fine white powder revealed separated fibers with nanoscale diameters (<20 nm, **Figure** **2**). The formic acid remaining from this fabrication of NFC was also successfully recovered and reused for another cycle of NFC production. Compared with the NFC derived from the TEMPO–NaClO oxidation–homogenization of bleached sulfite softwood dissolving pulp, the NFC derived from formic acid does not have charged groups on its surface and can therefore aggregate upon standing. Therefore, the formic acid‐derived NFC was converted to a foam in high yields (97–99%) after its fabrication. The NFC generated from the TEMPO–NaClO oxidation–homogenization route was also converted to foam. We also successfully produced NFC by an initial catalytic aerobic oxidation (TEMPO/O_2_)‐formic acid treatment. This simple metal‐free aerobic oxidation/organic acid protocol use oxygen instead of NaClO as the terminal oxidant and can also introduce charges onto the NFC surface. Fourier transform infrared (FT‐IR) spectroscopic analysis of the formic acid‐fabricated NFC revealed that the surface had also been esterified by formic acid (degree of substitution (ds) <0.1). However, the NFC was not hydrophobic. The ester bonds were readily removed by basic treatment with NaOH solution (1 m) prior to homogenization and freeze‐drying, as shown by FT‐IR analysis of the corresponding NFC foam. The formic acid method exhibited the same performance when scaled up (25 g dry mass of pulp) to give fine NFC, which was also converted to a foam. This reaction was performed in parallel (2 × 25 g) and gave the same results. Satisfied with the development of this mild and scalable NFC production route, we embarked on the organocatalytic surface modification of the NFC foam. The silane bond is robust and more stable in basic conditions than the ester bond, and there are many commercial silanes that could be attached by this silylation method. A screen of different acids for their ability to catalyze the silylation of 3‐phenylpropionalcohol with thiapropylsilane (TPSi) 1a or allylsilane 1c revealed that (*S*)‐tartaric acid and malic acid were excellent catalysts for this transformation. Thus, a variety of different silanes (1a–d) were investigated as substrates for the direct catalytic silylation of NFC using (*S*)‐tartaric acid as the catalyst (**Scheme** **1**). Elemental analysis revealed that the surfaces of all NFC samples had been modified (ds < 0.6). The direct catalytic attachment of hydrophobic silanes to the surface of the nanocellulosic materials made the materials water repellent (Figure 2). Metal‐free catalyzed silylation was also performed on the NFC derived using the TEMPO–NaClO homogenization method. This carboxy‐functionalized NFC also became hydrophobic after treatment. Performing the same procedure with filter paper as a substrate enabled us to measure the contact angle for the attachment of silanes, which had a range of 96°–130° (1a–d). The direct attachment of organic amines such as aminopropyl (AmP) silane 1e is also notable, as the corresponding amino‐functionalized NFC could be used as a sustainable support for catalysts (e.g., metals and enzymes) and as a binder of metals.^8^, ^35^ Here, the AmP‐NFC foam material efficiently bound Pd(II) salts to form AmP‐NFC‐Pd(II)‐complexes. In addition, the subsequent reduction of Pd(II) with NaBH_4_ gave the corresponding AmP‐NFC‐Pd(0) foam containing Pd nanoparticles (Figure 2). This AmP‐NFC‐Pd(0) served as a heterogeneous catalyst and efficiently catalyzed Suzuki cross‐couplings in water (See Supporting Information).

**Figure 2:**
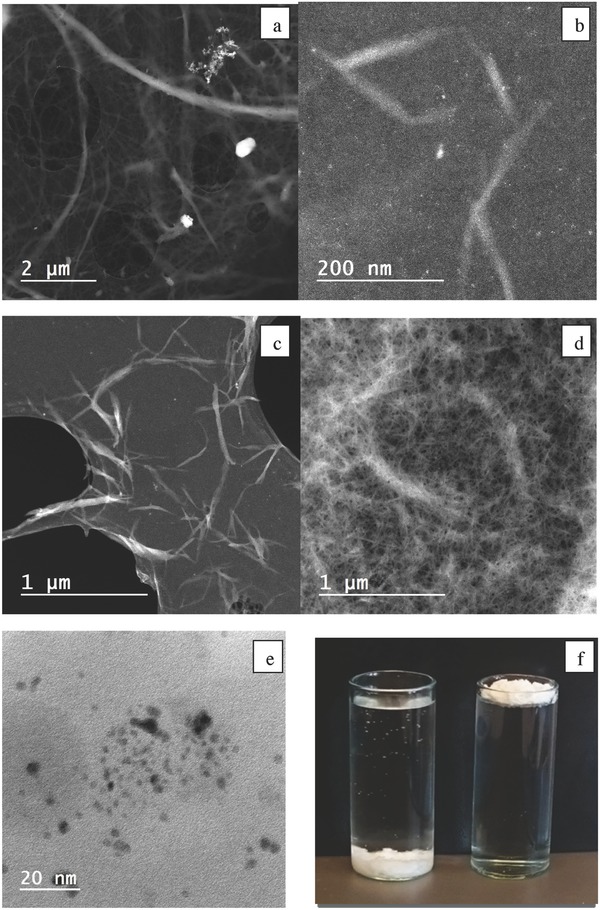
TEM images of different samples: a) Sulfite softwood dissolving pulp. b] Formic acid‐fabricated NFC. c) Formic acid‐fabricated NFC. d) NFC derived from the TEMPO–NaClO oxidation–homogenization route. e) Scanning Transmission electron micrograph bright field (STEM‐BF) Image of AmP‐NFC‐Pd(0). f) The left vial contains NFC and H_2_O. The right vial contains hydrophobic C16‐NFC and H_2_O.

**Scheme 1:**
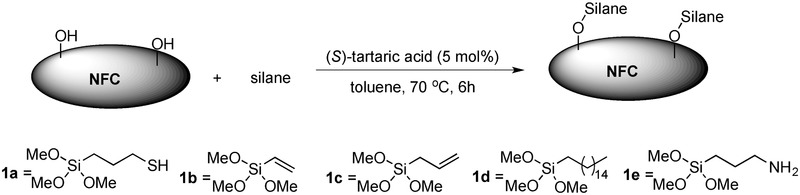
Direct organocatalytic surface modification of heterogeneous NFC with Silanes 1.

Having established a versatile and direct method for organocatalytic nanocellulose modification, we next began to investigate the “organoclick” strategy for the functionalization of NFC using first (*S*)‐tartaric acid and then UV light. Thus, the direct catalytic modification of NFC foam with TPSi 1a or allylsilane 1c (Figure 1, **Scheme** **2**) was followed by thiol‐ene click reactions on the corresponding TPSi‐1a‐NCF and 1c‐NCF, respectively, using a UV lamp and a metal‐free initiator (Scheme 2). Thiol‐end‐group‐functionalized polyesters were chosen as the initial substrates for reaction with the allyl‐1c‐NFC, as the polyester groups could be readily detected by FT‐IR (Scheme 2a). After the click reaction and Soxhlet extraction, FT‐IR clearly showed that the polyesters poly(ε‐caprolactone) (PCL) and poly(δ‐valerolactone) (PVL) were attached to the NFC. We also attached *n*‐octane‐1‐thiol by the click reaction. This reaction occurs much faster than the analogous reactions using the polyester substrates. The “click” reaction was also monitored by ^1^H NMR spectroscopy of the reaction between *n*‐octane‐1‐thiol or 6‐mercaptohexan‐1‐ol‐initiated PCL with trimethoxyallylsilane 1c.

**Scheme 2:**
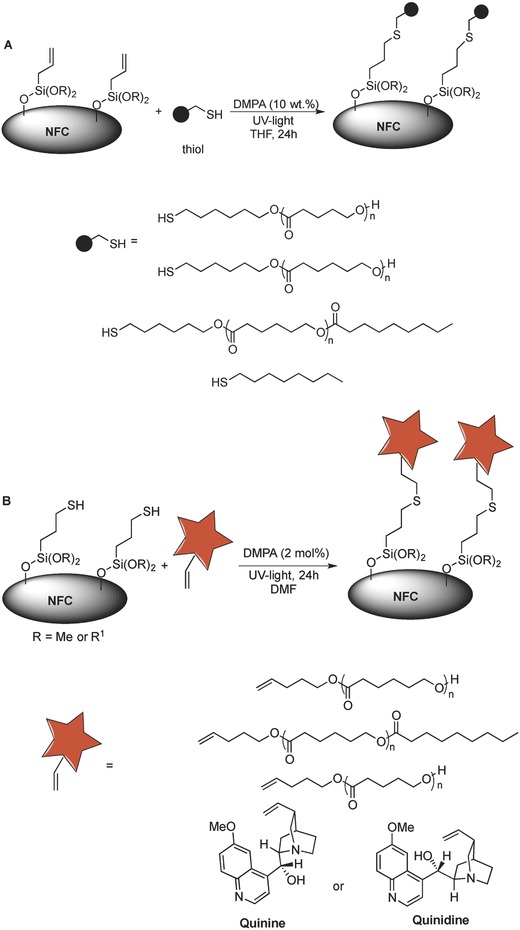
a) Click reaction between Allyl‐1c‐NFC and 6‐Mercaptohexan‐1‐ol‐Initiated Polyesters or *n*‐Octane‐1‐Thiol. b) Click Reaction between TPSi‐1a‐NFC and olefin‐initiated polyesters or cinchona alkaloids.

The thiol‐ene click reactions between TPSi‐1a‐NFC and 4‐pentene‐1‐ol‐initiated polyesters (PCL and PVL) produced by chemoselective enzyme‐ or (*S*)‐tartaric acid‐catalyzed polymerizations, respectively, were also investigated (Scheme 2b). The reactions proceeded smoothly, generating PCL‐ and PVL‐functionalized NFCs, respectively.

An important factor in the engineering of the cellulose surface is the attachment of fluorescent and UV‐active groups with applications in sensors and biology (e.g., cell uptake and viability). However, the introduction of a functional group onto the NFC surface is not eco‐friendly, and hazardous solvents are employed (pyridine).^21^ Another important potential application for NFC is as a sustainable supporting material for organic molecules that serve as ligands or catalysts, which would make the cellulose surface catalytically active and allow applications including diagnostics and heterogeneous catalysis. Therefore, we decided to investigate the attachment of the pharmaceutical agents quinidine and quinine to the NFC surface (Scheme 2b). These natural products isolated from the bark of the cinchona tree exhibit important biological activities (e.g., antimalaria, antiarrhythmic heart, lupus, and arthritis). They are also used as fluorescent markers, organocatalysts, and organic chiral ligands for metal catalysts.^36^, ^37^ They also mediate several important asymmetric reactions (e.g., conjugate additions, oxidations, kinetic resolutions, and Sharpless asymmetric dihydroxylation) used in selective organic synthesis.^37^ Consequently, the formic acid fabrication of NFC was followed by its direct “organoclick” functionalization with cinchona alkaloids. Here, (*S*)‐tartaric acid catalyzed the direct silylation of NFC with 1a to give TPSi‐1a‐NFC, followed by the thiol‐ene “click” reaction with quinine under UV light and a metal‐free photocatalyst. The same procedure was also performed with quinine as the cinchona alkaloid. After basic washing and extensive Soxhlet extraction, we investigated the fluorescent activity of the 1a‐NFCs treated with cinchona alkaloids. Both the quinidine‐TPSi‐1a‐NFC and quinine‐TPSi‐1a‐NFC were fluorescent, whereas the TPSi‐1a‐NFC and NFC were not (**Figure** **3**). In the control experiments, we also mixed NFC and TPSi‐1a‐NFC with quinidine for 24 h in dimethylformamide (DMF) and found that these NFC materials did not exhibit fluorescence activity after basic washing and Soxhlet extraction with acetone. The above “organoclick” surface engineering strategy was also successful on TEMPO–NaClO produced NFC, NCC and filter paper, and several thiol‐ or olefin‐functionalized molecules were directly attached to the allyl‐ or TPSi‐modified cellulosic materials, respectively (see SI). Thus, this approach is a broadly versatile strategy for the direct surface engineering of nanocellulose and cellulosic materials.

**Figure 3:**
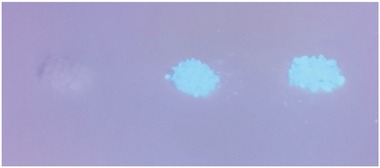
UV lamp (long waveLength): Left: 3‐thiapropylsilane‐(TPSi)‐1a‐modified NFC (blank). Middle: Quinidine‐TPSi‐1a‐NFC. Right: Quinine‐TPSi‐1a‐NFC.

The NFC foams can also be modified with different silanes 1 using (*S*)‐tartaric acid as the catalyst. For example, reacting the surface of the NFC foam with equal amounts of TPSi 1a and allyl silane 1c (1a:1c‐1:1) resulted in the corresponding 1a–c‐NFC foam material with a 1:1 ratio between 1a:1c, as measured by elemental analysis. The ratio and concentration of the silanes 1 were found to be important for the organocatalytic modifications. We next performed a selective thiol‐ene click reaction between quinidine and 1a–c‐NFC to produce the fluorescent quinidine‐1a–c‐NFC.

In conclusion, we have developed an eco‐friendly strategy for the scalable fabrication and surface engineering of NFC. The nanocellulose production method requires low energy input and provides high yield, and the formic acid mediator can be recycled. It also avoids the use of radical initiators and chlorine‐based oxidants, which produce organochlorines and persistent pollutants including dioxins in large‐scale industrial production. The incorporation of direct “organoclick” engineering of the NFC using metal‐free catalysis and UV light broadens the concept. For example, the NFC was made hydrophobic, and different types of functional silane groups (e.g., alkyl, thia, amino, and olefinic groups) were introduced to its surface using organic acids as catalysts. Here, the AmP‐functionalized nanocellulose (AmP‐NFC) could bind metal salts. It was also converted to an AmP‐NFC‐Pd(0)‐nanoparticle catalyst for C—C bond‐forming reactions. Performing click reactions on the thiol and olefin‐functionalized NFC under UV light allowed further surface engineering. Biodegradable hydrophobic molecules (e.g., alkanes and polyesters produced by “green” catalysis) and valuable cinchona alkaloids (e.g., antimalaria, fluorescent, metal‐free catalyst, and ligands for asymmetric synthesis) were smoothly attached to the nanocellulose. Thus, the reported nanocellulose fabrication strategy (Figure 1) overcomes obstacles and challenges in the future development of nanocelluloses, such as achieving eco‐friendly production and functionalization as well as addressing water and moisture sensitivity. It also facilitates new applications of nanocellulose in different areas (e.g., pharmaceuticals, electrodes, sensors, and as heterogeneous catalyst) with fully sustainable materials and chemistry.

## Conflict of Interest

The authors declare no conflict of interest.

## Supporting information

Supplementary
