# Supplementary material for: Sustainable Design for the Direct Fabrication and Highly Versatile Functionalization of Nanocelluloses
Source: Glob Chall. 2017 Sep 13;1(7):1700045. doi: 10.1002/gch2.201700045 (PMC6607377; doi:10.1002/gch2.201700045)
Supplement: Supplementary file 1 — Supplementary [file GCH2-1-1700045-s001.pdf]

Copyright WILEY-VCH Verlag GmbH & Co. KGaA, 69469 Weinheim, Germany, 2017.

Global Challenges Open Access

Supporting Information

for *Global Challenges*, DOI: 10.1002/gch2.201700045

Sustainable Design for the Direct Fabrication and Highly Versatile Functionalization of Nanocelluloses

*Samson Afewerki, Rana Alimohammadzadeh, Sinke H. Osong, Cheuk-Wai Tai, Per Engstrand, and Armando Córdova**

Table of Contents for the Supplementary Information:

General 7

Condition screening for the formic acid treatment of sulphite softwood dissolving pulp 9

Figure S1. *High speed homogenizing* 10

Figure S2. *High speed homogenizing zoomed picture* 10

Table S1. Reaction conditions for the formic acid treatment of sulphite softwood dissolving pulp for 24h. 10

Typical procedure for the large scale formic acid treatment of sulphite softwood dissolving pulp 11

Typical procedure for the recycling of formic acid 11

General procedure for the NFC processing using TEMPO-NaClO-oxidation-homogenization method 12

Figure S3. *NFC-gel (2%w/v) after TEMPO-NaClO-oxidation-homogenization processing* 12

Freeze-drying of the NFC-gel 12

Figure S4. Foam material from freeze-dried NFC: The left foam material is NFC produced using the TEMPO method. The right foam material is NFC derived from the formic acid method. 13

Typical procedure for the tandem catalytic aerobic oxidation/formic acid treatment of sulphite softwood dissolving pulp 13

General procedure for basic treatment of all formic acid derived NFC or foam materials used in the functionalization experiments: Removal of formic acid ester groups 13

Typical procedure 1 for the silylation of NFC 14

Table S2. Elemental analysis of the silylated NFC derived by formic acid method or TEMPO-NaClO-oxidation method. 14

Procedure for silylation of NFC with 1a and 1c and it subsequent UV-treatment under "click" conditions 15

Table S3a. Elemental analysis of "click" reacted **1a-1c** modified NFC. 16

Table S4. Data of the NFC foam materials. 16

Typical procedure 2 for the silylation of NFC. 17

Typical procedure 2 for the silylation of NFC with 1a and 1c. 18

Table S3b. Elemental analysis of NFC modified with silane **1a** and **1c** using procedure 2. 18

Procedure for Lipase-catalyzed synthesis of 6-mercaptohexan-1-ol-initiated poly(ε-caprolactone) or 4- pentene-1-ol-initiated poly(ε-caprolactone). 18

Typical procedure for the (*S*)-tartaric acid-catalyzed synthesis of alcohol-initiated poly(δ-valerolactone) (PVL) 19

Typical procedure for lipase-catalyzed alcohol-end group esterification of 6-mercaptohexan-1-ol-initiated-PCL and 4-Pentene-1-ol-initiated-PCL with nonanoic acid: 20

Procedure for the thiol-ene 'click' reaction between 1c-NFC and 6-Mercaptohexan-1-ol initiated PVL, 6-Mercaptohexan-1-ol initiated PCL or Nonanoic acid-terminated-6-mercaptohexan-1-ol-initiated-PCL 21

Table S5. Elemental analysis of thiol-ene click reaction between allyl-**1c**-NFC and 6-mercaptohexan-1-ol initiated PCL. 21

Procedure for the thiol-ene 'click' reaction between allyl-1c-NFC and 1-octanethiol 21

Procedure for the thiol-ene 'click' reaction between TPSi-1a-NFC and 4-Pentene-1-ol-initiated PCL, 4- Pentene-1-ol-initiated PVL or nonanoic acid 4- pentene-1-ol-initiated PCL 21

Procedure for the thiol-ene 'click' reaction between TPSi-1a-NFC or 1a-1c-NFC and Quinidine or Quinine 22

Table S6. Elemental analysis of Quinidine-TPSi-**1a**-NFC. 22

Figure S5. Modified NFC under a UV-lamp (Short wave length): Left: TPSi-**1a**-NFC (blank). Middle: Quinidine-TPSi-NFC. Right: Quinidine-TPSi-NFC. 22

Figure S6. Modified NFC under a UV-lamp (Short wave length): Left: NFC. Middle: **1a**-**1c**-NFC. Right: Quinididine-**1a**-**1c**-NFC. 23

Procedure for the synthesis of NFC-AmP-Pd(0) heterogeneous catalyst 23

General procedure for the acid catalyzed screening of allyltrimethoxysilylation 1c to 3-phenylpropionalcohol: 26

Graf S1. *Acid screening for the silylation with silane* ***1c*** *and 3-phenylpropionalcohol* 26

General procedure for the silylation of 3-phenylpropionalcohol with 1a: . 27

Graf S2. *Acid screening for the silylation with silane* ***1c*** *and 3-phenylpropionalcohol* 27

Procedure for the thiol-ene 'click' reaction between (3 mercaptopropyl)trimethoxysilane 1a and Quinidine[7]: 28

General procedure for the acid screening of silylated filter paper: 28

Table S8. Acid screening for the silylation of filter paper with silane **1a**. 29

General procedure for the silylation on filter paper: 29

Table S9. Silylation of filter paper 30

General procedure for the thiol-ene 'click' reaction between modified filter paper and alkene or thiol: 30

General procedure for the thiol-ene 'click' reaction between modified filter paper and polymer: 31

Table S10. Click reaction of filter paper modified with silane **1a** 31

Table S11. Click reaction of filter paper modified with silane **1b** 32

Table S12. Click reaction of filter paper modified with silane **1c** 33

Table S13. Elemental analysis of silylated filter paper 33

Figure S8. *A water droplet on a hydrophobized filter paper with C-16 hexadecene* 34

Procedure for the thiol-ene 'click' reaction between TPSi-1a-modified-filter paper and Quinidine or Quinine 34

Figure S9. *UV-activity of modified filter paper (short wave):* 34

Figure S10. *UV-activity of modified filter paper (long wave):* 34

References 35

Figure S11. *N_2 sorption isotherms of formic acid fabricated NFC foam material* 36

Figure S12. *Pore size distribution of formic acid fabricated NFC foam material using the adsorption isotherms with density functional theory* 36

Figure 13. *N_2 sorption isotherms of formic acid fabricated NFC foam material performed at 80 ^oC* 37

Figure S14. *Pore size distribution of formic acid fabricated NFC foam material performed at 80 ^oC using the adsorption isotherms with density functional theory* 37

Figure S15. *N_2 sorption isotherms of formic acid fabricated NFC foam material* 38

Figure S16. *Pore size distribution of formic acid fabricated NFC foam material using the adsorption isotherms with density functional theory* 38

Figure S17. *N_2 sorption isotherms of hydrophobized formic acid fabricated NFC foam material with C-16 silane **1d*** 39

Figure S18. *Pore size distribution of hydrophobized formic acid fabricated NFC foam material with C-16* ***1d*** *using the adsorption isotherms with density functional theory* **39**

Figure S19. *N_2 sorption isotherms of formic acid fabricated NFC silylated without acid with silane* ***1a*** **40**

Figure S20. *Pore size distribution of formic acid fabricated NFC silylated without acid with silane* ***1a*** **40**

Figure S21. *N_2 sorption isotherms of AmP-NFC from formic acid fabricated NFC* **41**

Figure S22. *Pore size distribution of AmP-NFC from formic acid fabricated NFC* **41**

Figure S23. *N_2 sorption isotherms of AmP-NFC from NFC derived from TEMPO-NaClO oxidation-homogenization route* **42**

Figure S24. *Pore size distribution of NFC-AmP from NFC derived from TEMPO-NaClO oxidation-homogenization route* **42**

Figure S25. *N_2 sorption isotherms of NFC with equal amounts of* ***1a*** *and* ***1c*** *and it subsequent UV-treatment under "click" conditions. Formic acid fabricated NFC was used* **43**

Figure S26. *Pore size distribution of NFC with equal amounts of* ***1a*** *and* ***1c*** *and it subsequent UV-treatment under "click" conditions using the adsorption isotherms with density functional theory. Formic acid fabricated NFC was used* **43**

Figure S27. *N_2 sorption isotherms of NFC with equal amounts of* ***1a*** *and* ***1c*** *and it subsequent UV-treatment under "click" conditions. NFC derived from TEMPO-NaClO oxidation-homogenization route was used* **44**

Figure S28. *Pore size distribution of NFC with equal amounts of* ***1a*** *and* ***1c*** *and it subsequent UV-treatment under "click" conditions using the adsorption isotherms with density functional theory. NFC derived from TEMPO-NaClO oxidation-homogenization route was used* **44**

Figure S29. *N_2 sorption isotherms of AmP-NFC-Pd(0). Formic acid fabricated NFC was used* **45**

Figure S30. *Pore size distribution of NFC-AmP-Pd(0) using the adsorption isotherms with density functional theory. Formic acid fabricated NFC was used* .. **45**

Figure S31. *High-angle annular dark-field scanning transmission electron micrograph (HAADF-STEM) image of sulphite-softwood-dissolving pulp* **46**

Figure S32. *High-angle annular dark-field scanning transmission electron micrograph (HAADF-STEM) image of formic acid fabricated NFC foam material* **46**

Figure S33. *High-angle annular dark-field scanning transmission electron micrograph (HAADF-STEM) image of formic acid fabricated NFC with 50% formic acid* **47**

Figure S34. *High-angle annular dark-field scanning transmission electron micrograph (HAADF-STEM) image of formic acid fabricated NFC at 80 °C* **47**

Figure S35. *High-angle annular dark-field scanning transmission electron micrograph (HAADF-STEM) image of formic acid fabricated with 180 minutes homogenization time* **48**

Figure S36. *TEM image of formic acid fabricated NFC with 180 minutes homogenization time and 48h reaction time* **48**

Figure S37. *High-angle annular dark-field scanning transmission electron micrograph (HAADF-STEM) image of NFC derived from TEMPO-NaClO oxidation-homogenization route* **49**

Figure S38. *High-angle annular dark-field scanning transmission electron micrograph (HAADF-STEM) image of NFC with equal amount of* ***1a*** *and* ***1c*** *and it subsequent UV-treatment under "click" conditons. NFC derived from TEMPO-NaClO oxidation-homogenization route was used* **49**

Figure S39. *STEM image of NFC with equal amount of* ***1a*** *and* ***1c*** *and it subsequent UV-treatment under "click" conditions. Formic acid fabricated NFC was used* **50**

Figure S40. *TEM image of hydrophobized formic acid fabricated NFC with C-16 silane* ***1d*** **50**

Figure S41. *High-angle annular dark-field scanning transmission electron micrograph (HAADF-STEM) image of hydrophobized NFC with C-16 silane* ***1d****. NFC derived from TEMPO-NaClO oxidation-homogenization route was used* **51**

Figure S42. *TEM image of formic acid and ammonium formiate fabricated NFC* **51**

Figure S43. *High-angle annular dark-field scanning transmission electron micrograph (HAADF-STEM) image of tandem catalytic aerobic oxidation/formic acid fabricated NFC* **52**

Figure S44. *Transmission electron micrograph bright field (STEM-BF) image* AmP-NFC-Pd(0). **52**

Figure S45. *FT-IR of formic acid fabricated NFC* **53**

Figure S46. *FT-IR of NFC derived from TEMPO-NaClO oxidation-homogenization route* **54**

Figure S47. *FT-IR of NFC derived from TEMPO-NaClO oxidation-homogenization route silylated with* ***1a*** **55**

Figure S48. *FT-IR of NFC derived from TEMPO-NaClO oxidation-homogenization route silylated with* ***1c*** **56**

Figure S49. *FT-IR of formic acid fabricated NFC silylated with* ***1e*** **57**

Figure S50. *FT-IR of NFC derived from TEMPO-NaClO oxidation-homogenization route silylated with* ***1e*** **58**

Figure S51. *FT-IR of NFC with equal amounts of **1a** and **1c** and it subsequent UV-treatment under "click" conditions. Formic acid fabricated NFC was used* **59**

Figure S52. *FT-IR of NFC with equal amounts of **1a** and **1c** and it subsequent UV-treatment under "click" conditions. NFC derived from TEMPO-NaClO oxidation-homogenization route was used* **60**

Figure S53. *FT-IR of click reaction between NFC silylated with **1c** and 6-mercaptohexan-1-ol-initated PCL:* **61**

Figure S54. *FT-IR of click reaction between NFC silylated with **1c** and 6-mercaptohexan-1-ol-initated PVL:* **62**

Figure S55. *FT-IR of click reaction between NFC silylated with **1c** and nonanoic acid-terminated-4-Pentene-1-ol-initiated PCL:* **63**

Figure S56. *FT-IR of click reaction between NFC silylated with **1a** and 4-Pentene-1-ol-initiated-PCL:* **64**

Figure S57. *FT-IR of click reaction between NFC silylated with **1a** and nonanoic acid-terminated-4-Pentene-1-ol-initiated PCL:* **65**

Figure S58. *FT-IR of click reaction between NFC silylated with **1a** and 4-Pentene-1-ol-initiated-PVL:* **66**

Figure S59. *FT-IR of click reaction between filter paper silylated with **1a** and 4-Pentene-1-ol-initiated-PVL:* **67**

Figure S60. *FT-IR of click reaction between NFC silylated with **1a** and Quinidine:* **68**

Figure S61. *FT-IR of click reaction between NFC silylated with **1a** and Quinine:* . **69**

Figure S62. *FT-IR of click reaction between NFC silylated with **1a** and Quinidine:* **70**

Figure S63. *FT-IR of click reaction between filter paper silylated with **1a** and Quinine:* **71**

Figure S64. *FT-IR of click reaction between filter paper silylated with **1a** and Quinidine:* **72**

Figure S65. *FT-IR of click reaction between NFC silylated with **1c** and 1-octanethiol:* **73**

Figure S66. *^{1}H NMR spectrum of 6-Mercaptohexan-1-ol-initated-PCL* **74**

Figure S67. *^{13}C NMR spectrum of 6-Mercaptohexan-1-ol-initated-PCL* **75**

Figure S68. *^{1}H NMR spectrum of 6-Mercaptohexan-1-ol-initated-PVL* **76**

Figure S69. *^{13}C NMR spectrum of 6-Mercaptohexan-1-ol-initated-PVL* **77**

Figure S70. *^{1}H NMR spectrum of 4-Pentene-1-ol-initated-PCL* **78**

Figure S71. ^{13}C NMR spectrum of 4-Pentene-1-ol-initated-PCL 79
Figure S72. ^{1}H NMR spectrum of 4-Pentene-1-ol-initated-PVL 80
Figure S73. ^{13}C NMR spectrum of 4-Pentene-1-ol-initated-PVL 81
Figure S74. ^{1}H NMR spectrum of allyldimethoxy(3-phenylpropoxy)silane 82
Figure S75. ^{13}C NMR spectrum of allyldimethoxy(3-phenylpropoxy)silane 83
Figure S76. High resolution mass of allyldimethoxy(3-phenylpropoxy)silane 84
Figure S77. ^{1}H NMR spectrum of 3-(dimethoxy(3-phenylpropoxy)-propane-1-thiol 85
Figure S78. ^{13}C NMR spectrum of 3-(dimethoxy(3-phenylpropoxy)-propane-1-thiol 86
Figure S79. High resolution mass of 3-(dimethoxy(3-phenylpropoxy)-propane-1-thiol 87
Figure S80. MWD curve (by SEC in 05% LiCl/DMAC) of the formic acid route fabricated NFC. 88
Figure S81. Pd nanoperticle size distrubution. 89

General

^{1}H NMR spectra were recorded on a Bruker Avance (500 MHz) spectrometer. Chemical shifts are reported in ppm from tetramethylsilane with the solvent resonance resulting from incomplete deuterium incorporation as the internal standard ($CDCl_3$: δ 7.26 ppm). Data are reported as follows: chemical shift, multiplicity (s = singlet, d = doublet, q = quartet, br = broad, m = multiplet), and coupling constants (Hz), integration. ^{13}C NMR spectra were recorded on a Bruker Avance (125.8 MHz or 100 MHz) spectrometer with complete proton decoupling, Chemical shifts are reported in ppm from tetramethylsilane with the solvent resonance as the internal standard ($CDCl_3$: δ 77.16 ppm). GC analysis was performed on GC Varian 3300x, chiral, BETA-DEX 325 column (30 m, 0.25mmx0.25μM), with He as carrier gas. High-resolution mass spectrometry was performed on Agilent 6520 Accurate-Mass Q-TOF LC/MS (positive mode). Elemental analyses were carried out by Medac LTD Analytical and chemical consultancy services (United Kingdom) by ICP-OES. Chemicals and solvents were either purchased *puriss p. A.* from commercial suppliers or were purified by standard techniques. Commercial reagents were used as purchased

without any further purification. Infrared spectrum was recorded with a Varian 610 IR microscope coupled to a Varian 670-IR spectrometer. Gas sorption measurements were carried out on a Micrometrics ASAP2020 analyzer an recored at 77K. Specific surface area was calculated by the Brunauer-Emmet-Teller (BET) equation, adsorption average pore width was calculated by the Barret-Joyner-Halenda (BJH) equation and the total pore volume was calculated by the Barret-Joyner-Halenda (BJH) equation. All TEM experiments were carried out on a 200 kV JEOL JEM-2100F field-emission electron microscope equipped with an ultra-high-resolution pole piece. A Gatan ultra-high tilt tomography holder was used. TEM samples were prepared by crushing, and the tomography data was acquired between -60° and +60° with 1° increments. Each image per tilt angle was recorded with a Gatan Ultrascan 1000 camera. The data acquisition was assisted by a commercial tomography packed, TEMography (version 2.15.07) developed by JEOL System Technology Co. Ltd. The contact angles were recorded on DAT 1100-fibro-system ab-SWEPEN. Measurement method is T 558 PM-95.The pulp used in this work was produced from a commercial sulphite softwood dissolving pulp (Domsjö Fabriker AB, Örnsköldsvik, Sweden) with a very low content of hemicellulose (<3%) and lignin (<1%) with 15% consistency (mass of dry pulp/(mass of dry pulp + mass of rest of the suspension). The wood material used was made of 70% fresh Norway spruce (Picea abies) and 30% Scot pine (Pinus sylvestris), with a mixture of 90% round wood and 10% saw mill chips. The average Mw of the pulp was 549987 and the PDI was 10.72. The fibrillation equipment used was IKA T 25 ULTRA TURRAX High Speed Homogenizer for the homogenization trials. The machine was set at 15,000 rpm and mechanical treatment time for each trial was 90 min per 1L of approximately 2% w/v pretreated pulp sample. MUNKTELL FILTER PAPER was used as filter paper cellulose source. Pieces cut from the filter paper was dried overnight at 40 °C. The tartaric acid was dried in a desiccator over phosphorus pentoxide. The freeze-dryer was a LAB360AB, SCANLAF Coolsafe 110-4 Pro, 2010 from Lynge Denmark. The SEC system consists of a DGU-20A3 degasser (Shimadzu), a LC-20AD liquid chromatography (Shimadzu), a Rheodyne 7725i fixed loop (100 µl) and a RID-10A refractive index detector (Shimadzu). The injection volume is 100 µl. The separation system consists of a mixed-A 20 µm guard column (7.5.50 mm, Polymer Laboratories) and 4 mixed-A 20 µm columns (7.5.300 mm, Polymer Laboratories). The flow rate is set constantly at 0.5 ml/min. The columns are thermostated at 80°C and the eluent is

0.5% LiCl/DMAc. The pullulan standards (Fluka) with nominal molar weights of 1.3 MDa, 805 KDa, 366 KDa, 210 KDa, 113 KDa, 48.8 KDa, 21.7 KDa, 10 KDa, 6 KDa, 1.32 KDa, and 342 Da are used for composing the calibration curve (Mw vs. retention time). The linear coefficient of determination (r^2) is 0.996. LC Solution software (Shimadzu) is applied to control the system as well as record data. The nanocellulose sample were by dissolved in LiCl/DMAC (final conc. 0.5 wt%) and analyzed by SEC.[1]

Condition screening for the formic acid treatment of sulphite softwood dissolving pulp

To a round bottom flask (500 mL) was poured concentrated formic acid (200 mL). Next, sulphite softwood dissolving pulp (66.7 g, 15% consistency, 10 g dry mass of pulp) was added and the mixture was heated to the temperature stated in Table S1. After stirring the suspension with a mechanical IKA RW 20 digital stirrer (2,400 rpm) for 24h, the reaction temperature was decreased to room temperature and next ultra sonicated in a bath for 1h using a BANDELIN SONOREX DIGITEC. Next, the reaction mixture was diluted with water (800 mL) and neutralized to pH = 7 by NaOH (aq. 1 M). Afterwards the suspension was centrifuged with EBA 21 Hettich ZENTRIFUGEN with 6,000 rpm for 10 min and the supernatant was decanted away. The heterogeneous precipitate was further washed by water and subsequent centrifugation; this procedure was repeated three times. The material was further homogenized with IKA T 25 ULTRA TURRAX High Speed Homogenizer (15,000) rpm for 90 min. The material was then put in a beaker and placed in liquid nitrogen at temperature less than -198 ^{o}C before freeze-drying. Liquid nitrogen was preferred as opposed the normal freezer, as it is a fast-freezing method and this method helps in preserving the pore size distribution of the 3D-structure. The yields of dry NFC foam (Table S1) were generally >97% based on the initial amount of sulphite softwood dissolving pulp (Table S1).

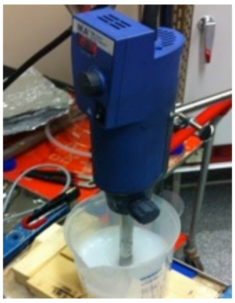

Figure S1. *High speed homogenizing*

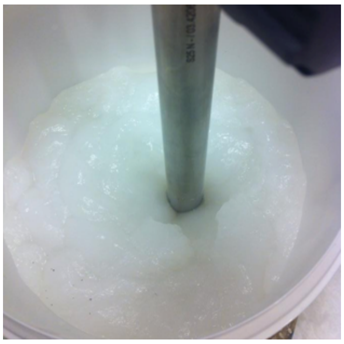

Figure S2. *High speed homogenizing zoomed picture*

Table S1. Reaction conditions for the formic acid treatment of sulphite softwood dissolving pulp for 24h.

Entry	Additive	Temp. (h)	Yield[a]	NFC
1	-	50	-	no
2[b]	-	90	-	no
3	-	80	98	yes
4	-	90	>99	yes
5[c]	-	90	97	yes
6	ammonium formiate	110	97	yes
7[d]	-	90	97	yes
8[e]	-	90	90	yes

(a) Yield of dry NFC foam material. (b) The pulp was treated with 50% formic acid solution. (c) The reaction was performed with recovered formic acid. (d) Homogenization time was 180 minutes. (e) Formic acid treatment was 48h and homogenization time 180 minutes.

Typical procedure for the large scale formic acid treatment of sulphite softwood dissolving pulp

To two wide opening media bottles (1 L) was poured concentrated formic acid (1 L). Next, sulphite softwood dissolving pulp (166.5 g, 15% consistency, 25 g dry mass of pulp) was added to each bottle and the mixtures were heated to 90 ^{o}C. After mechanical stirring the suspensions with a IKA RW 20 digital stirrer (2,400 rpm) for 24h, the reaction temperature was decreased to room temperature and next ultra sonicated in a bath for 1h using a BANDELIN SONOREX DIGITEC. Then the precipitates were diluted with water (4 L) and then neutralized to pH = 7 by NaOH (aq. 1 M). (Alternatively NaOH (1 M, 50 mL/g precipitate) was added to reach a pH between 13−14. After stirring for 1h, the suspension was neutralized (pH = 7) with HCl (1 M)). Afterwards the suspensions were centrifuged with 6,000 rpm for 10 min and the supernatant was decanted away. The precipitates were further washed by deionized water following centrifugation; this procedure was repeated three times. The purified materials were combined and homogenized with IKA T 25 ULTRA TURRAX High Speed Homogenizer (15,000 rpm) for 90 min. The average Mw was determined by SEC (M_w = 112 360 Da, PDI = 6.8) All of this material was subsequently freeze-dried as described above giving a 3D-foam material.

Typical procedure for the recycling of formic acid

To a round bottom flask (500 mL) was poured concentrated formic acid (200 mL). Next, sulphite softwood dissolving pulp (66.7 g, 15% consistency, 10 g dry mass of pulp) was added and the mixture was heated to 90 ^{o}C. After stirring the suspension with a mechanical IKA RW 20 digital stirrer (2,400 rpm) for 24h, the reaction temp. was decreased to room temperature and next ultra sonicated in a bath for 1h using a BANDELIN SONOREX DIGITEC. Subsequently, the reaction mixture was centrifuged with 6,000 rpm for 10 min and the formic acid was decanted. This formic acid (200 mL) was next reused for a second round of NFC fabrication. The precipitates from the above sequences were washed using deionized water (800 mL) and then neutralized to pH = 7 by NaOH (aq. 1M). (Alternatively NaOH (1 M, 50 mL/g percipitate) was added to reach a pH between 13−14. After stirring for 1h, the suspension was neutralized (pH = 7) with HCl (1 M). Next, the reaction mixture was centrifuged with 6,000 rpm for 10 min and the water was decanted. The precipitate was further diluted with water and centrifuged; this procedure was repeated three

times with IKA T 25 ULTRA TURRAX High Speed Homogenizer (15,000 rpm) for 90 min. After this, the resulting NFC material was freeze-dried to a foam material as described above.

General procedure for the NFC processing using TEMPO-NaClO-oxidation-homogenization method

The processing of wood pulp-derived nanofibrillated cellulose (NFC) using 2, 2, 6, 6-tetramethypiperidine-1-oxyl (TEMPO)-NaClO-oxidation method combined with mechanical shearing of pulp. TEMPO/NaClO-oxidation was done on cellulose sulphite softwood dissolving pulp according to the method described by Saito et al.[2] Here the pulp was suspended in deionized water (100 mL/g pulp) containing TEMPO ((0.016 g, 0.1 mmol)/g pulp) and sodium bromide ((0.1 g, 1 mmol)/g pulp). The dosage of NaClO used in this trial was 10 mmol of NaClO per gram of cellulose pulp. The pH was kept at approximately 9–10 by adjusting it with either NaOH or HCl and the reaction time for the chemical oxidation was 2h. The reaction was stopped by washing with excess deionized water (more than 100 L). A T25 Ultra Turrax high-shear homogenizer (IKA Works, Wilmington, NC, USA) was used in performing the homogenization. The machine was set at 15,000 rpm and mechanical treatment time for each homogenization was 90 min per 1L of oxidized pulp suspension.

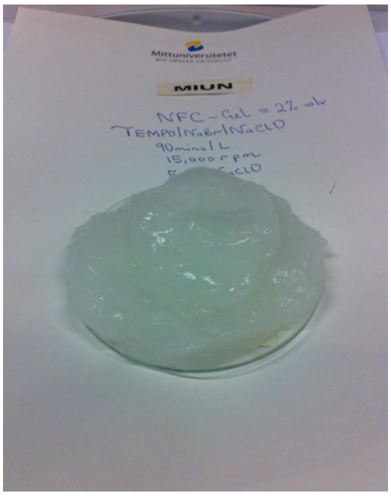

Figure S3. *NFC-gel (2%w/v) after TEMPO-NaClO-oxidation-homogenization processing*

Freeze-drying of the NFC-gel

25 g, 50 g, or 100 g of TEMPO-oxidized NFC-gel were put in beakers and placed in liquid nitrogen at temperature less than -198 ^{o}C before freeze-drying. Liquid nitrogen

was preferred as opposed the normal freezer, as it is a fast-freezing method and this method helps in preserving the pore size distribution of the 3D-structure (Figure S4).

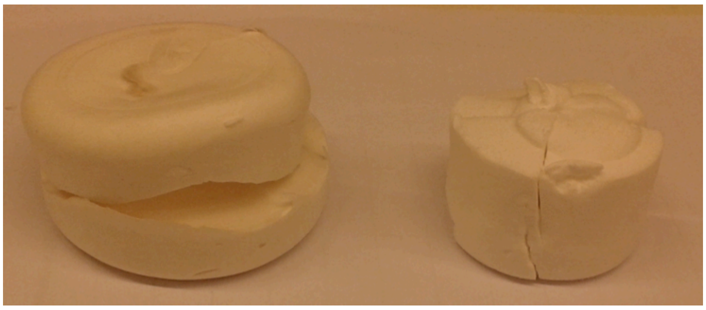

Figure S4. Foam material from freeze-dried NFC: The left foam material is NFC produced using the TEMPO method. The right foam material is NFC derived from the formic acid method.

Typical procedure for the tandem catalytic aerobic oxidation/formic acid treatment of sulphite softwood dissolving pulp

To a round bottom flask (500 mL) containing sulphite softwood dissolving pulp (16.7 g, 15% consistency, 2.5 g dry sample) and deionized water (50 mL) was subsequently added TEMPO (32 mg, 0.2 mmol), $NaNO_2$ (45 mg, 0.66 mmol) and HCl (121 mg, 1.3 mmol, 37% HCl). This mixture was further heated to 60 ^{o}C and stirred using IKA RW 20 digital with 2,400 rpm for 24h.[2,3] Next, formic acid (50 mL) was added and the temperature was increased to 90 ^{o}C. After stirring for 24h, the reaction mixture was diluted with water (800 mL) and then neutralized to pH = 7 by NaOH (aq. 1 M). Afterwards the reaction mixture was centrifuged with EBA 21 Hettich ZENTRIFUGEN with 6,000 rpm for 10 min and the water decanted away. The solid precipitate was further washed by diluting it in deionized water followed by additional centrifugation (this procedure was repeated three times). The material was further homogenized with IKA T 25 ULTRA TURRAX High Speed Homogenizer with 15,000 rpm for 90 min.

General procedure for basic treatment of all formic acid derived NFC or foam materials used in the functionalization experiments: Removal of formic acid ester groups

To a round bottom flask (500 mL) charged with formic acid derived NFC foam or material, was added a NaOH (1 M, 50 mL/g NFC-foam or solid)) at room

temperature. After stirring for 1h, the suspension was neutralized (pH = 7) with HCl (1 M). Centrifugation of this suspension at 6,000 rpm for 10 min was followed by decantation of the supernatant. The precipitate was further washed with deionized water to give the NFC in quantitative yield. This material was freeze-dried (lyophilized) according to the above procedure to provide NFC foam in quantitative yield. IR-analysis determined that all formic ester groups were gone.

Typical procedure 1 for the silylation of NFC

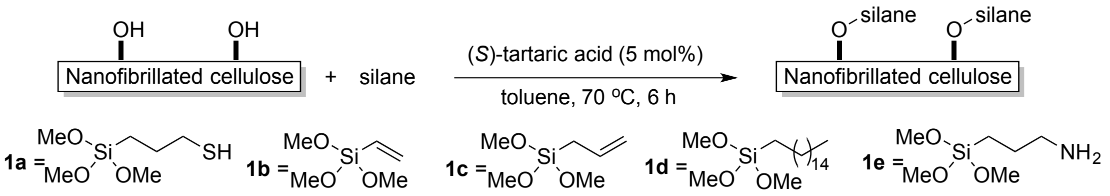

To a round-bottomed flask (250 mL) charged with NFC foam material (600 mg, 3.7 mmol, 1 equiv), which had undergone basic work-up, was added subsequently toluene (100 mL), (*S*)-tartaric acid (75 mg, 0.5 mmol, 14 mol %,) and silane **1** (1 g). Next, this reaction mixture was heated to 70 °C and stirred. After 6h, the reaction temperature was decreased and the **1**-modified NFC, filtrated and washed with CH_2Cl_2 (200 mL). The washed NFC was further treated by Soxhlet extraction using CH_2Cl_2. After Soxhlet extraction for 24h, the modified NFC was dried under reduced pressure.

Table S2. Elemental analysis of the silylated NFC derived by formic acid method or TEMPO-NaClO-oxidation method.

Silane	Element	C	H	Si	S	N
**1a**[a]	% Found	37.18	5.28	0.62	0.87	-
**1a**[a],[b]	% Found	42.16	6.04	0.30	<0.10	-
**1a**[c]	% Found	38.61	5.59	1.58	0.83	-
**1c**[a]	% Found	41.92	5.96	0.60	-	-
**1c**[c]	% Found	40.40	5.81	1.80	-	-
**1d**[a]	% Found	37.83	5.40	0.17	-	-
**1d**[c]	% Found	37.12	5.66	0.13	-	-
**1e**[a]	% Found	40.56	6.28	6.60	-	1.76
**1e**[c]	% Found	37.72	5.34	5.53	-	1.90

(a) Formic acid fabricated NFC. (b) The silylation was performed without acid. (c) NFC derived from TEMPO-NaClO oxidation-homogenization route

Procedure for silylation of NFC with 1a and 1c and it subsequent UV-treatment under "click" conditions

To a round-bottomed flask (250 mL) charged with NFC foam material (600 mg, 1.0 equiv.) was added toluene (100 mL). Next, (*S*)-tartaric acid (75 mg, 0.5 mmol, 14 mol %), mercaptopropyltrimethoxysilane **1a** (1.0 g, 5.1 mmol, 1.4 equiv.) and allyltrimethoxysilane **1c** (0.83 g, 5.1 mmol, 1.4 equiv.) were added and the reaction mixture was heated to 70 ^{o}C. After stirring for 6h, the modified NFC was filtrated off and washed with CH_2Cl_2 (200 mL). Next, Soxhlet extraction using CH_2Cl_2 was followed by drying the resulting **1a**-**1c**-NFC under reduced pressure.

To a mixture of silylated NFC (300 mg) in a minimum amount of DMF was added 2,2-dimethoxy-2-phenylacetophenone (DMPA) (1 wt.-%, 3.0 mg). Then the reaction was irradiated with UV-lamp (UV-B bulb, TL20W/12, 20W) for 1 h. Afterwards, the cross-linked NFC was extracted (Soxhlet) with dichloromethane Then the material was dried under vacuum.

Table S3a. Elemental analysis of "click" reacted **1a-1c** modified NFC.

Entry	Element	C	H	Si	S	Ratio (Si:S)[c]
1[a]	% Found	37.55	5.27	0.62	<0.10	>7.1:1
2[b]	% Found	39.25	5.51	0.69	<0.10	>7.9:1

(a) Formic acid fabricated NFC. (b) NFC derived from TEMPO-NaClO oxidation-homogenization route. (c) Mol ration (Si:S).

Table S4. Data of the NFC foam materials.

Material	S_{BET}[a] (m^2g^{-1})	P_{BJH}[b] (Å)	V_{BJH}[c] (cm^3g^{-1})
NFC foam material[d]	6.66	91.8	**0.015**: Single point adsorption total pore volume of pores less than 1649.077 Å width at p/p° = 0.988240468
NFC foam material[e]	6.78	162	**0.027**: Single point adsorption total pore volume of pores less than 1236.682 Å width at p/p° = 0.984238134
NFC foam from material[f]	19.3	110	**0.053**: Single point adsorption total pore volume of pores less than 1493.2012 Å width at p/p° = 0.986870096
AmP-NFC[g]	9.32	111	**0.026**: Single point adsorption total pore volume of pores less than 1410.437 Å width at p/p° = 0.986214373
AmP-NFC[h]	10.0	109	**0.027**: Single point adsorption total pore volume of pores less than 1601.918 Å width at p/p° = 0.987888637
NFC-**1d**[i]	7.02	77.7	**0.014**: Single point adsorption total pore volume of pores less than 1761.181 Å width at p/p° = 0.989000161
NFC-**1a**[j]	8.84	64.6	**0.014**: Single point adsorption total pore volume of pores less than 1830.059 Å width at p/p° = 0.989420163
Click reacted **1a**-**1c**-NFC[k]	7.53	69.2	**0.013**: Single point adsorption total pore volume of pores less than 1841.974 Å width at p/p° = 0.989489588
Click reacted **1a**-**1c**-NFC[l]	7.90	83.4	**0.016**: Single point adsorption total pore volume of pores less than 1792.391 Å width at p/p° = 0.989194530
AmP-NFC-Pd(0)[m]	49.2	101	**0.012**: Single point adsorption total pore volume of pores less than 1716.778 Å width at p/p° = 0.988711293

(a) S_{BET}: specific surface area was calculated by the Brunauer-Emmett-Teller (BET) equation. (b) P_{BJH}: Adsorption average pore width was calculated by the Barret-Joyner-Halenda (BJH) equation. (c) V_{BJH}: pore volumes were calculated by the Barret-Joyner-Halenda (BJH) equation. (d) Formic acid fabricated NFC at 90 °C. (e) Formic acid fabricated NFC at 80 °C. (f) NFC derived from TEMPO-NaClO oxidation-homogenization route. (g) AmP-NFC from formic acid fabricated NFC at 90 °C. (h) AmP-NFC with NFC derived from TEMPO-NaClO oxidation-homogenization route. (i) Silylation with silane **1d** of NFC from formic acid fabricated at 90 °C. (j) Silylation with silane **1a** without acid of NFC from formic acid fabricated at 90 °C. (k) Silylation with silane **1a** and **1d** of NFC from formic acid fabricated at 90 °C and then further UV-treatment. (l) Silylation with silane **1a** and **1d** of NFC derived from TEMPO-NaClO oxidation-homogenization route and then further UV-treatment. (m) Pd complexation of AmP-NFC from formic acid fabricated NFC at 90 °C.

Typical procedure 2 for the silylation of NFC.

To a flame-dried round-bottomed flask (50 mL) charged with NFC foam material (200 mg, 1.0 equiv.) was added subsequently (*S*)-tartaric acid (9 mg, 0.06 mmol, 5

mol %), toluene (4 mL) and silane **1** (3.2 mmol, 2.6 equiv.). Next, the flask was placed in an oil bath and heated to 80 °C. After stirring 6h, the reaction temperature was decreased to room temperature and the **1**-modified NFC was washed by Soxhlet extraction using CH_2Cl_2. After Soxhlet extraction for 24h, the modified NFC was dried under reduced pressure.

Typical procedure 2 for the silylation of NFC with 1a and 1c.

To a flame-dried round-bottomed flask (50 mL) charged with NFC foam material (200 mg, 1.0 equiv.) was added subsequently (*S*)-tartaric acid (9 mg, 0.06 mmol, 5 mol %), toluene (4 mL), silane **1c** (3.2 mmol, 2.6 equiv.) and silane **1a** (3.2 mmol, 2.6 equiv.). Next, the flask was placed in an oil bath and heated to 80 °C. After stirring 6h, the reaction temperature was decreased to room temperature and the **1**-modified NFC was washed by Soxhlet extraction using CH_2Cl_2. After Soxhlet extraction for 24h, the modified NFC was dried under reduced pressure.

Table S3b. Elemental analysis of NFC modified with silane **1a** and **1c** using procedure 2.

Entry	Element	C	H	Si	S	Ratio (Si:S)[d]
1[a,b]	% Found	37.35	5.65	3.42	1.97	2.0:1.0
2[a,c]	% Found	38.23	5.59	2.29	3.19	1.0:1.2

(a) Formic acid fabricated NFC. (b) 1:1 ratio of silane **1a** and **1c** was employed. (c) 6:1 ratio of silane **1a** and **1c** was employed. d) Mol ratio (Si:S).

Procedure for Lipase-catalyzed synthesis of 6-mercaptohexan-1-ol-initiated poly(ε-caprolactone) or 4- pentene-1-ol-initiated poly(ε-caprolactone).

To an oven-dried vial (24 mL) containing caprolactone (1.14 g, 10.0 mmol, 1.0 equiv.) were added 6-mercaptohexan-1-ol (53.6 mg, 0.4 mmol, 4 mol%) or 4-pentene-1-ol (35 mg, 0.4 mmol, 4 mol%) and Novozyme 435 (50 mg, 120 mg/mmol) and the reaction was heated to 80 °C.[5] After stirring for 24 hours, the reaction temperature was decreased, the reaction mixture was dissolved in CH_2Cl_2 and filtered. Next, the solution was added to a solution of cold methanol and the alcohol initiated

poly(ε-caprolactone) (PCL) precipitated. After filtration, a white solid was isolated and dried under reduced pressure providing the corresponding alcohol-initiated PCL. ^{1}H NMR and MALDI-TOF analysis of the isolated 6-mercaptohexan-1-ol initiated PCL (0.95 g) revealed that only the alcohol group had been esterified and that the thiol was intact.

6-Mercaptohexan-1-ol-initiated-PCL: White solid; **^{1}H NMR (500 MHz, $CDCl_3$)**: δ 4.06 (t, *J* = 6.7 Hz, 38H), 3.64 (t, *J* = 6.7 Hz, 2H), 2.52 (q, *J* = 7.4, 1.1, 2H), 2.3 (t, *J* = 7.4Hz, 38H), 1.64 (m, 80H), 1.37 (m, 42H); **^{13}C NMR (125.8 MHz, $CDCl_3$)**: δ 173.5, 64.2, 64.1, 62.6, 34.1, 33.8, 32.3, 28.3, 27.9, 25.5, 25.3, 24.5.

4- Pentene-1-ol-initiated-PCL: White solid; **^{1}H NMR (500 MHz, $CDCl_3$)**: δ 5.78 (m, 1H), 5.02 (dd, *J* = 17.8, 1.8 Hz, 1H), 4.98 (dd, *J* = 10,1.8 Hz, 1H), 4.05 (t, *J* = 6.8 Hz, 49H), 3.63 (t, *J* = 6.5 Hz, 2H), 2.30 (t, *J* = 7.5 Hz, 49H), 2.09 (q, *J* = 7.2 Hz, 2H), 1.65 (m, 95H), 1.40 (m, 48H); **^{13}C NMR (125.8 MHz, $CDCl_3$)**: δ 173.5, 137.4, 115.2, 64.1, 63.7, 62.6, 34.1, 32.3, 30.0, 28.3, 27.8, 25.5, 25.3, 24.5.

Typical procedure for the (*S*)-tartaric acid-catalyzed synthesis of alcohol-initiated poly(δ-valerolactone) (PVL)

To an oven dried vial (24 mL) containing δ-valerolactone (VL) (0.75 g, 7.5 mmol, 1.0 equiv.) were added 6-mercaptohexan-1-ol (40 mg, 0.3 mmol, 4 mol%), (*S*)-tartaric acid (60 mg, 0.4 mmol, 5 mol%) and the reaction was heated to 110 °C.[6] After stirring for 1h, the temperature was decreased; the reaction mixture was dissolved in CH_2Cl_2 and filtered. Next, the solution was added to a cold methanol (–20 °C) and a white precipitate was formed. After filtration and drying under reduced pressure, a white solid was formed. ^{1}H NMR and MALDI-TOF analysis of the isolated 6-mercaptohexan-1-ol-initiated PVL (0.69 g) revealed that only the alcohol group had been esterified and that the thiol was intact.

6-Mercaptohexan-1-ol initiated PVL: White solid; **^{1}H NMR (500 MHz, $CDCl_3$)**: δ 4.06 (t, *J* = 6.2 Hz, 42H), 3.64 (t, *J* = 6.3 Hz, 2H), 2.52 (q, *J* = 7.4, 11.0, 2H), 2.32 (t, *J* = 6.8 Hz, 45H), 1.64 (m, 96H); **^{13}C NMR (125.8 MHz, $CDCl_3$)**: δ 173.2, 63.9, 62.2, 33.7, 32, 28.5, 27.9, 25.4, 24.4, 21.4, 21.1.

4- Pentene-1-ol-initiated-PVL: White solid; **^{1}H NMR (500 MHz, $CDCl_3$)**: δ 5.78 (m, 1H), 5.02 (dd, *J* = 17, 1.6 Hz, 1H), 4.96 (dd, *J* = 10.0, 1.3 Hz, 1H), 4.04 (t, *J* = 6.0 Hz, 42H), 3.63 (t, *J* = 6.4 Hz, 2H), 2.3 (t, *J* = 7.2Hz, 42H), 1.64 (m, 91H); **^{13}C NMR (125.8 MHz, $CDCl_3$)**: δ 173.5, 137.4, 115.2, 63.8, 63.8, 62.1, 33.8, 33.7, 32.0, 30.0, 27.8, 21.5, 21.4, 21.1.

Typical procedure for lipase-catalyzed alcohol-end group esterification of 6-mercaptohexan-1-ol-initiated-PCL and 4-Pentene-1-ol-initiated-PCL with nonanoic acid:

To an oven dried vial (24 mL) containing 6-mercaptohexan-1-ol-initiated-PCL or 4-Pentene-1-ol-initiated-PCL (0.1 mmol, 1.0 equiv.) and Molecular sieves 4Å (100 mg) were added nonanoic acid (32 mg, 0.2 mmol, 2.0 equiv.) and Novozyme 435 (25 mg, 120 mg/ mmol). The vial was sealed and the reaction mixture was heated to 80 ^oC.[5] After stirring for 16h, the reaction allowed to reach room temperature, CH_2Cl_2 was added and the Novozyme 435 particles was removed by filtration using celite. The filtrate was added to a cold methanol solution and a white precipitate was formed. Next, filtration and drying of the white solids under reduced pressure gave the corresponding nonanoic acid-terminated PCLs as white solids. ^{1}H NMR analysis of the isolated nonanoic acid-terminated 6-mercaptohexan-1-ol-initiated PCL revealed that only the alcohol end-group had been esterified and that the thiol was intact.[5]

Nonanoic acid-terminated-6-mercaptohexan-1-ol-initiated-PCL: White solid; **^{1}H NMR (500 MHz, $CDCl_3$)**: δ 4.05 (t, *J* = 7.2 Hz, 160H), 2.85 (t, *J* = 7.2 Hz, 2H), 2.67 (t, *J* = 7.2, 2H), 2.54 (t, *J* = 7.3 Hz, 2H), 2.3 (t, *J* = 7.6Hz, 165H), 1.64 (m, 349H), 1.38 (m, 171H), 1.27 (m, 63H), 0.87 (t, *J* = 7.5Hz, 16H); **^{13}C NMR (125.8 MHz, $CDCl_3$)**: δ 173.5, 64.1, 64.0, 34.3, 34.1, 31.8, 29.2, 29.1, 28.3, 25.5, 25.0, 24.5, 22.6, 14.0.

Nonanoic acid-terminated-4- Pentene-1-ol-initiated PCL: White solid; **^{1}H NMR (500 MHz, $CDCl_3$)**: δ 5.78 (m, 1H), 5.02 (dd, *J* = 17.8, 1.8 Hz, 1H), 4.98 (dd, *J* = 10,1.8 Hz, 1H), 4.05 (t, *J* = 6.8 Hz, 83H), 2.30 (t, *J* = 7.5 Hz, 86H), 2.1 (q, *J* = 7.2 Hz, 2H), 1.63 (m, 170H), 1.37 (m, 77H), 1.27(m, 32H), 0.8(t, *J* = 6.6 Hz, 10H); **^{13}C NMR (125.8 MHz, $CDCl_3$)**: δ 173.4, 137.4, 115.26, 64.1, 34.3, , 31.7, 30.0, 29.2, 29.1, 29.0, 27.8, 24.9, 24.6, 22.6, 14.0.

Procedure for the thiol-ene 'click' reaction between 1c-NFC and 6-Mercaptohexan-1-ol initiated PVL, 6-Mercaptohexan-1-ol initiated PCL or Nonanoic acid-terminated-6-mercaptohexan-1-ol-initiated-PCL.

To a scintillation vial (20 mL) containing allyl-**1c**-NFC foam material (30 mg) and PCL or PVL with a free thiol-end-group (30 mg), was added THF (0.5 mL) and DMPA (3–6 mg). Next, the reaction was irradiated for 24h using an UV-lamp (UV-B bulb, TL20W/12, 20 W). The UV-treated NFC material was removed and next extracted using a Soxhlet extractor and acetone as the solvent for 17h. The extracted NFC foam material was subsequently dried overnight under reduced pressure.

Table S5. Elemental analysis of thiol-ene click reaction between allyl-**1c**-NFC and 6-mercaptohexan-1-ol initiated PCL.

Entry	Element	C	H	Si	S
1	% Found	40.34	5.46	0.84	<0.10

Procedure for the thiol-ene 'click' reaction between allyl-1c-NFC and 1-octanethiol

To a scintillation vial (20 mL) containing **1c**-NFC (30 mg) and octanethiol (293 mg, 2.0 mmol) was added DMPA (3 mg) and the reaction mixture was irradiated with an UV-lamp (UV-B bulb, TL20W/12, 20 W) for 24h. Next, the UV treated NFC material was removed and extracted with a Soxhlet extractor with acetone for 16h. The extracted NFC foam material was subsequently dried overnight under reduced pressure.

Procedure for the thiol-ene 'click' reaction between TPSi-1a-NFC and 4-Pentene-1-ol-initiated PCL, 4- Pentene-1-ol-initiated PVL or nonanoic acid 4-pentene-1-ol-initiated PCL

To a scintillation vial (20 mL) containing TPSi-**1a**-NFC foam material (30 mg) and PCL or PVL with a free terminal olefin-end-group (30 mg), was added THF (0.5 mL) and DMPA (3 mg). Next, the reaction was irradiated for 24h using an UV-lamp (UV-B bulb, TL20W/12, 20 W). The UV-treated NFC material was removed and next extracted using a Soxhlet extractor and acetone as the solvent for 17h. The extracted NFC foam material was subsequently dried overnight under reduced pressure.

Procedure for the thiol-ene 'click' reaction between TPSi-1a-NFC or 1a-1c-NFC and Quinidine or Quinine

To a scintillation vial (20 mL) containing TPSi-**1a**-NFC foam material (30 mg) or **1a**-**1c**-NFC foam material (30 mg) and Quinidine or Quinine (30 mg, 0.1 mmol) in DMF (0.5 mL) was added DMPA (2 mol%). Next, the reaction was irradiated for 24h using an UV-lamp (UV-B bulb, TL20W/12, 20 W). The UV-treated NFC material was removed and stirred in aqueous solution (adjusted to pH 10 by NaOH 1N) for 1 h. Next, the mixture was neutralized to pH 6–7 using HCl (1N). Afterwards the material was washed with water and acetone by centrifugation and then dried under vacuum. The washed NFC foam material was subsequently dried overnight under reduced pressure. The cinchona-alkaloid-modified NFC exhibits high UV-activity and was fluorescent (Figures S5, S6 and S7). Performing the same procedure with NFC without addition of DMPA did not give a NFC material with UV activity (Blank).

Table S6. Elemental analysis of Quinidine-TPSi-**1a**-NFC.

Entry	Element	C	H	Si	N
1[a]	% Found	42.22	6.18	0.94	0.58
2[b]	% Found	39.07	5.67	1.23	0.50

(a) Formic acid fabricated NFC. (b) NFC derived from TEMPO-NaClO oxidation-homogenization route

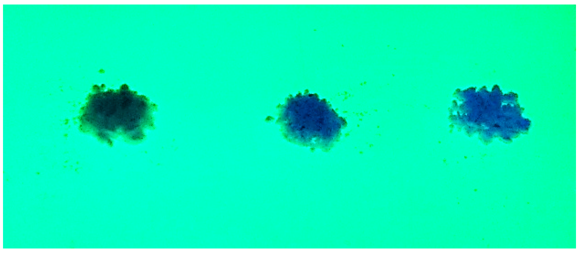

Figure S5. Modified NFC under a UV-lamp (Short wave length): Left: TPSi-**1a**- NFC (blank). Middle: Quinidine-TPSi-NFC. Right: Quinidine-TPSi-NFC.

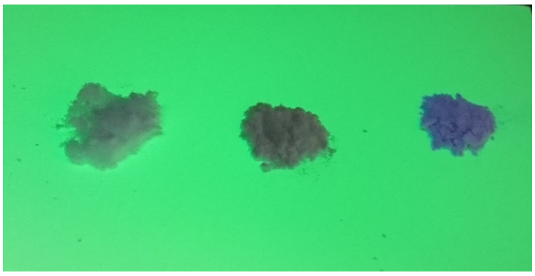

Figure S6. Modified NFC under a UV-lamp (Short wave length): Left: NFC. Middle: **1a**-**1c**-NFC. Right: Quinididine-**1a**-**1c**-NFC.

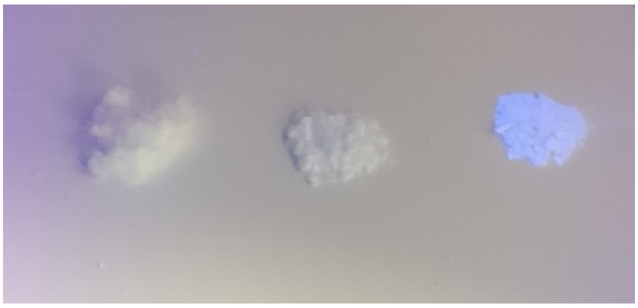

Figure S7. UV-lamp (long wave length): Left: NFC. Middle: **1a**-**1c**-NFC. Quinidine-**1a**-**1c**-NFC

Procedure for the synthesis of NFC-AmP-Pd(0) heterogeneous catalyst

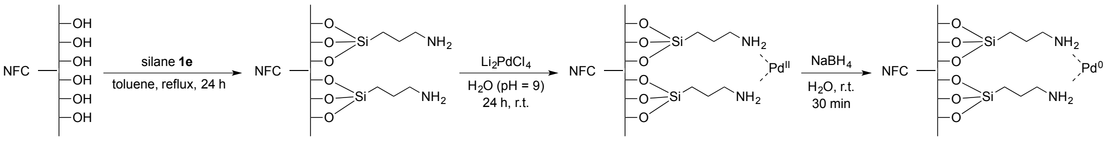

Step 1.

To a flask (250 mL) containing formic acid derived NFC foam material (0.4 g), which had undergone basic treatment, were added 3-aminopropyl-trimethoxysilane (AmP) **1e** (1.2 mL), (*S*)-tartaric acid (80 mg) and dry toluene (8 mL). After stirring the reaction mixture at 80 °C for 6 h, the **1e**-NFC foam was filtrated and extracted with acetone (soxhlet) overnight. Next, the NFC material was dried under vacuum, giving a white powder (0.45 g).

Step 2.

The aminofunctionalized Amp-**1e**-NFC (AmP-NFC) (200 mg) was suspended in a pH = 9 adjusted deionized water solution (10 mL). Li_2PdCl_4 (189 mg,) was solubilized in

a pH = 9 adjusted deionized water (6 ml) solution and then added to the AmP-NFC suspension. The reaction was stirred at r.t for 24 h. The reaction mixture was washed with water and acetone (3 times) and dried under vacuum, giving the product AmP-NFC-Pd(II) as brown powder (0.34 mg).

Step 3.

The AmP-NFC-Pd(II) (200 mg) was suspended in water (8 mL) and a solution of $NaBH_4$ (135 mg) in water (5 ml) was added slowly. The reaction was stirred at r.t for 30 min and then washed with water and acetone (3 times), followed by drying under vacuum over night, giving the product AmP-NFC-Pd(0) as grew powder (170 mg). The elemental analysis showed that the Pd content on the AmP-NFC-Pd(0) is 7.77 wt.%.

Procedure for the AmP-NFC-Pd(0)-catalyst catalyzed Suzuki-Miyaura reaction

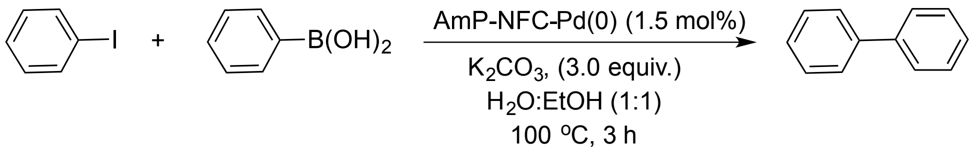

A microwave vial equipped with a magnetic stir bar was charged with AmP-NFC-Pd(0)-catalyst (4.2 mg, 0.0075 mmol, 1.5 mol%), phenyl boronic acid (73.2 mg, 0.6 mmol, 1.2 equiv.), K_2CO_3 (207.3 mg, 1.5 mmol, 3.0 equiv.), followed by addition of H_2O:EtOH (1:1, 1.5 mL). Subsequently, Iodobenzene (102.0, 0.5 mmol, 1.0 equiv.) was added and the reaction mixture heated to 100 °C and stirred for 3 h. Next, the reaction mixture was centrifuged and the solid diluted with acetone (3x10 mL) and centrifuged and then concentrated before purification or directly subjected to flash chromatography on silica (petroleum ether/EtOAc 100–90%) affording the pure products (96% yield).

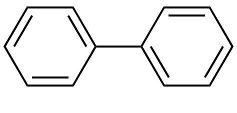

1,1'-biphenyl;[7] White solid. **^{1}H NMR (400MHz, $CDCl_3$):** δ 7.64 (d, J = 8.1 Hz, 4H), 7.48 (t, J = 7.5 Hz, 4H), 7.64 (t, J = 7.5 Hz, 2H); **^{13}C NMR (100MHz, $CDCl_3$):** 141.4, 128.9, 127.4, 127.3.

Procedure for the recycling of the AmP-NFC-Pd(0)-catalyst catalyzed Suzuki-Miyaura reaction

A microwave vial equipped with a magnetic stir bar was charged with AmP-NFC-Pd(0)-catalyst (8.4 mg, 0.015 mmol, 1.5 mol%), phenyl boronic acid (146.4 mg, 1.2 mmol, 1.2 equiv.), K_2CO_3 (414.6 mg, 3.0 mmol, 3.0 equiv.), followed by addition of H_2O:EtOH (1:1, 3.0 mL). Subsequently, Iodobenzene (204.01, 1.0 mmol, 1.0 equiv.) was added and the reaction mixture heated to 100 °C and stirred for 3 h. Next, the reaction mixture was centrifuged and the solid diluted with acetone (3x10 mL) and centrifuged. The collected was concentrated and purified by flash chromatography on silica (petroleum ether/EtOAc 100–90%) affording the pure products. The solid catalyst was diluted with water (10 mL) in order to remove remaining base and centrifuged. The solid catalyst was further diluted with acetone (2x10 mL) and centrifuged. Afterwards the solid heterogeneous catalyst was dired under vacuum and the further used in next cycle.

Table S7. Recycling study of the AmP-NFC-Pd(0)-catalyst catalyzed Suzuki-Miyaura reaction

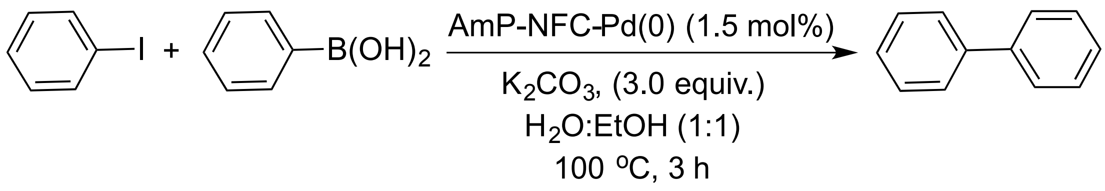

Cycle[a]	Yield (%)[b]
1	95
2	97
3	89
4	85

(a) Reaction conditions: AmP-NFC-Pd(0) (8.4 mg, 1.5 mol%), Iodobenzene (204.01 mg, 1.0 mmol, 1.0 equiv.), phenyl boronic acid (146.4 mg, 1.2 mmol, 1.2 equiv.), K_2CO_3 (414.6 mg, 3.0 mmol, 3.0 equiv.), solvent (3 mL), 100 °C, 3 h. (b) Yield of purified product after silica-gel column chromatography.

Typical procedure for the hot-filtration test

A microwave vial equipped with a magnetic stir bar was charged with AmP-NFC-Pd(0)-catalyst (4.2 mg, 0.0075 mmol, 1.5 mol%), phenyl boronic acid (73.2 mg, 0.6 mmol, 1.2 equiv.), K_2CO_3 (207.3 mg, 1.5 mmol, 3.0 equiv.), followed by addition of H_2O:EtOH (1:1, 1.5 mL). Subsequently, iodobenzene (102.0, 0.5 mmol, 1.0 equiv.) was added and the reaction mixture heated to 100 ^oC. The NFC-AmP-Pd(0)-catalyst was removed through centrifugation after 35% conversion was reached and the solid free filtrate was allowed to stir for 24 h under the same reaction conditions. Analysis of the reaction mixture showed that no further conversion of the substrate had occurred and elemental analysis of the reaction mixture showed that 3.9 ppm Pd had leached to the reaction.

General procedure for the acid catalyzed screening of allyltrimethoxysilylation 1c to 3-phenylpropionalcohol:

To an oven dried vial (8 mL) containing 3-phenylpropionalcohol (68.1 mg, 0.5 mmol, 1.0 equiv.) in toluene (1.0 mL) were added allyltrimethoxysilane **1c** (162.3 mg, 1.0 mmol, 2.0 equiv.) and acid (0.05 mmol, 10 mol%). The reaction was heated to 70 ^oC and monitored by Gas chromatography analysis.

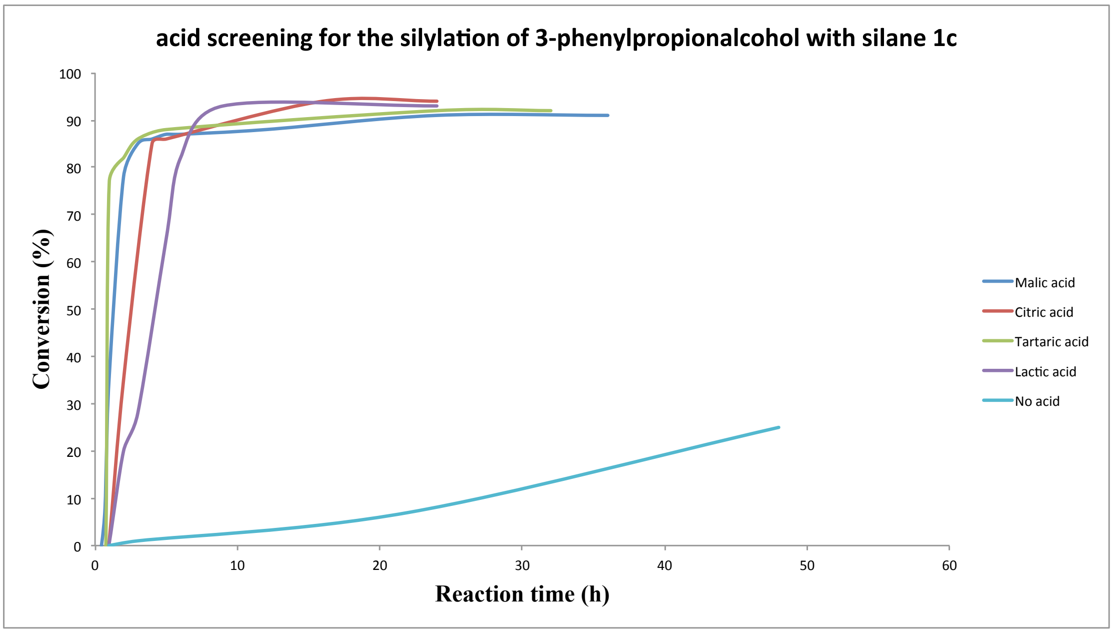

Graf S1. *Acid screening for the silylation with silane* ***1c*** *and 3-phenylpropionalcohol*

General procedure for the silylation of 3-phenylpropionalcohol with 1a:

To an oven dried vial (8 mL) containing 3-phenylpropionalcohol (68.1 mg, 0.5 mmol, 1.0 equiv.) in toluene (1.0 mL) were added silane **1a** (1.0 mmol, 2.0 equiv.) and in the case of acid (*S*)-tartaric acid (7.5 mg, 0.05 mmol, 10 mol%). The reaction was heated to 70 °C and monitored by Gas chromatography analysis.

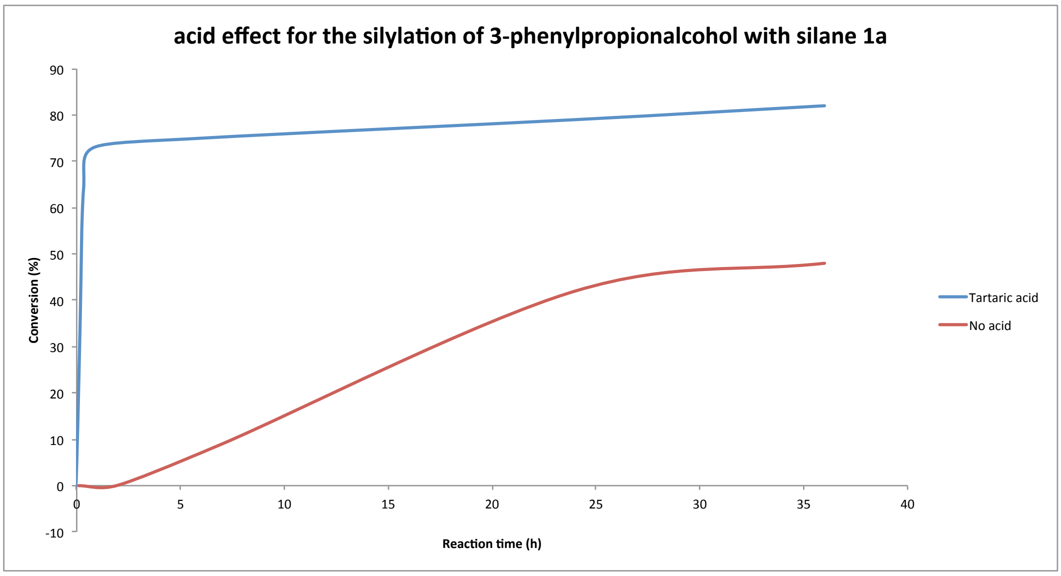

Graf S2. *Acid screening for the silylation with silane **1c** and 3-phenylpropionalcohol*

Allyldimethoxy(3-phenylpropoxy)silane

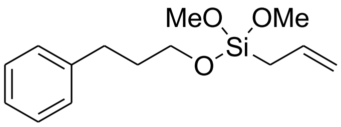

Colorless oil; **^{1}H NMR (500MHz, CDCl$_3$):** δ 7.35-7.27 (m, 2H), 7.25-7.17 (m, 3H), 5.93-5.81 (m, 1H), 5.10-5.01 (m, 1H), 5.01-4.94 (m, 1H), 3.87-3.79 (m, 2H), 3.61 (s, 6H), 2.76-2.70 (m, 2H), 1.98-1.87 (m, 2H), 1.76-1.68 (m, 2H); **^{13}C NMR (125MHz, CDCl$_3$):** δ 142.0, 132.4, 128.6, 128.4, 125.9, 115.1, 62.4, 50.8, 34.1, 32.1, 17.5; **HRMS (ESI$^+$) [M+Na]$^+$** calcd for $C_{14}H_{22}O_3SiNa^+$: 289.1230, found: 289.1235;

3-(dimethoxy(3-phenylpropoxy)-propane-1-thiol

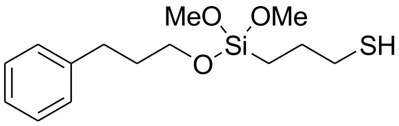

Colorless oil; **^{1}H NMR (500MHz, CDCl$_3$):** δ 7.33-7.26 (m, 2H), 7.24-7.17 (m, 3H),

3.83-3.76 (m, 2H), 3.58 (m, 6H), 2.75-2.67 (m, 2H), 2.56 (q, J = 14.9, 7.4 Hz, 2H), 1.97-1.85 (m, 2H), 1.79-1.70 (m, 2H), 1.38-1.31 (m, 1H), 0.81-0.73 (m, 2H); **^{13}C NMR (125MHz, $CDCl_3$):** δ 142.0, 128.6, 128.5, 125.9, 62.2, 50.7, 34.2, 32.1, 27.7, 27.7, 8.7; **HRMS (ESI$^+$) [M+Na]$^+$** calcd for $C_{14}H_{24}O_3SSiNa^+$: 323.1108, found: 323.1118.

Procedure for the thiol-ene 'click' reaction between (3 mercaptopropyl)trimethoxysilane 1a and Quinidine[7]:

A dry vial (12 mL) was charged with Quinidine (50 mg, 0.15mmol, 1equiv.), (3-mercaptopropyl) trimethoxysilane **1a** (27 µl, 0.15mmol, 1equiv.), DMPA (0.8 mg, 0.003 mmol, 2 mol%) and DMF or CH_2Cl_2 (0.2 ml). Next, the mixture was irradiated with UV-lamp (UV-B bulb, TL20W/12, 20 W) for 24h. ^{1}H NMR analysis confirmed that the corresponding thiol-ene silane-quinidine product had been formed.[8]

General procedure for the acid screening of silylated filter paper:

To a flask containing filter paper (500 mg, 1 equiv.), was added acid (10 wt.%, 50 mg) and toluene (7 mL). Next, silane **1a** (8.1 mmol, 2.9 equiv.) in toluene (3 mL) was added and the flask was heated to 70 °C in an oil-bath. After stirring, for the time stated in Table S9, the paper was washed with CH_2Cl_2 (50 mL) and subsequently Soxhlet extracted using CH_2Cl_2 for 17h. Then the paper was dried overnight under vacuum.

Table S8. Acid screening for the silylation of filter paper with silane **1a**.

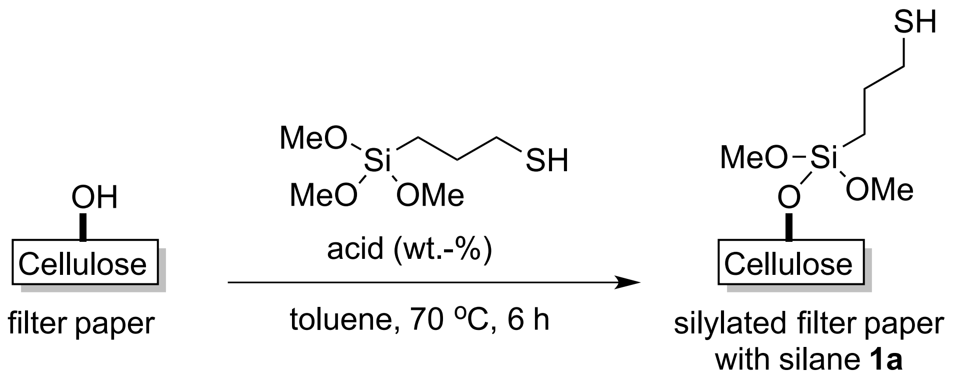

entry	acid(wt.-%)	angle
1[a]	-	109
2	Tartaric acid (10)	115
3	Citric acid (10)	113
4	*p*-Toluenesulfonic acid(1)	109
5	Malic acid (10)	115
6	Lactic acid (10)	106

[a]The reaction was run for 24 h.

General procedure for the silylation on filter paper:

To a flask containing filter paper (500 mg, 1 equiv), was added acid (10 wt.%, 50 mg), toluene (7 mL), followed by addition of silane **1** (8.1 mmol, 2.9 equiv.) in toluene (3 mL). The reaction was heated to 70 °C and stirred for the time stated in table S9. Afterwards the paper was washed with dichloromethane (50 mL) and extracted by Soxhlet using CH_2Cl_2. Then the paper was dried overnight under vacuum.

Table S9. Silylation of filter paper

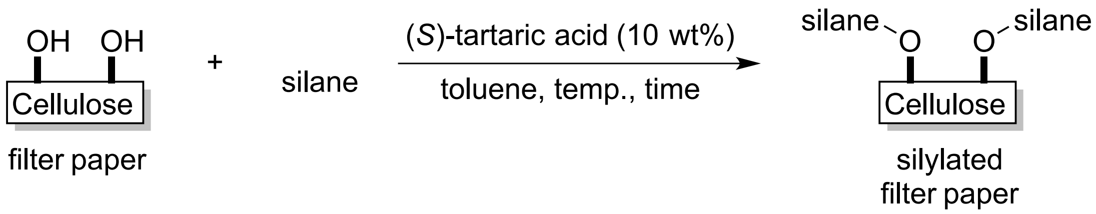

Entry	Silane	Time (h)	Temp. (°C)	Acid (10 wt.-%)	Angle
1	**1a**	24	70	-	109
2	**1a**	6	22	(*S*)-tartaric acid	105
3	**1a**	6	70	(*S*)-tartaric acid	115
4	**1b**	24	70	-	118
5	**1b**	6	70	(*S*)-tartaric acid	126
6	**1c**	6	70	-	123
7	**1c**	24	70	-	115
8	**1c**	6	70	(*S*)-tartaric acid	125
9	**1c**	24	70	(*S*)-tartaric acid	105
10	**1e**	24	70	(*S*)-tartaric acid	-

(a) The reaction was run for 24 h.

General procedure for the thiol-ene 'click' reaction between modified filter paper and alkene or thiol:

To a mixture of silylated filter paper (around 30 mg) and thiol or alkene (2.0 mmol) was added DMPA (1 wt.%, 4.5 mg). Then the reaction was irradiated with UV-lamp (UV-B bulb, TL20W/12, 20 W) for 1 h. Afterwards the filter paper was extracted (Soxhlet) with CH_2Cl_2. Then the paper was dried overnight under vacuum.

General procedure for the thiol-ene 'click' reaction between modified filter paper and polymer:

To a mixture of silylated filter paper (around 30 mg) and polymer (60 mg) in minimum amount of DMF was added DMPA (1 wt.%, 4.5 mg). Then the reaction was irradiated with UV-lamp (UV-B bulb, TL20W/12, 20 W) for 1 h. Afterwards the filter paper was extracted (Soxhlet) with dichloromethane. Then the paper was dried overnight under vacuum.

Table S10. Click reaction of filter paper modified with silane **1a**

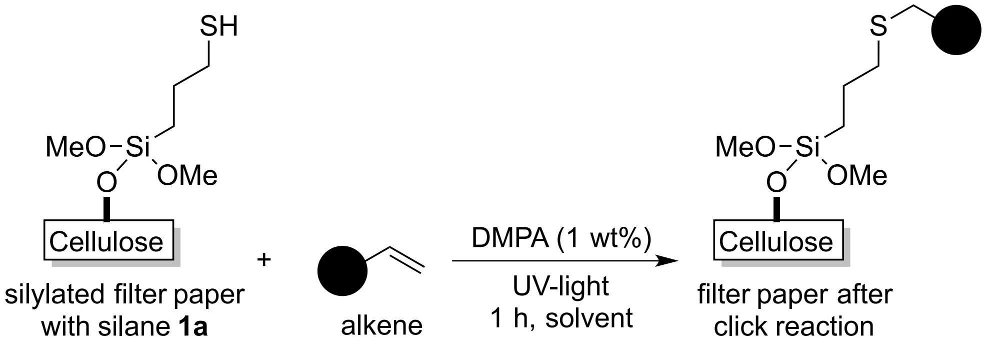

entry	alkene	solvent	angle
1	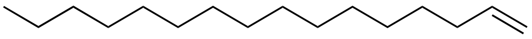	DMF	129[a]
2	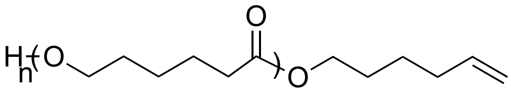	DMF	117[a]
3	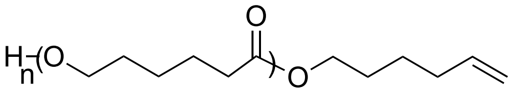	DMF	101[b]
4	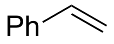	neat	117[a]
5	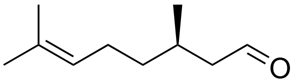	neat	111[a]
6	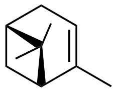	neat	111[a]

(a) Cellulose silylated with (*S*)-tartaric acid giving contact angle 115 prior to click reaction. (b) Cellulose silylated without acid giving contact angle 109 prior to click reaction.

Table S11. Click reaction of filter paper modified with silane **1b**

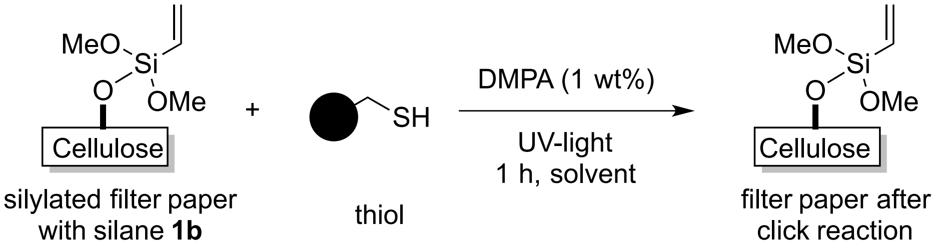

entry	thiol	solvent	angle
1	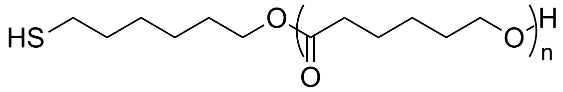	DMF	123[a]
2	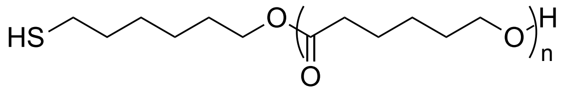	DMF	100[b]
3	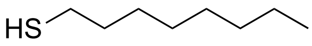	neat	123[b]
4	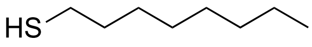	neat	116[b]

[a] Cellulose silylated with (*S*)-tartaric acid giving contact angle 126 prior to click reaction. [b] Cellulose silylated without acid giving contact angle 118 prior to click reaction.

Table S12. Click reaction of filter paper modified with silane **1c**

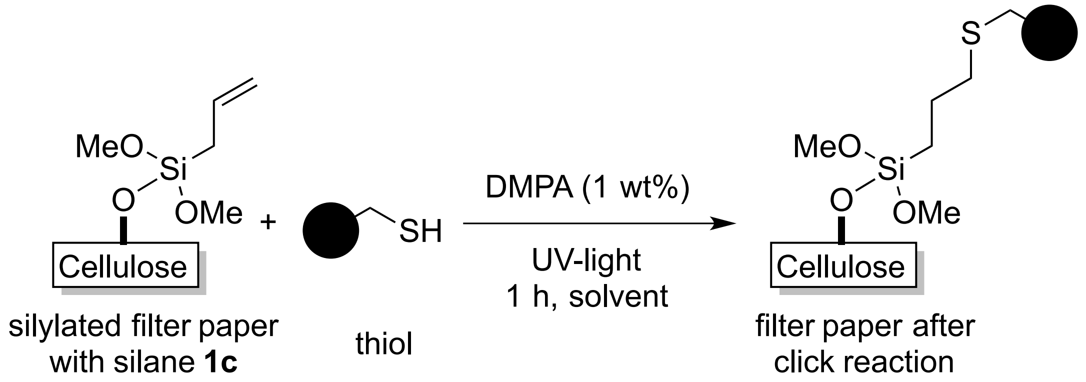

entry	thiol	solvent	angle
1	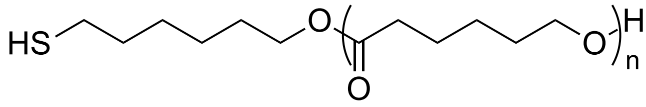	DMF	129[a]
2	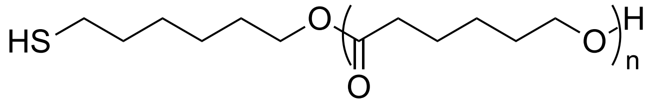	DMF	124[b]
3	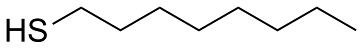	neat	127[b]

(a) Cellulose silylated with (*S*)-tartaric acid giving contact angle 125 prior to click reaction. (b) Cellulose silylated without acid giving contact angle 115 prior to click reaction.

Table S13. Elemental analysis of silylated filter paper

Silane	Catalyst	Element	C	H	O	Si	S	N
**1a**	no acid	% Found	42.28	6.04	50.21	0.15	<0.10	-
**1a**	acid	% Found	41.84	6.14	48.88	0.29	0.65	-
**1c**	acid	% Found	42.47	5.93	44.48	0.18	-	-
**1e**	acid	% Found	41.89	6.30	43.56	0.45	-	0.44

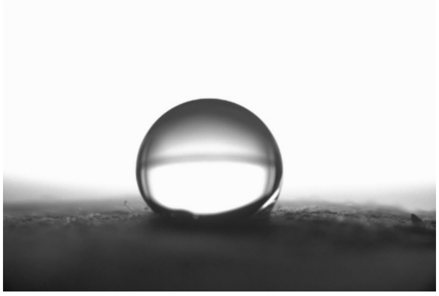

Figure S8. *A water droplet on a hydrophobized filter paper with C-16 hexadecene*

Procedure for the thiol-ene 'click' reaction between TPSi-1a-modified-filter paper and Quinidine or Quinine

To a scintillation vial (20 mL) containing TPSi-**1a**-filter paper (30-32 mg) and Quinidine or Quinine (30 mg, 0.1 mmol) in DMF (0.5 mL) was added DMPA (2 mol%). Next, the reaction was irradiated for 24h using an UV-lamp (UV-B bulb, TL20W/12, 20 W). The UV-treated filter paper was removed and next extracted using a Soxhlet extractor and acetone as the solvent for 17h. The extracted was filter paper was subsequently dried overnight under reduced pressure. The cinchona-alkaloid-modified NFC exhibit high UV-activity and was fluorescent (Figure S9 and S10).

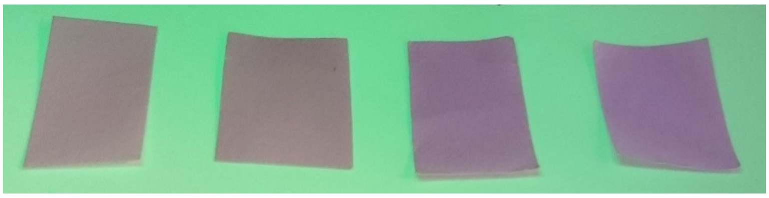

Figure S9. *UV-activity of modified filter paper (short wave):*

From left: Unmodified filter paper, modified filter paper with (3-mercaptopropyl)trimethoxysilane, filter paper after click reaction with Quinidine, filter paper after click reaction with Quinine.

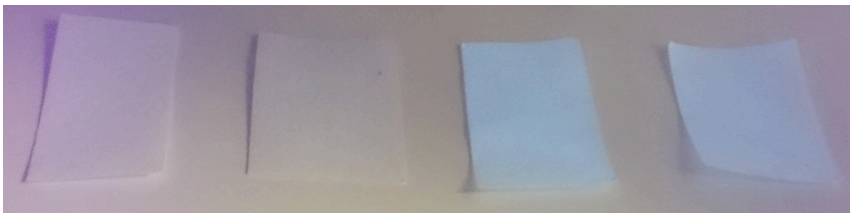

Figure S10. *UV-activity of modified filter paper (long wave):*

From left: Unmodified filter paper, modified filter paper with (3-mercaptopropyl)trimethoxysilane, filter paper after click reaction with Quinidine, filter paper after click reaction with Quinine.

References

1. Li, D.; Sevastyanova, O.; Ek. M. Pretreatment of softwood dissolving pulp with ionic liquids. Holzforschung, **66**, 935-943 (2012).
2. Saito, T.; Nishiyama, Y.; Putaux, J. L.; Vignon, M.; Isogai, A. Homogeneous suspensions of individualized microfibrils from TEMPO-catalyzed oxidation of native cellulose. *Biomacromolecules*, **7**, 1687–1691 (2006).
3. Rahimi, A.; Azarpira, A.; Kim, H.; Ralph, J.; Stahl, S. S. Chemoselective Metal-Free Aerobic Alcohol Oxidation in Lignin. *J. Am. Chem. Soc.* **135**, 6415–6418 (2013).
4. Wang, X.; Liu, R.; Jin, Y.; Liang, X. TEMPO/HCl/$NaNO_2$ Catalyst: A Transition-Metal-Free Approach to Efficient Aerobic Oxidation of Alcohols to Aldehydes and Ketones Under Mild Conditions. *Chem. Eur. J.* **14**, 2679–2685 (2008).
5. Hedfors, C. et al. Thiol end-functionalization of poly(e-caprolactone), catalysed by *Candida antarctica* lipase B. *Macromolecules* **38**, 647-649 (2005).
6. Persson, V. P. et. al. Direct organocatalytic chemoselective synthesis of a dendrimer-like star polyester. *Macromolecules* **39**, 2819-2822 (2006).
7. Premi, C.; Jain, N. Phosphane-Free Hiyama Cross-coupling of Aryl and Heteroaryl Halides Catalyzed by Palladium Nanoparticles in Ionic Liquids. *Eur. J. Org. Chem.* 5493–5499 (2013).
8. Tucker-Schwartz, A. K. et al. Thiol-ene click recation as a general route to functional trialkoxysilanes for surface coating applications. *J. Am. Chem. Soc.* **133**, 11026-11029 (2011).

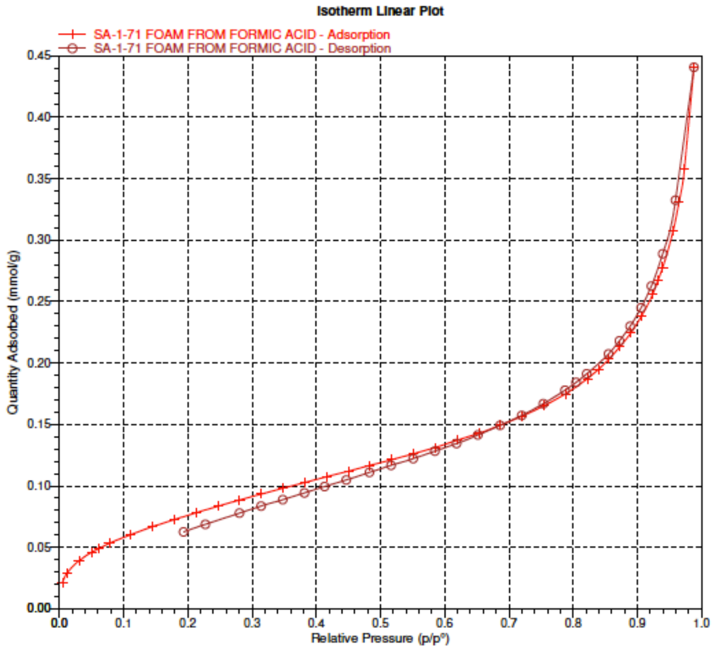

Figure S11. *N_2 sorption isotherms of formic acid fabricated NFC foam material*

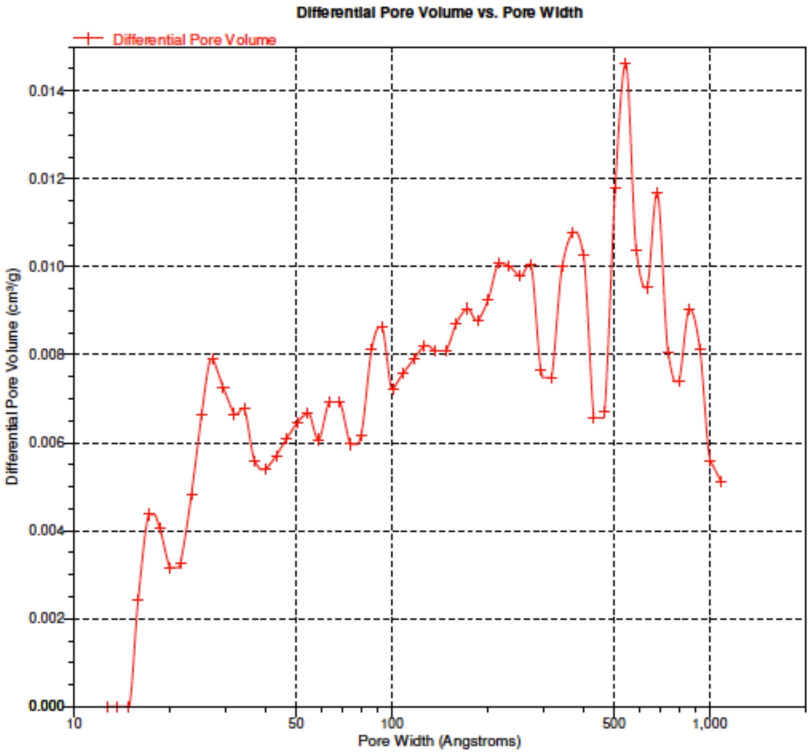

Figure S12. *Pore size distribution of formic acid fabricated NFC foam material using the adsorption isotherms with density functional theory*

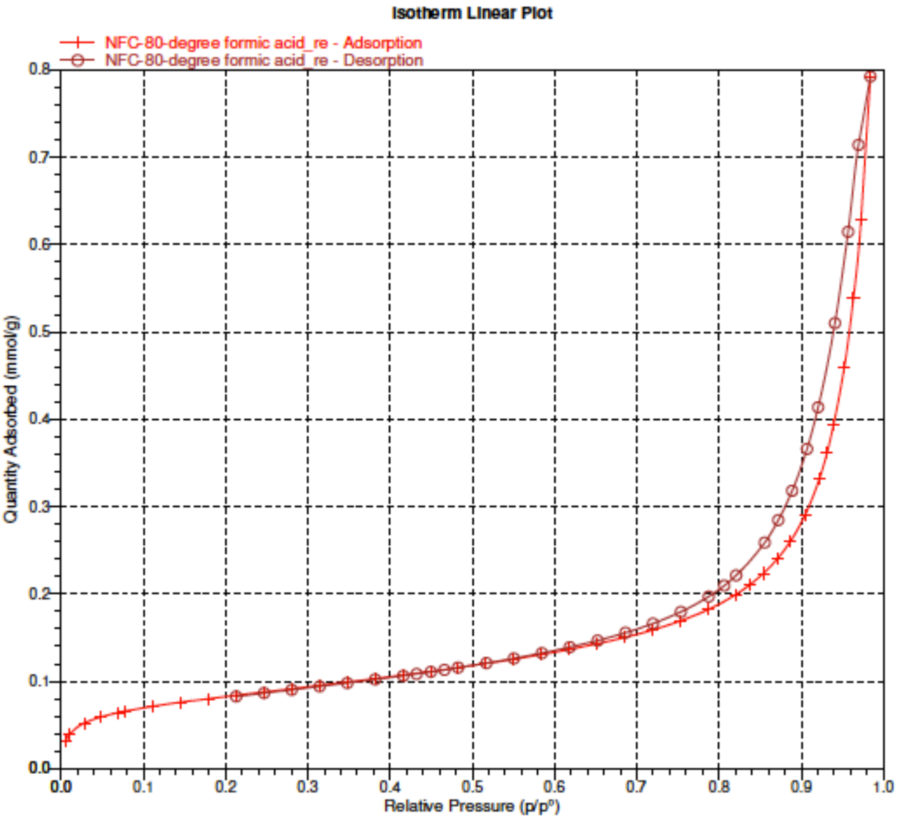

Figure 13. *N_2 sorption isotherms of formic acid fabricated NFC foam material performed at 80 ^{o}C*

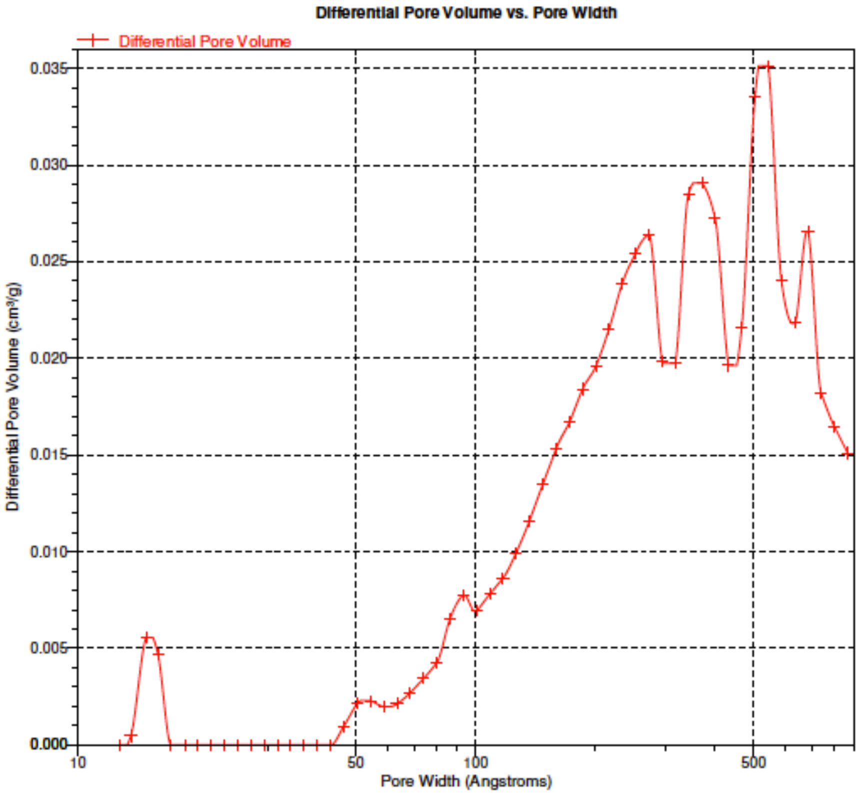

Figure S14. *Pore size distribution of formic acid fabricated NFC foam material performed at 80 ^{o}C using the adsorption isotherms with density functional theory*

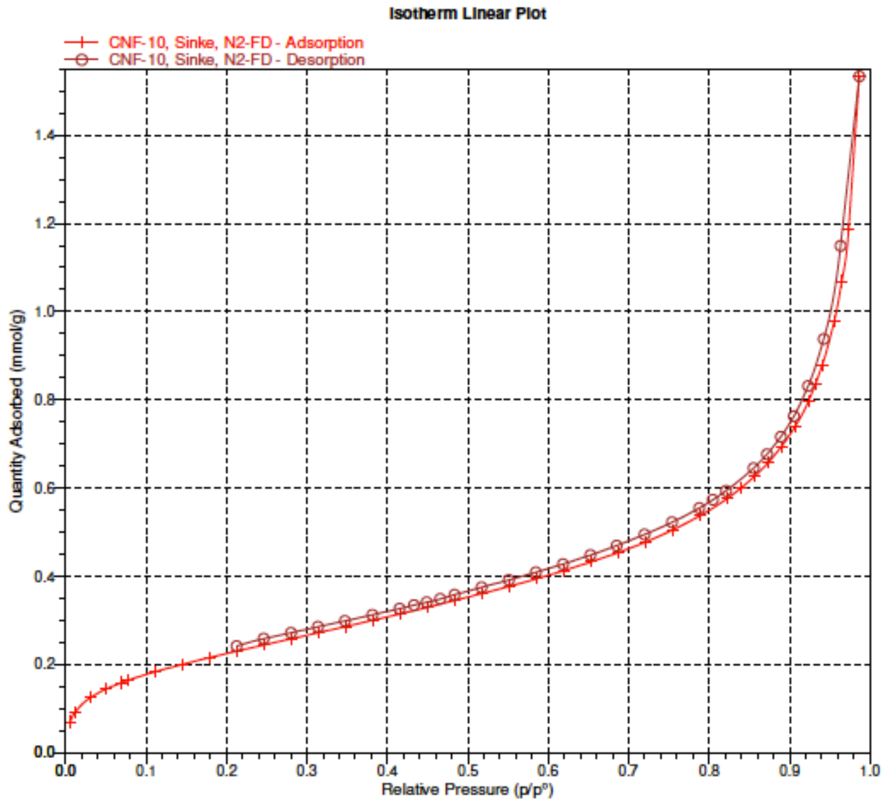

Figure S15. *N_2 sorption isotherms of formic acid fabricated NFC foam material*

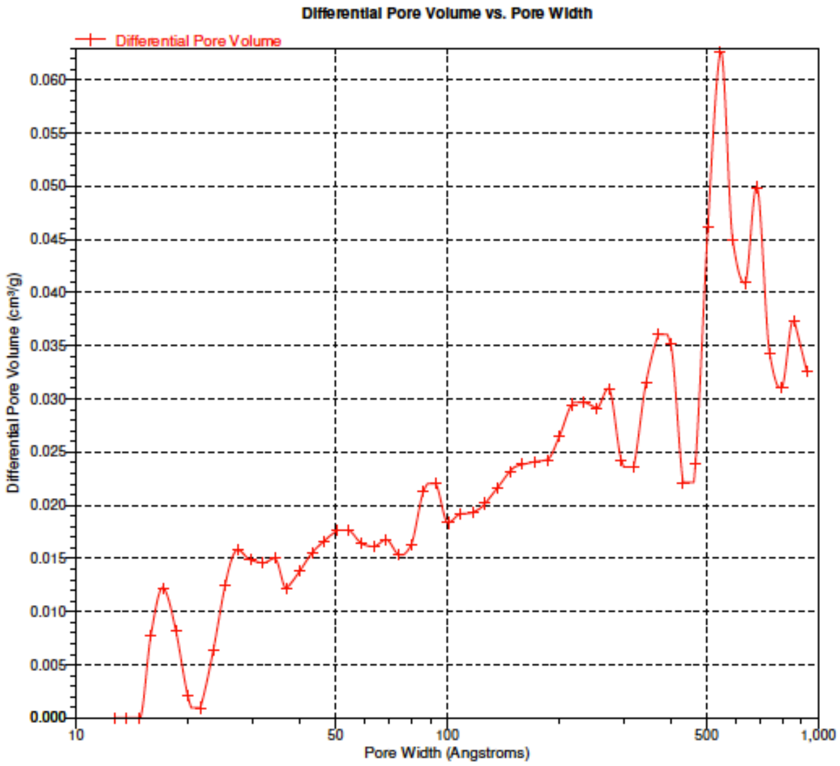

Figure S16. *Pore size distribution of formic acid fabricated NFC foam material using the adsorption isotherms with density functional theory*

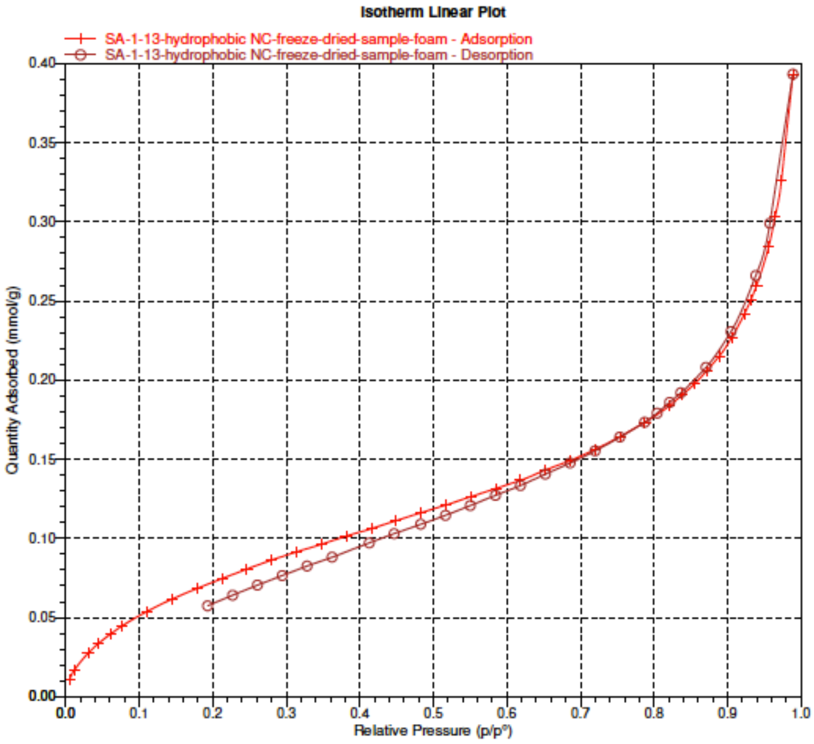

Figure S17. *N_2 sorption isotherms of hydrophobized formic acid fabricated NFC foam material with C-16 silane **1d***

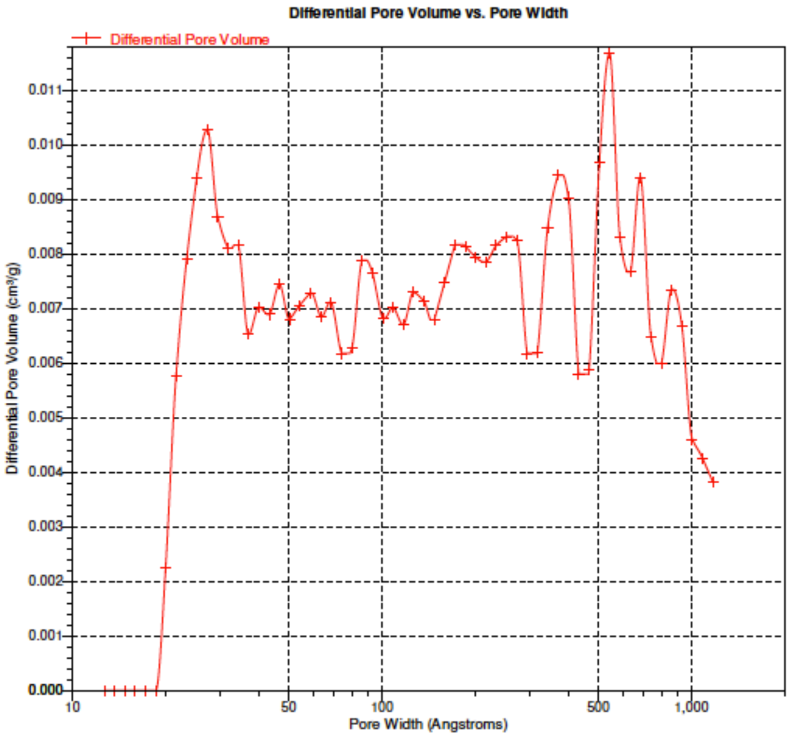

Figure S18. *Pore size distribution of hydrophobized formic acid fabricated NFC foam material with C-16 **1d** using the adsorption isotherms with density functional theory*

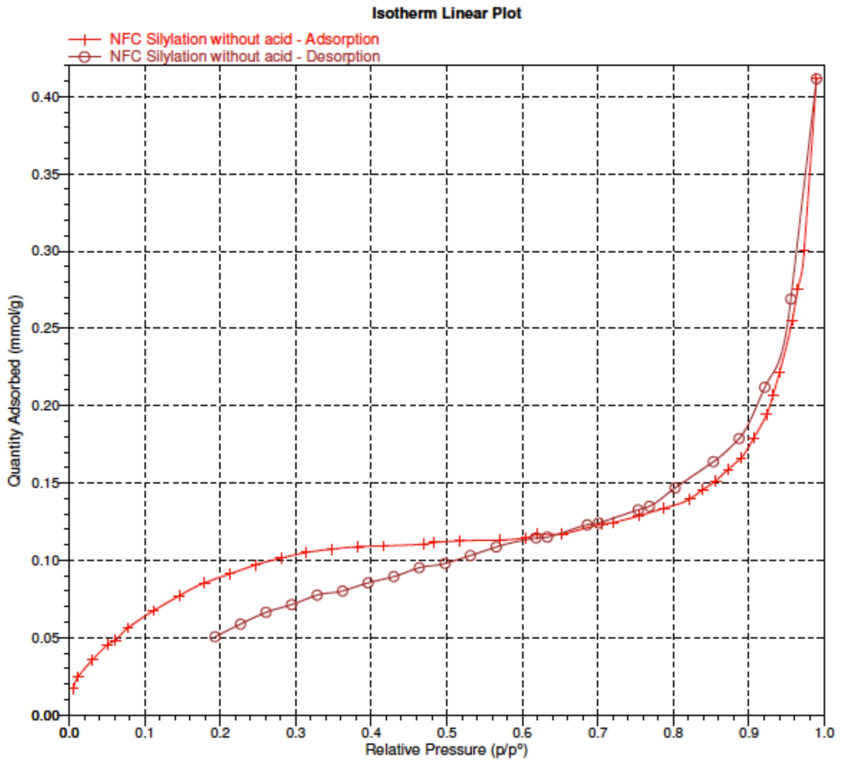

Figure S19. *N_2 sorption isotherms of formic acid fabricated NFC silylated without acid with silane* ***1a***

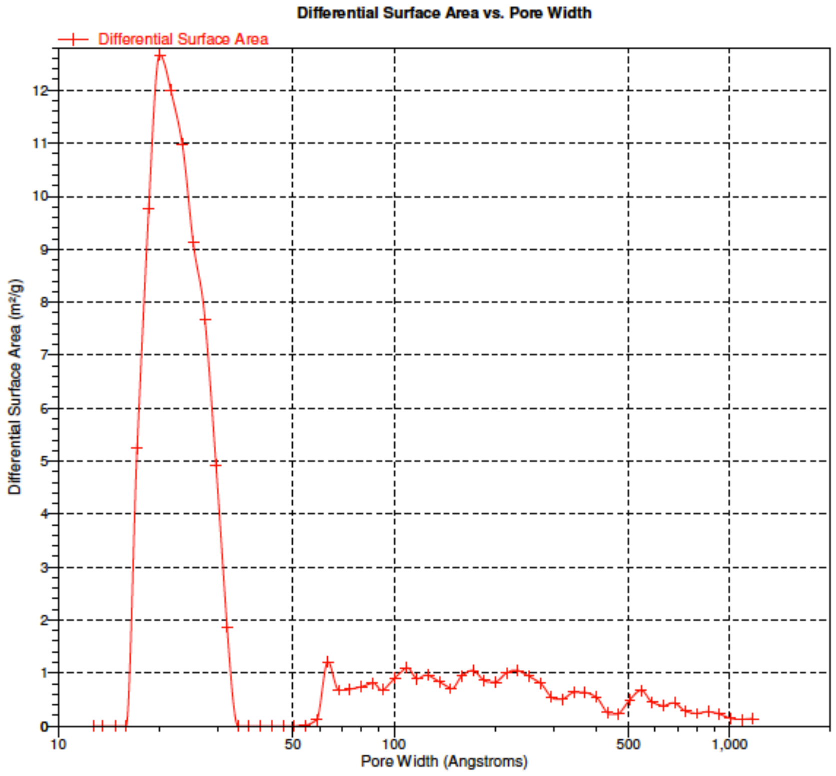

Figure S20. *Pore size distribution of formic acid fabricated NFC silylated without acid with silane* ***1a***

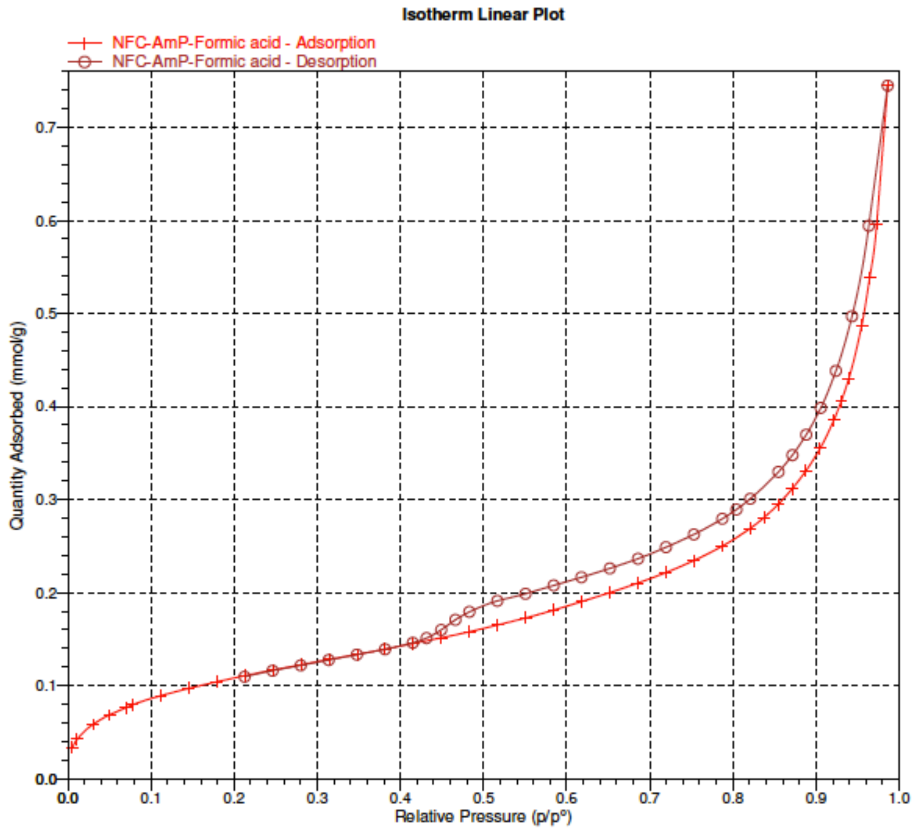

Figure S21. *N_2 sorption isotherms of AmP-NFC from formic acid fabricated NFC*

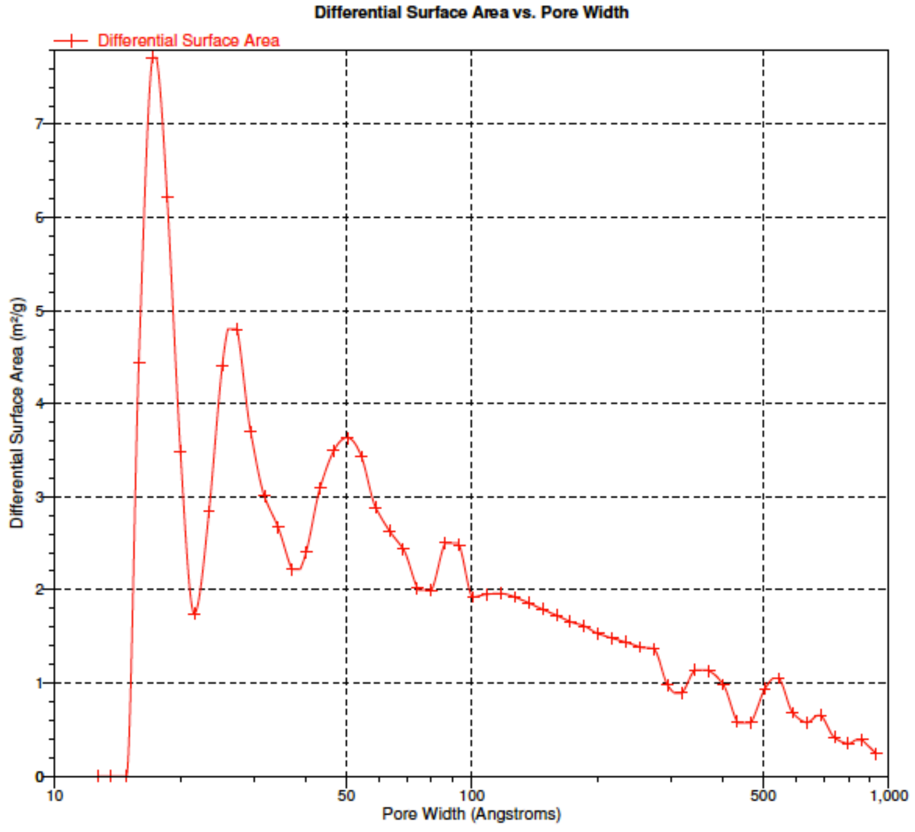

Figure S22. *Pore size distribution of AmP-NFC from formic acid fabricated NFC*

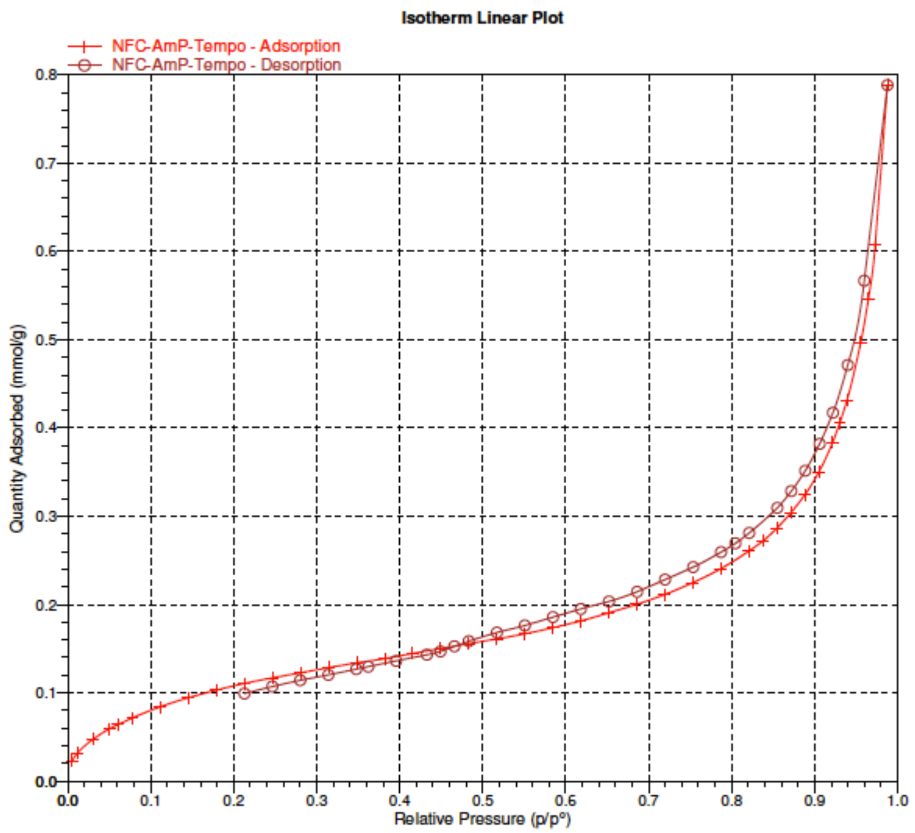

Figure S23. *N_2 sorption isotherms of AmP-NFC from NFC derived from TEMPO-NaClO oxidation-homogenization route*

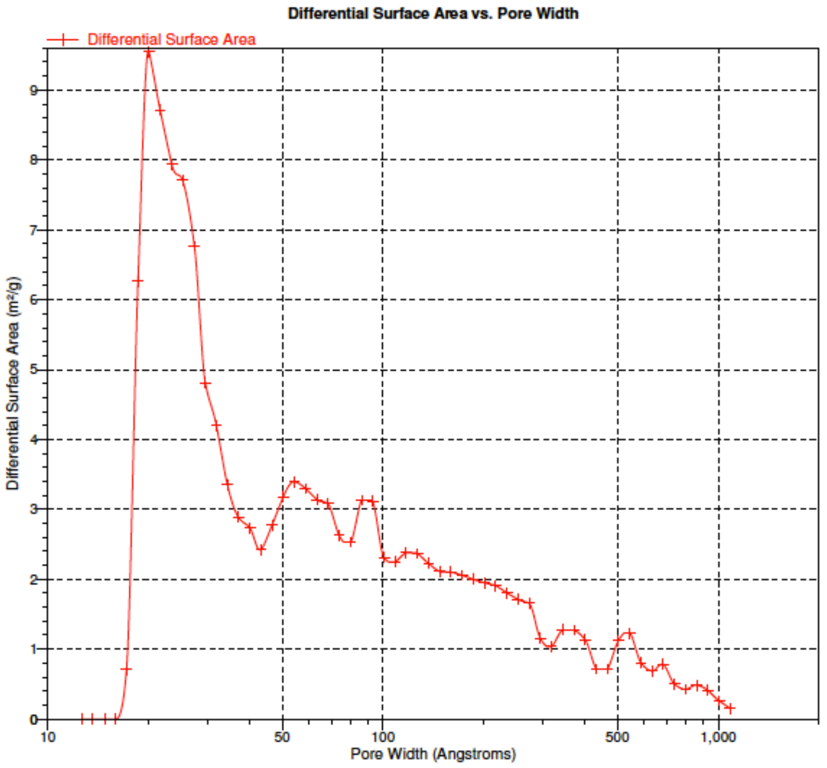

Figure S24. *Pore size distribution of NFC-AmP from NFC derived from TEMPO-NaClO oxidation-homogenization route*

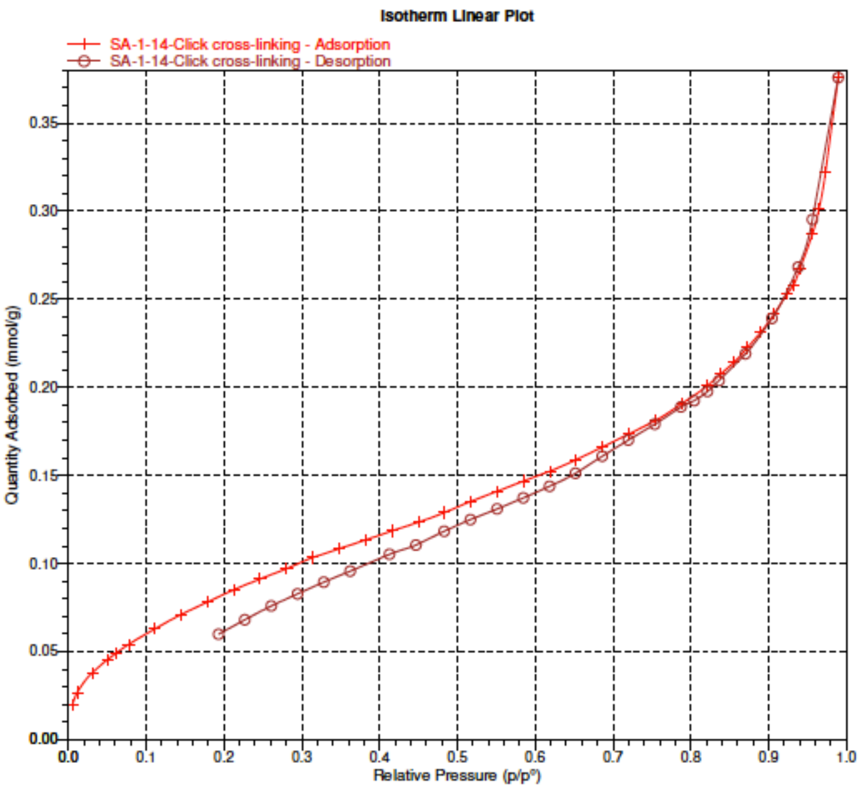

Figure S25. *N_2 sorption isotherms of NFC with equal amounts of **1a** and **1c** and it subsequent UV-treatment under "click" conditions. Formic acid fabricated NFC was used*

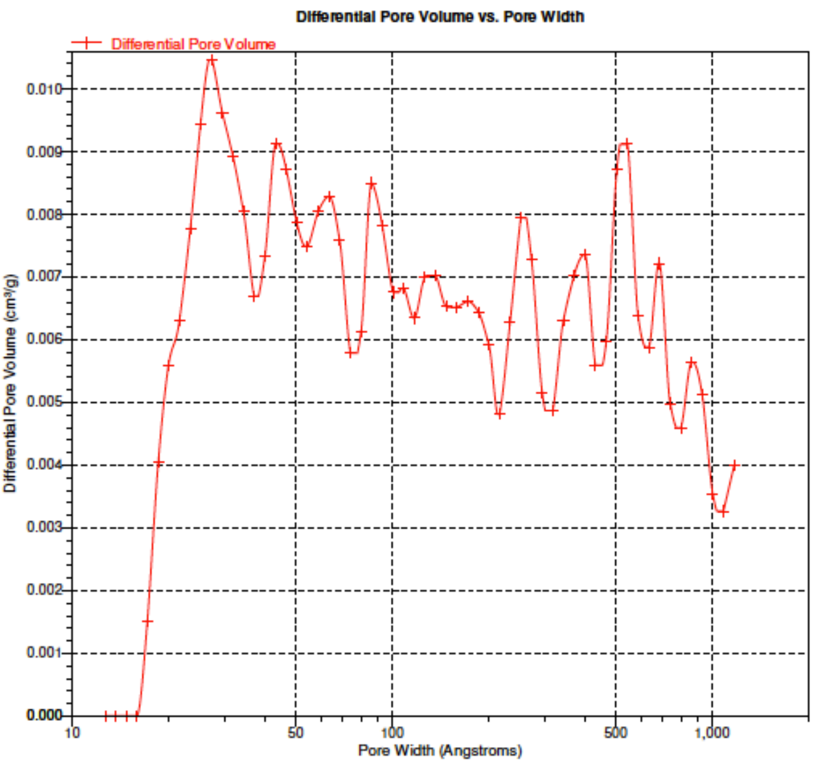

Figure S26. *Pore size distribution of NFC with equal amounts of **1a** and **1c** and it subsequent UV-treatment under "click" conditions using the adsorption isotherms with density functional theory. Formic acid fabricated NFC was used*

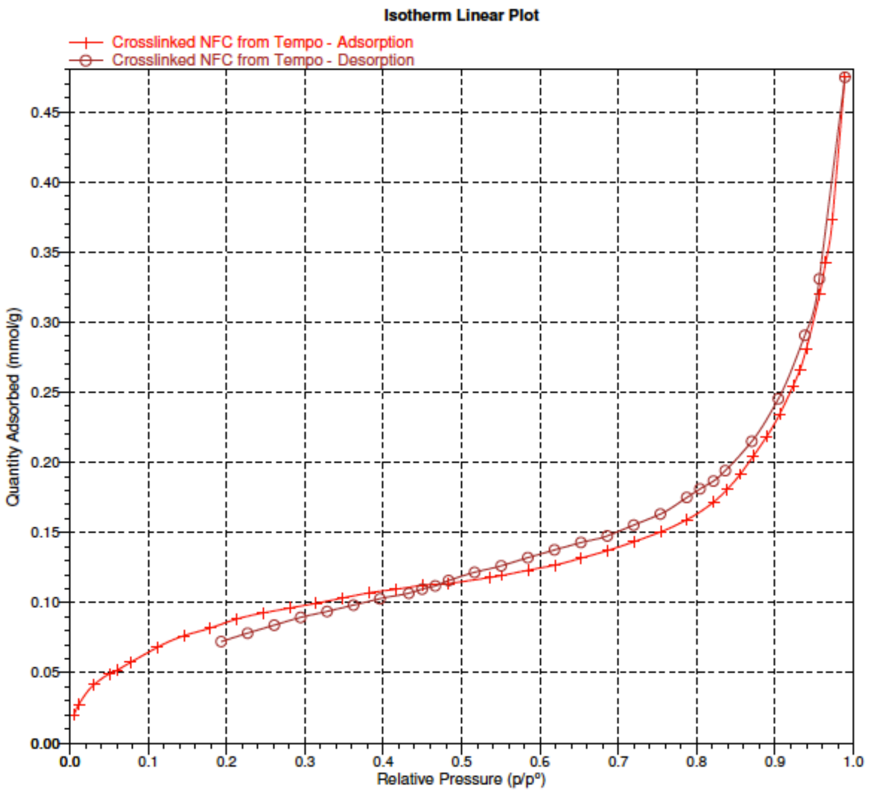

Figure S27. *N_2 sorption isotherms of NFC with equal amounts of **1a** and **1c** and it subsequent UV-treatment under "click" conditions. NFC derived from TEMPO-NaClO oxidation-homogenization route was used*

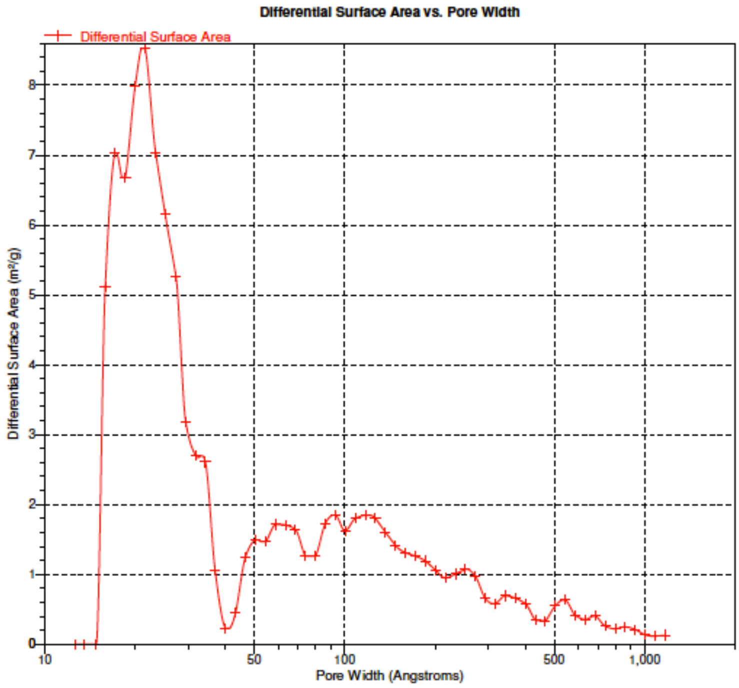

Figure S28. *Pore size distribution of NFC with equal amounts of **1a** and **1c** and it subsequent UV-treatment under "click" conditions using the adsorption isotherms with density functional theory. NFC derived from TEMPO-NaClO oxidation-homogenization route was used*

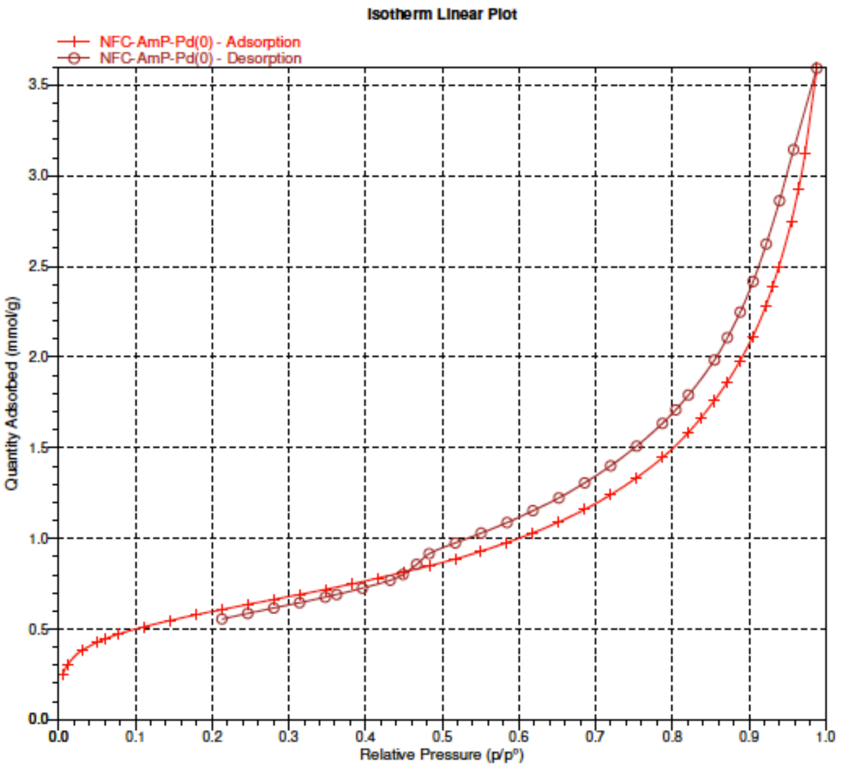

Figure S29. *N_2 sorption isotherms of AmP-NFC-Pd(0). Formic acid fabricated NFC was used*

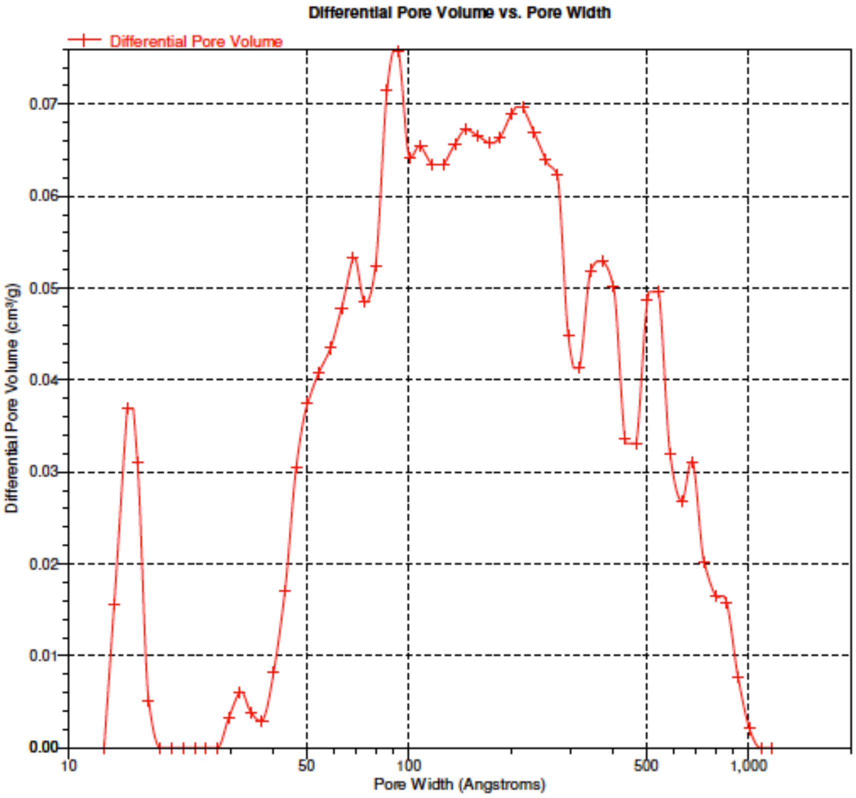

Figure S30. *Pore size distribution of NFC-AmP-Pd(0) using the adsorption isotherms with density functional theory. Formic acid fabricated NFC was used*

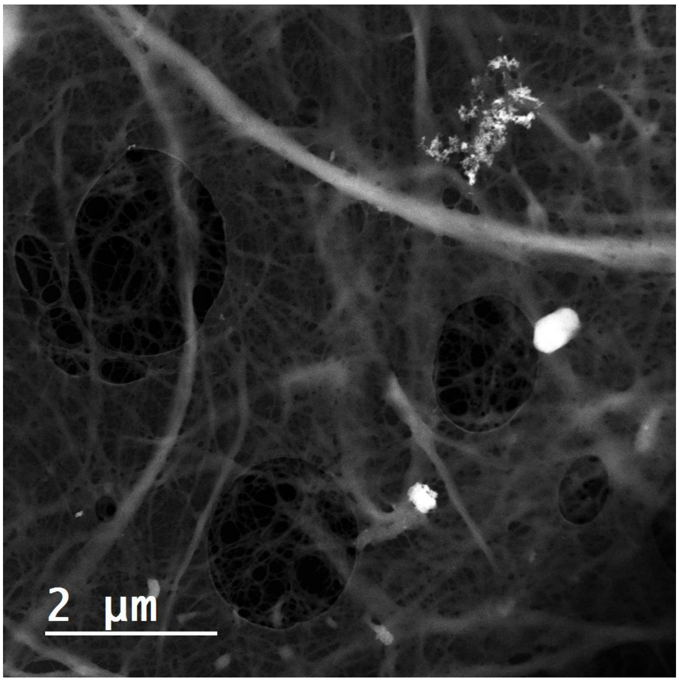

Figure S31. *High-angle annular dark-field scanning transmission electron micrograph (HAADF-STEM) image of sulphite-softwood-dissolving pulp*

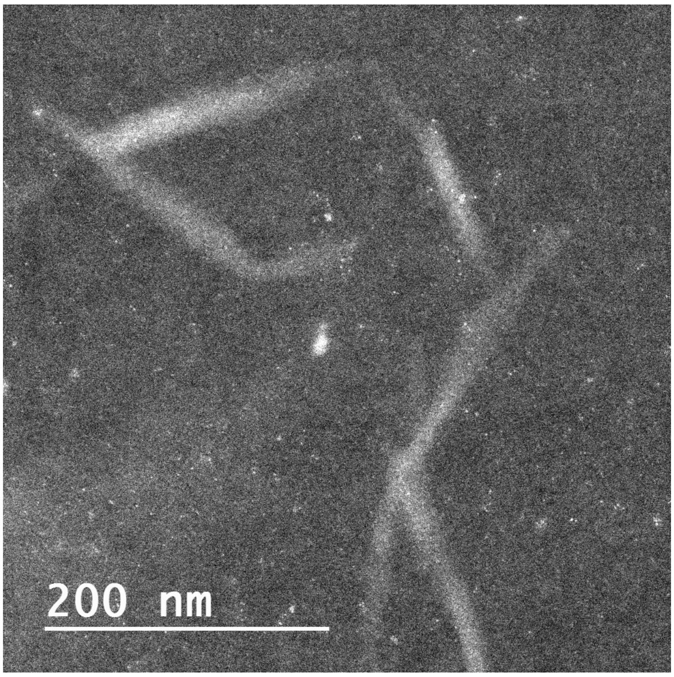

Figure S32. *High-angle annular dark-field scanning transmission electron micrograph (HAADF-STEM) image of formic acid fabricated NFC foam material*

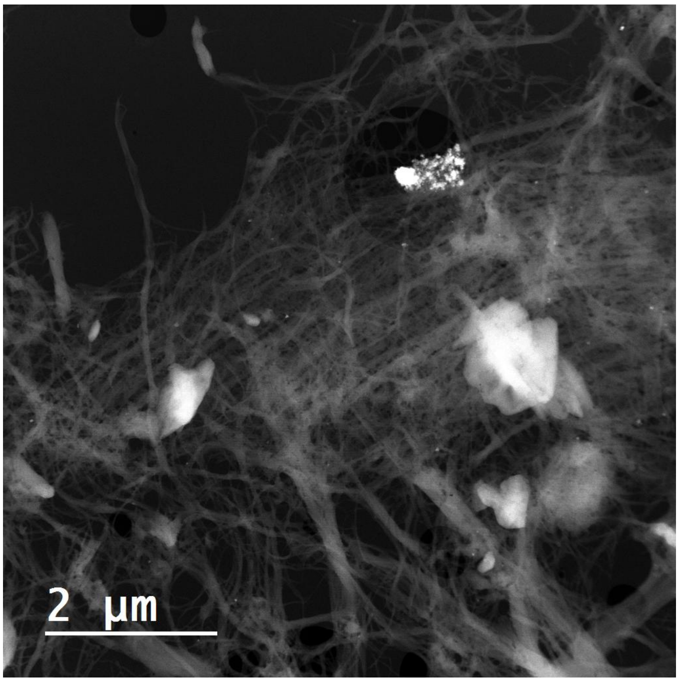

Figure S33. *High-angle annular dark-field scanning transmission electron micrograph (HAADF-STEM) image of formic acid fabricated NFC with 50% formic acid*

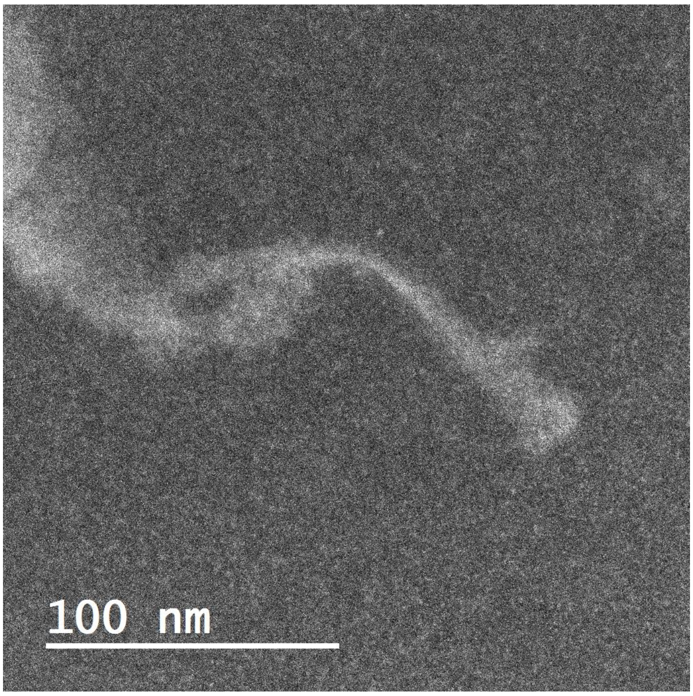

Figure S34. *High-angle annular dark-field scanning transmission electron micrograph (HAADF-STEM) image of formic acid fabricated NFC at 80 °C*

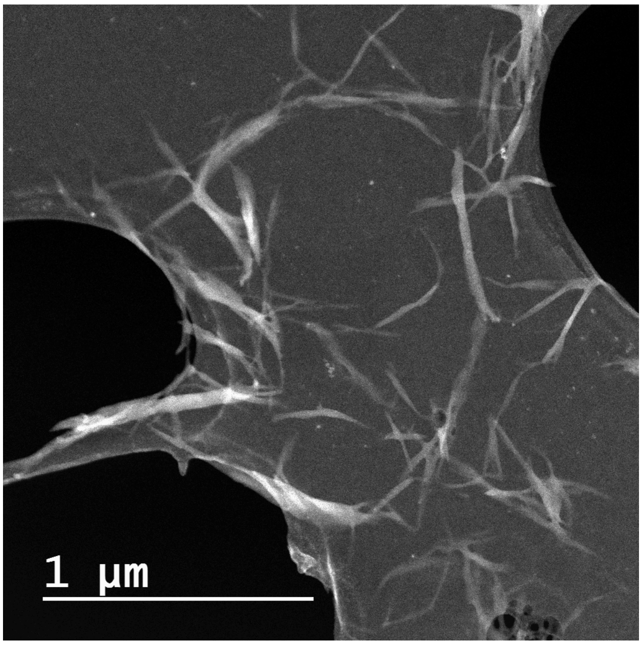

Figure S35. *High-angle annular dark-field scanning transmission electron micrograph (HAADF-STEM) image of formic acid fabricated with 180 minutes homogenization time*

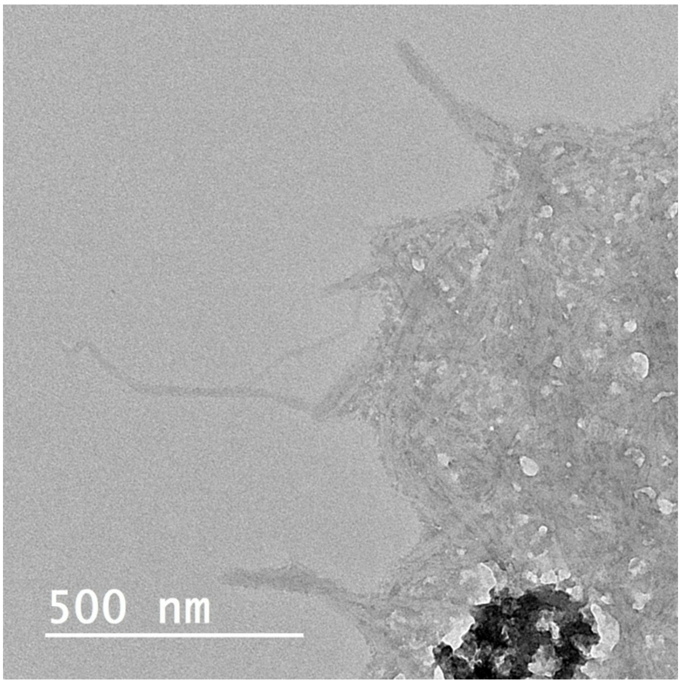

Figure S36. *TEM image of formic acid fabricated NFC with 180 minutes homogenization time and 48h reaction time*

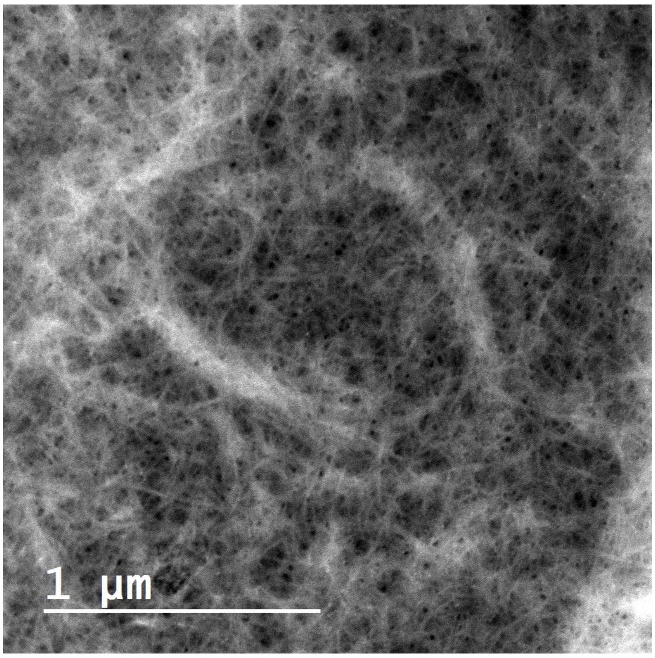

Figure S37. *High-angle annular dark-field scanning transmission electron micrograph (HAADF-STEM) image of NFC derived from TEMPO-NaClO oxidation-homogenization route*

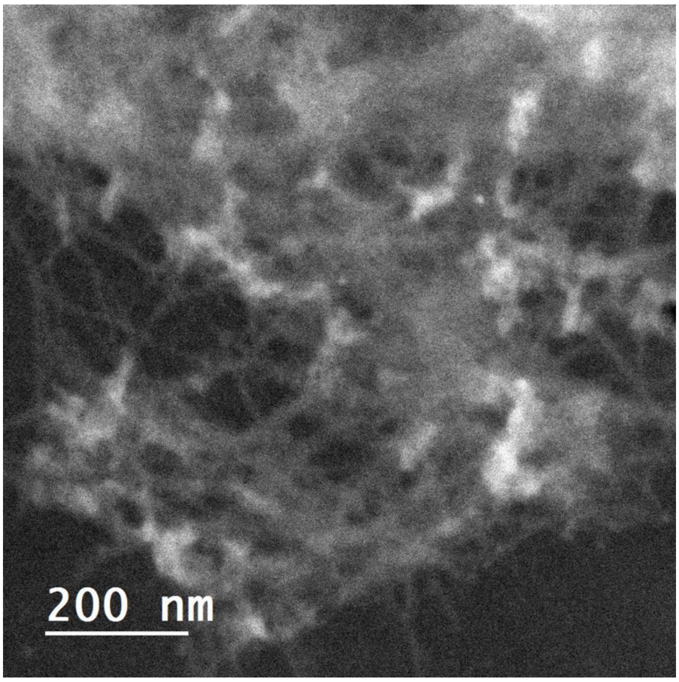

Figure S38. *High-angle annular dark-field scanning transmission electron micrograph (HAADF-STEM) image of NFC with equal amount of* ***1a*** *and* ***1c*** *and it subsequent UV-treatment under "click" conditons. NFC derived from TEMPO-NaClO oxidation-homogenization route was used*

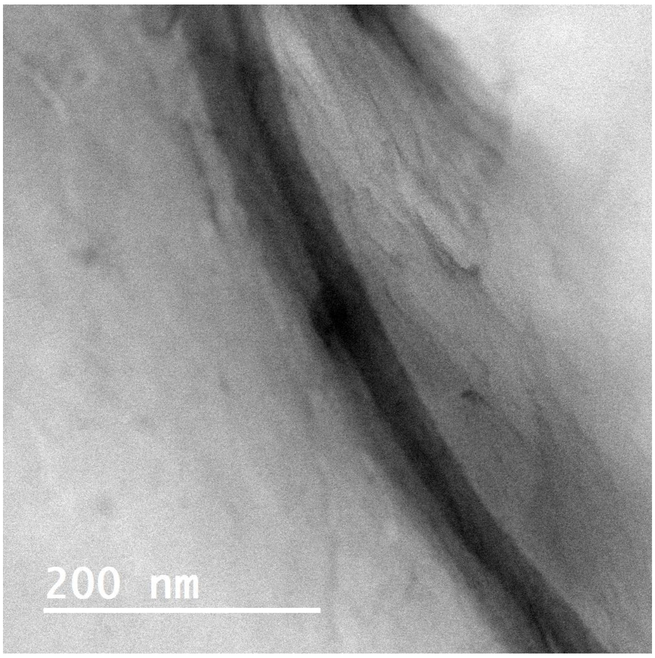

Figure S39. *STEM image of NFC with equal amount of **1a** and **1c** and it subsequent UV-treatment under "click" conditions. Formic acid fabricated NFC was used*

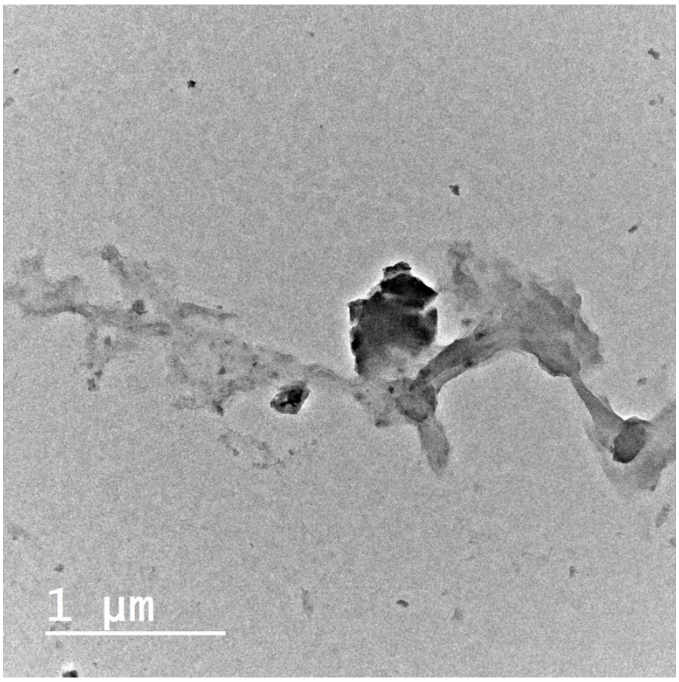

Figure S40. *TEM image of hydrophobized formic acid fabricated NFC with C-16 silane **1d***

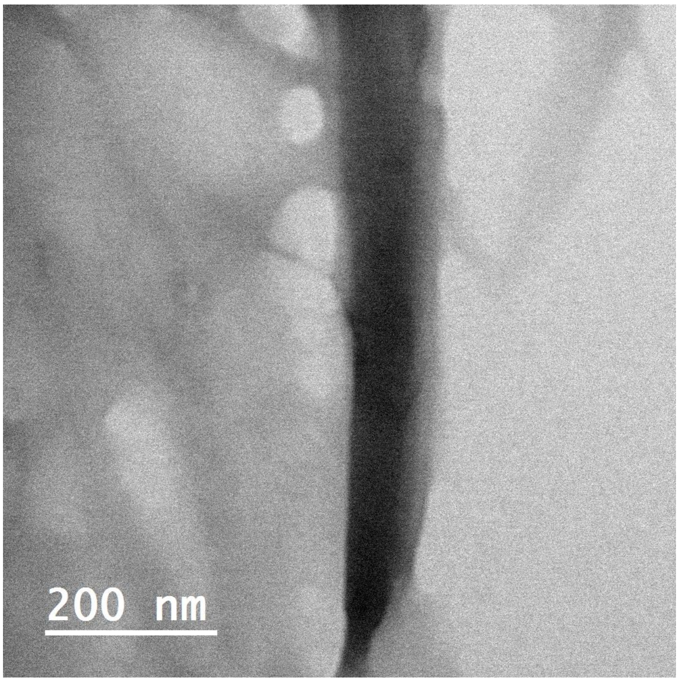

Figure S41. *High-angle annular dark-field scanning transmission electron micrograph (HAADF-STEM) image of hydrophobized NFC with C-16 silane* ***1d****. NFC derived from TEMPO-NaClO oxidation-homogenization route was used*

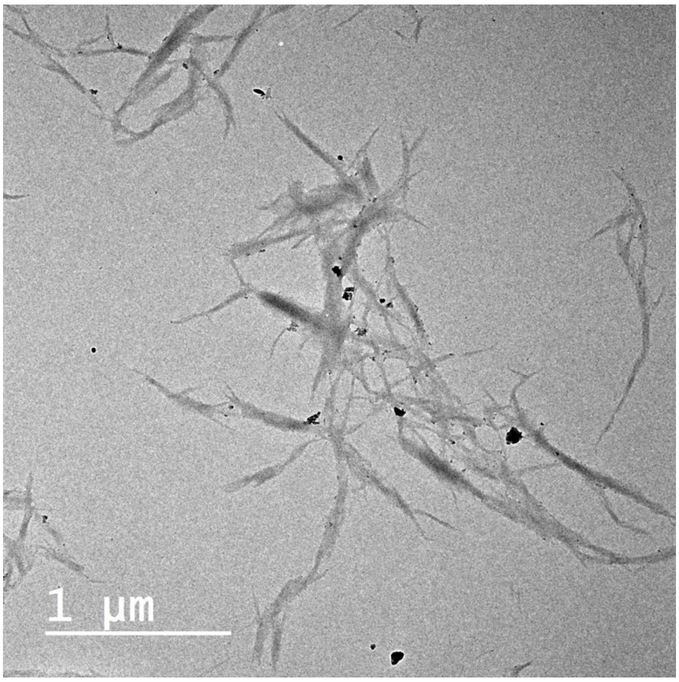

Figure S42. *TEM image of formic acid and ammonium formiate fabricated NFC*

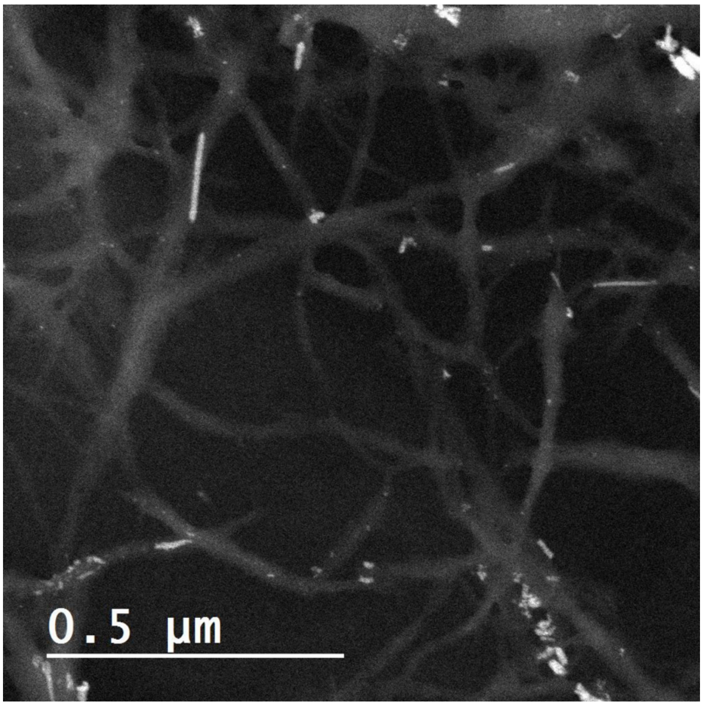

Figure S43. *High-angle annular dark-field scanning transmission electron micrograph (HAADF-STEM) image of tandem catalytic aerobic oxidation/formic acid fabricated NFC*

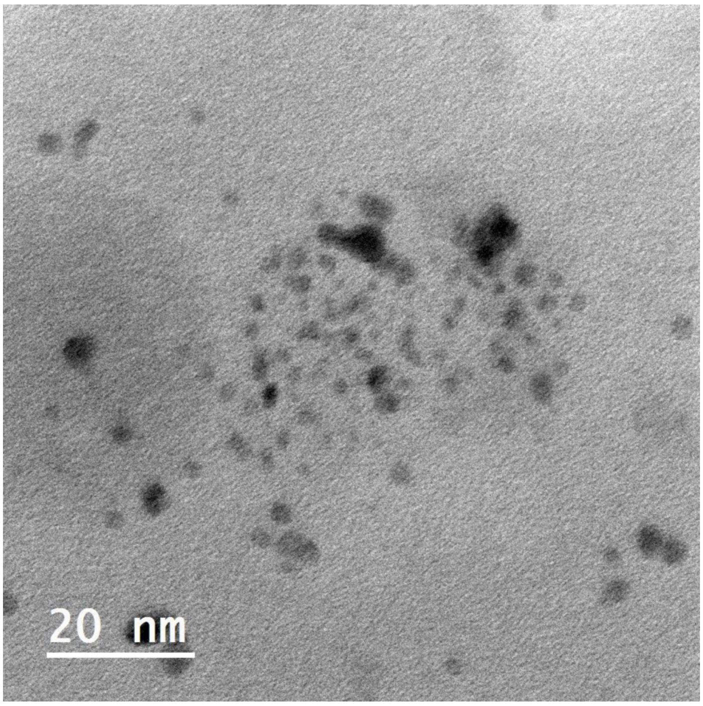

Figure S44. *Transmission electron micrograph bright field (STEM-BF) image* AmP-NFC-Pd(0).

The images of the Pd nanocatalyst indicated particle-size distribution of 1–5 nm

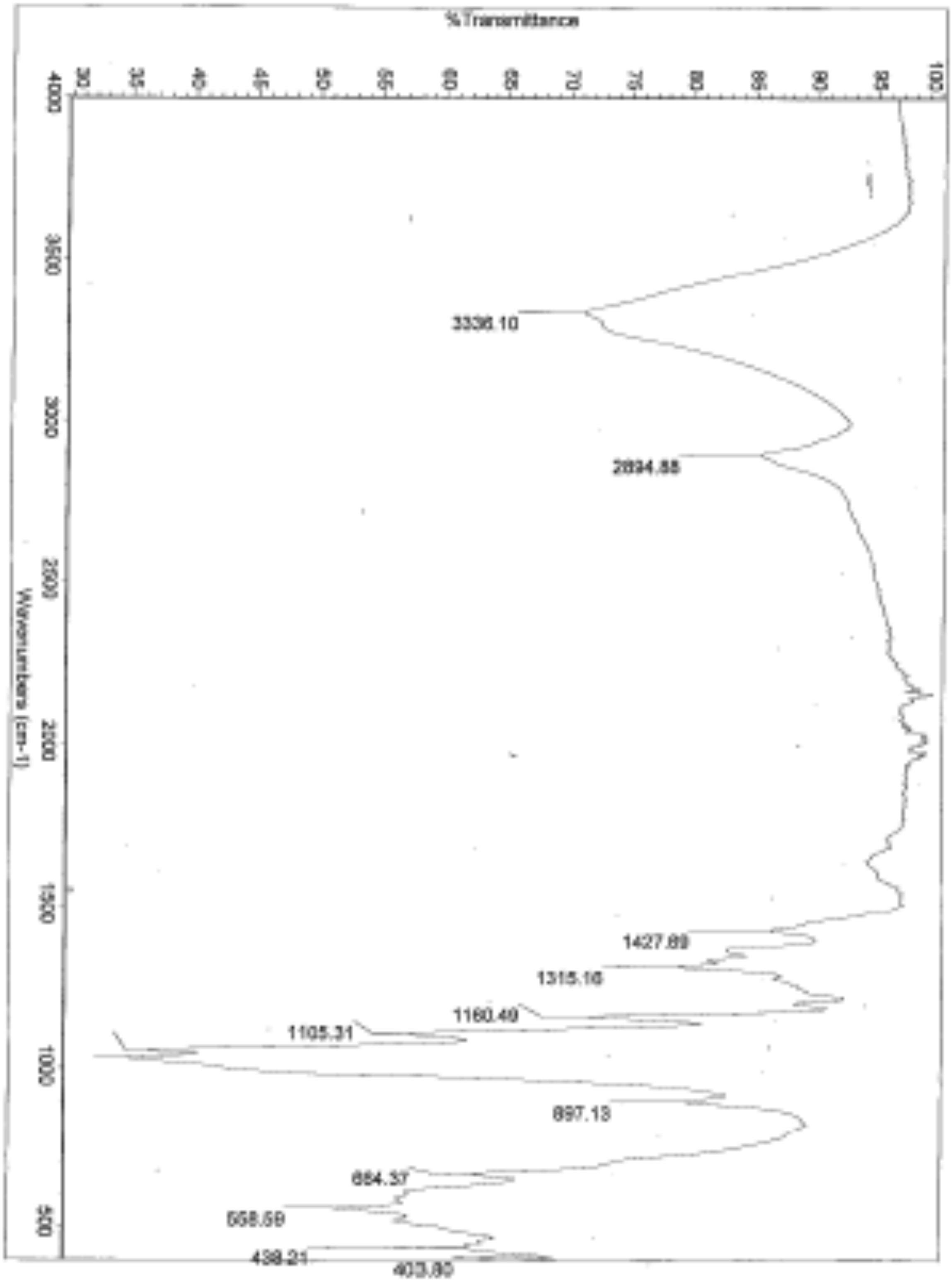

Figure S45. *FT-IR of formic acid fabricated NFC*

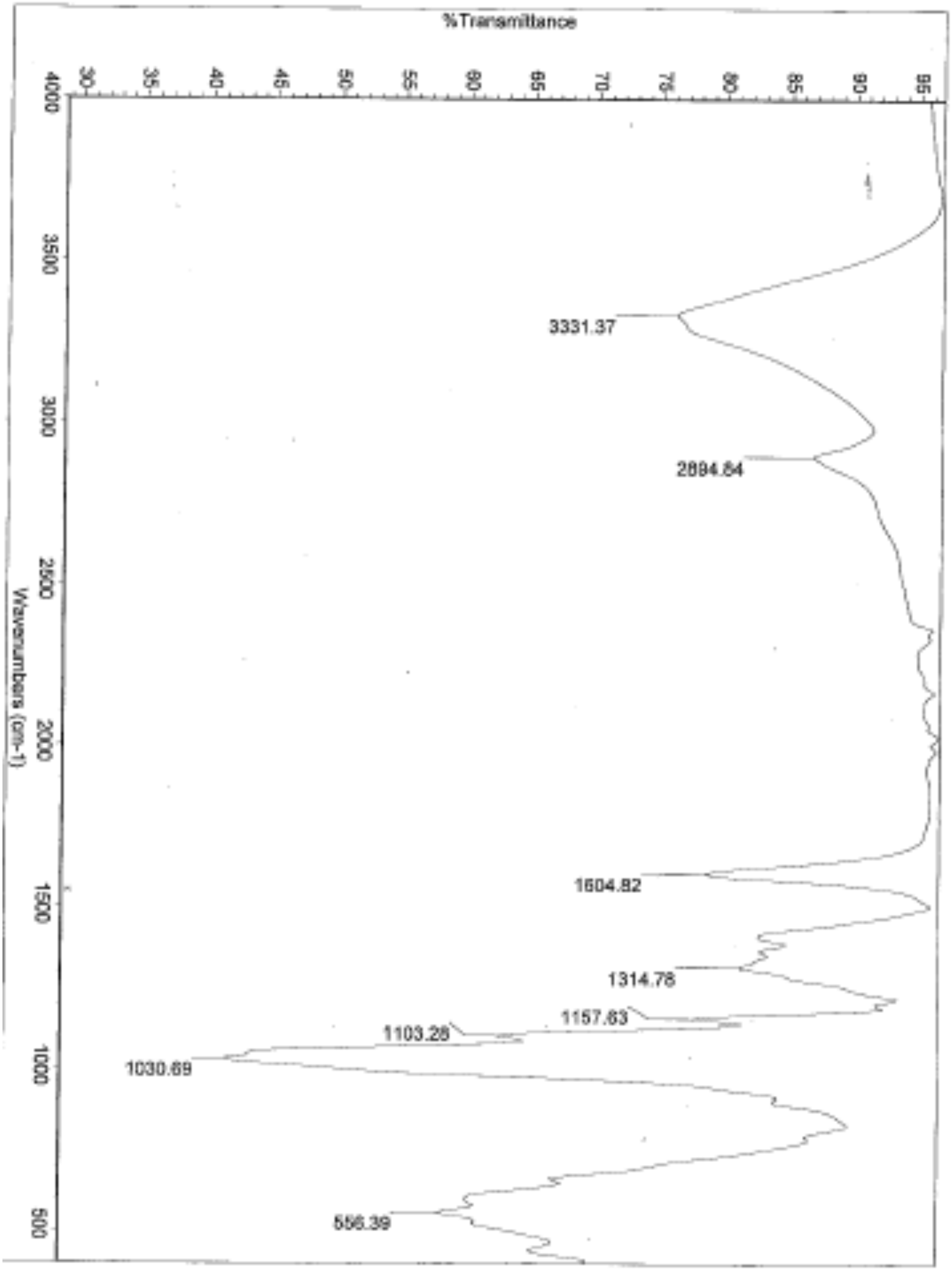

Figure S46. *FT-IR of NFC derived from TEMPO-NaClO oxidation-homogenization route*

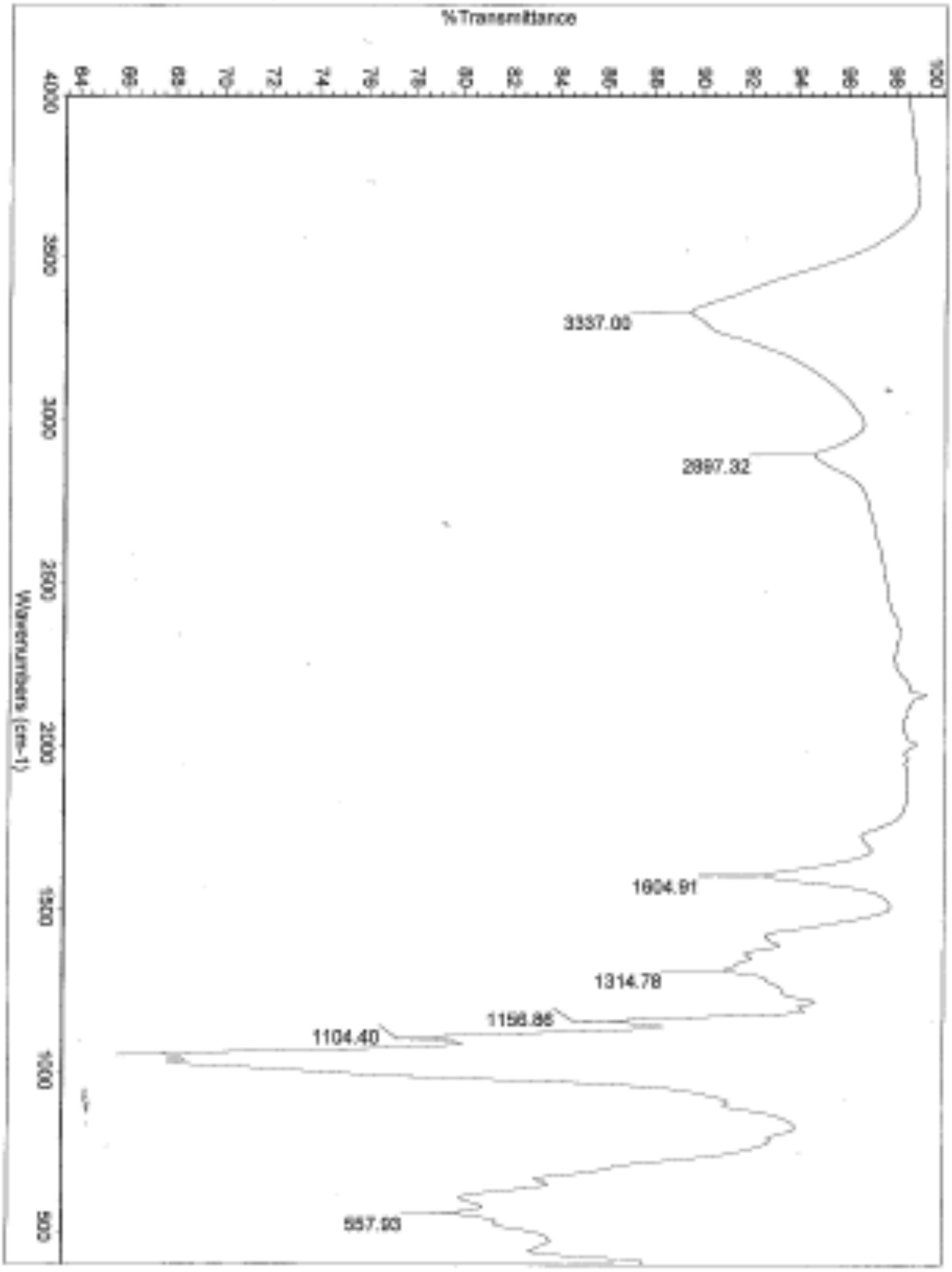

Figure S47. *FT-IR of NFC derived from TEMPO-NaClO oxidation-homogenization route silylated with* ***1a***

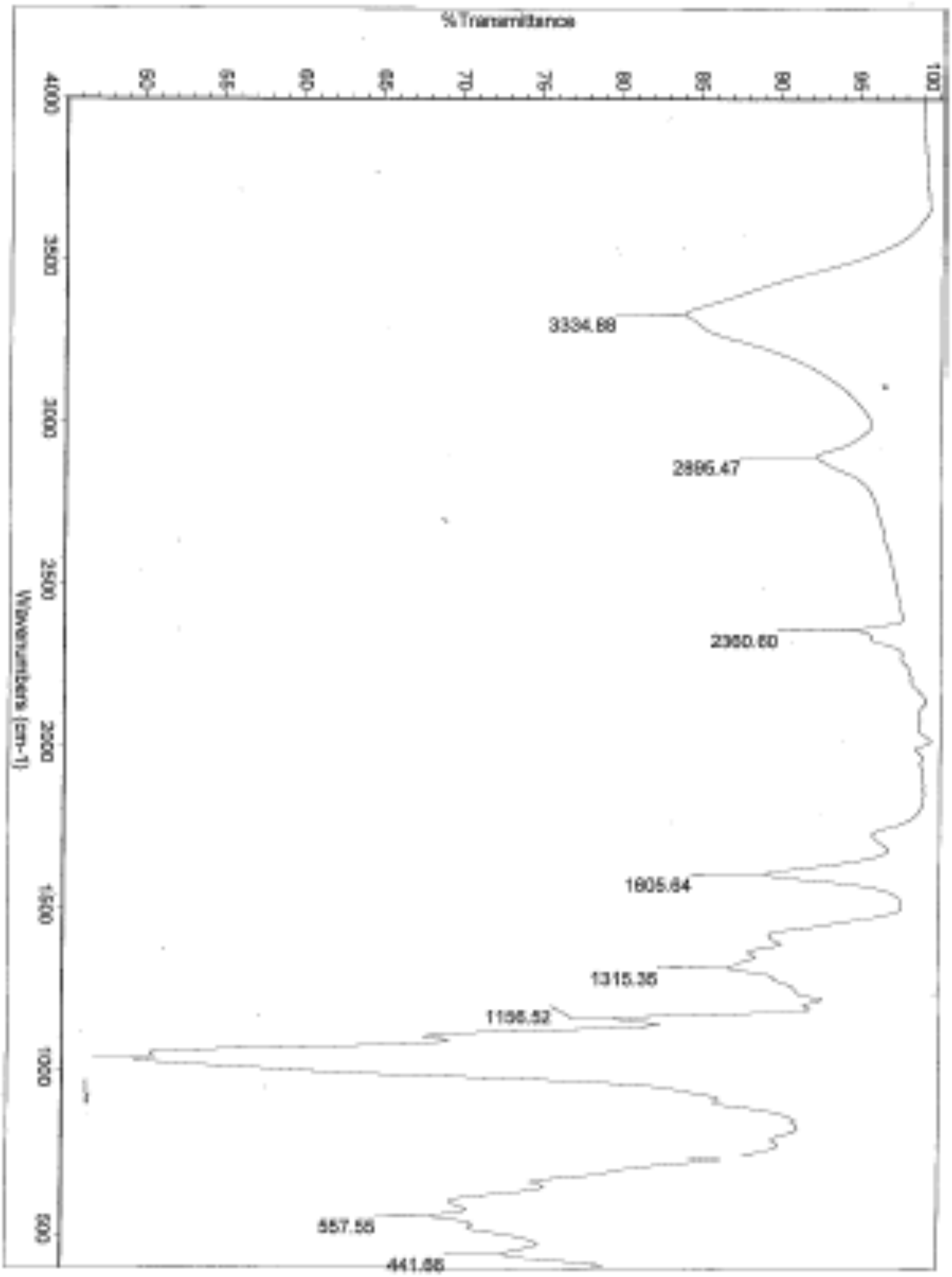

Figure S48. *FT-IR of NFC derived from TEMPO-NaClO oxidation-homogenization route silylated with* ***1c***

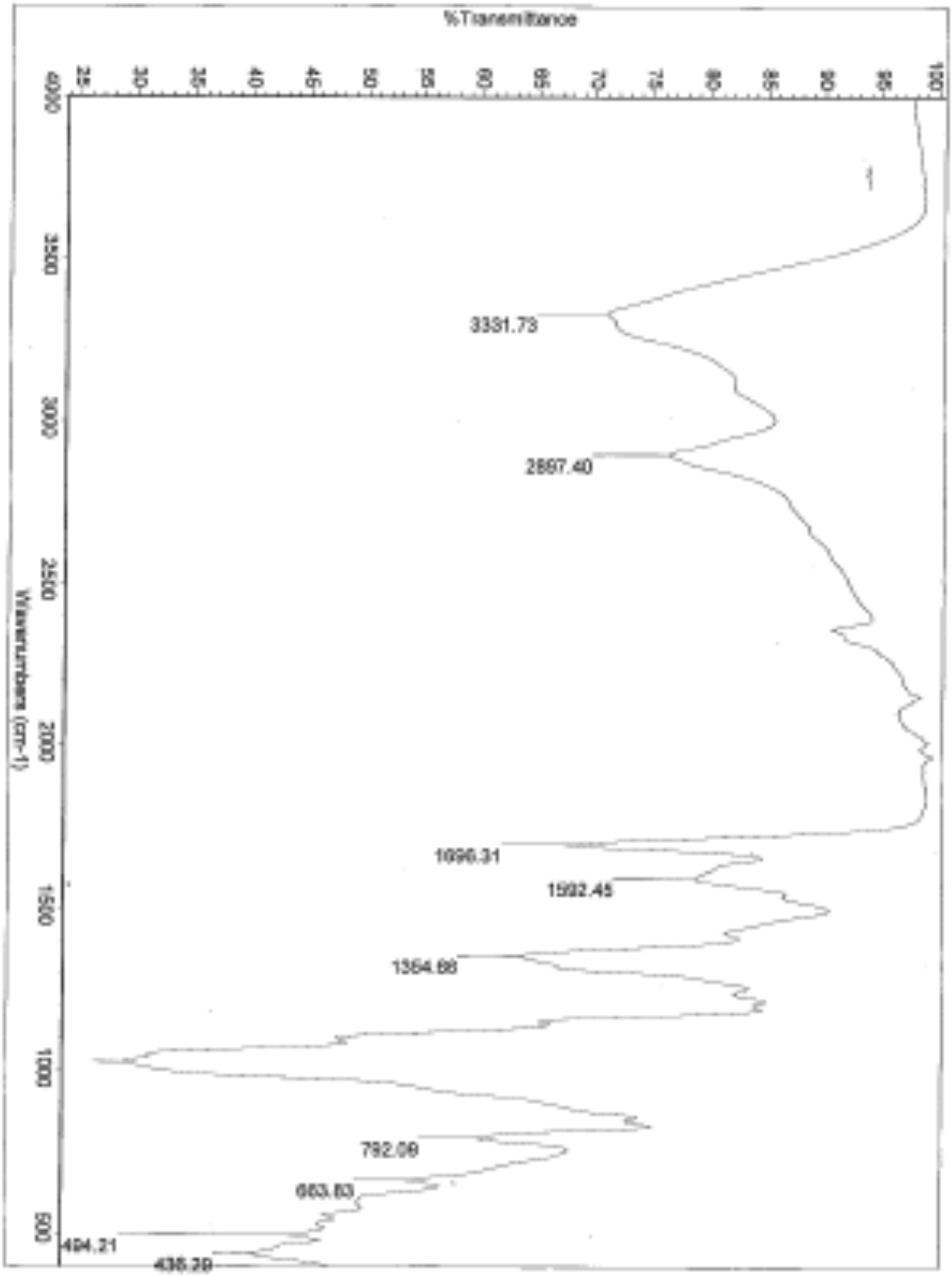

Figure S49. *FT-IR of formic acid fabricated NFC silylated with* ***1e***

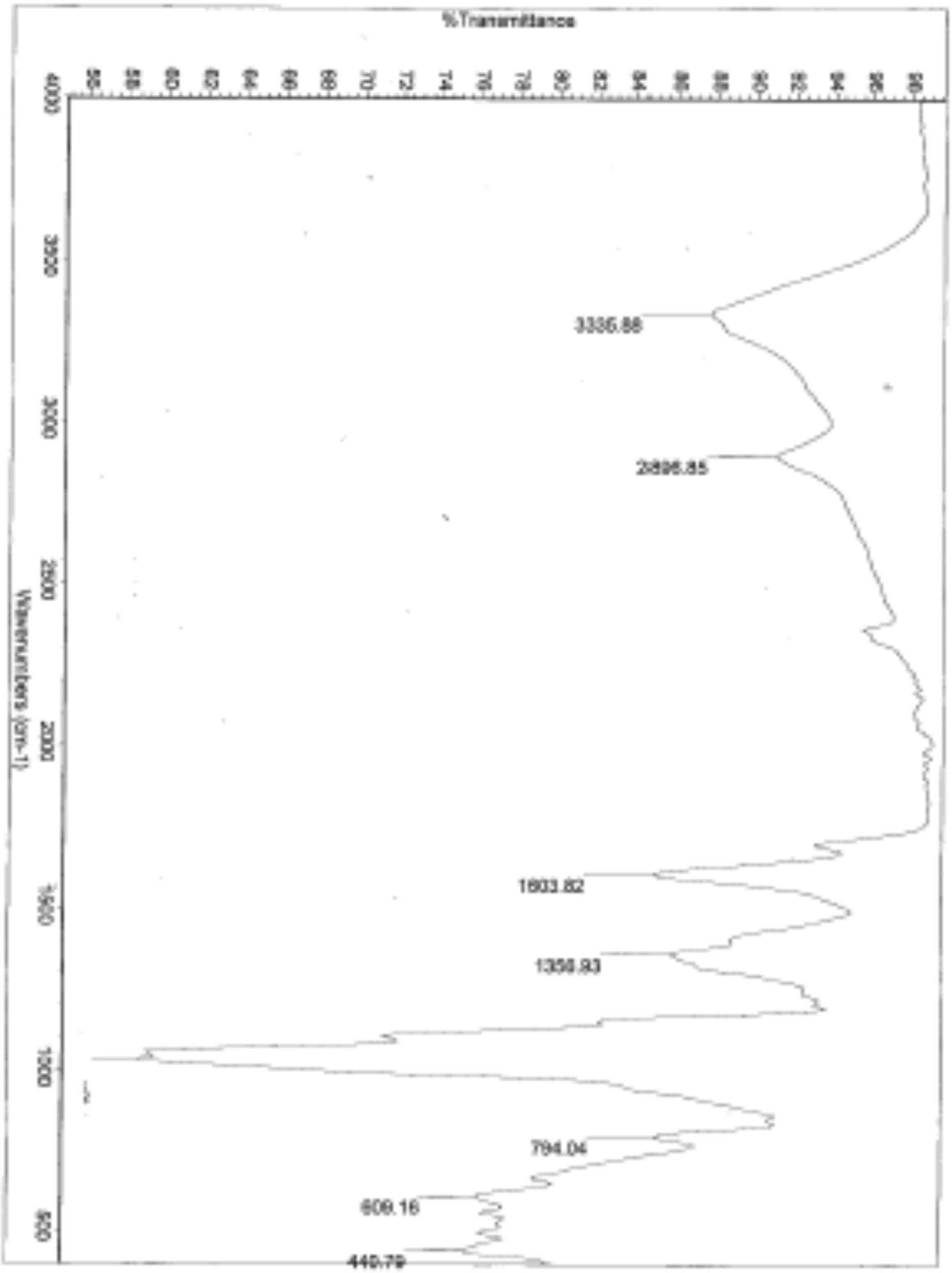

Figure S50. *FT-IR of NFC derived from TEMPO-NaClO oxidation-homogenization route silylated with **1e***

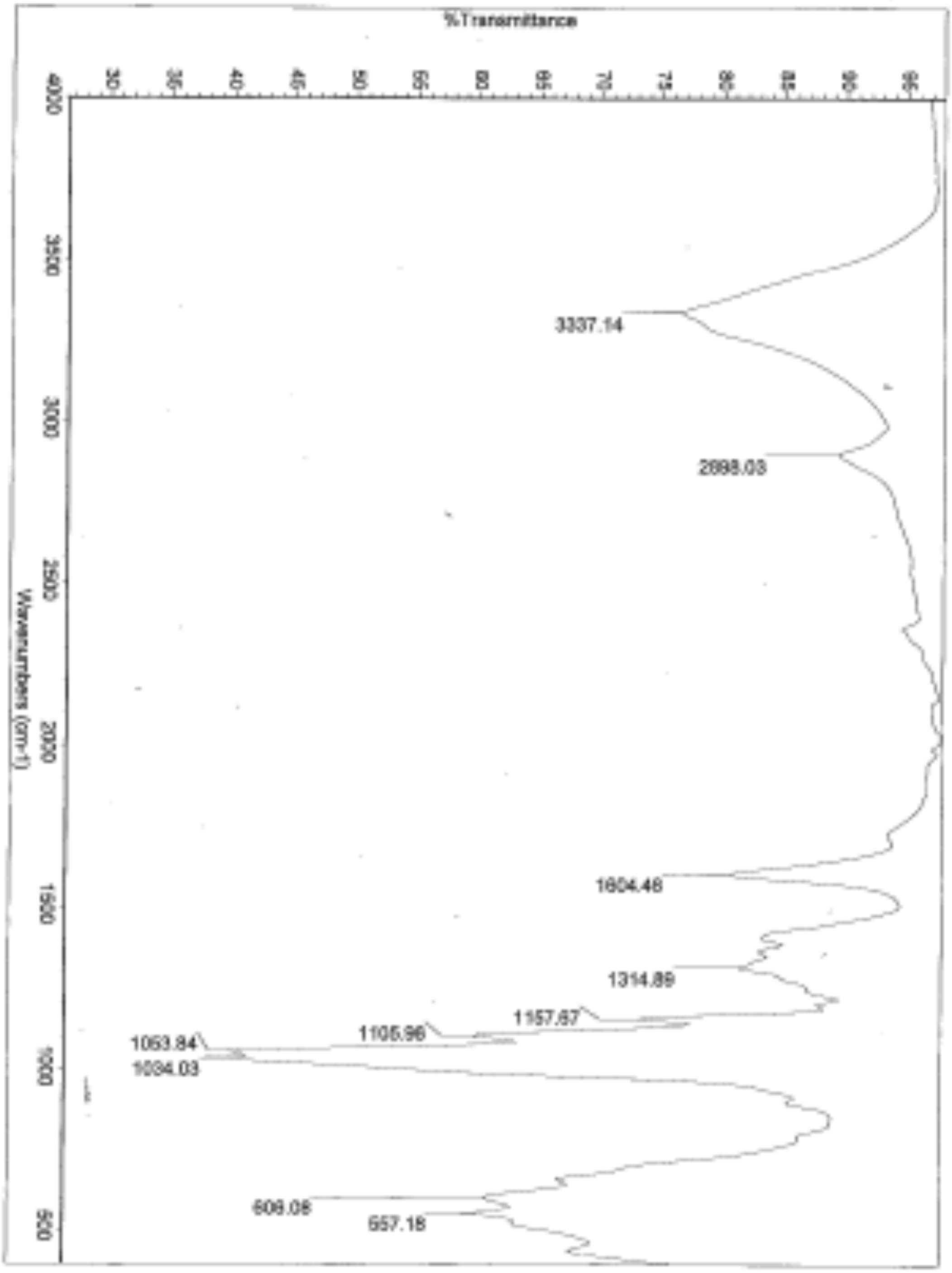

Figure S51. *FT-IR of NFC with equal amounts of **1a** and **1c** and it subsequent UV-treatment under "click" conditions. Formic acid fabricated NFC was used*

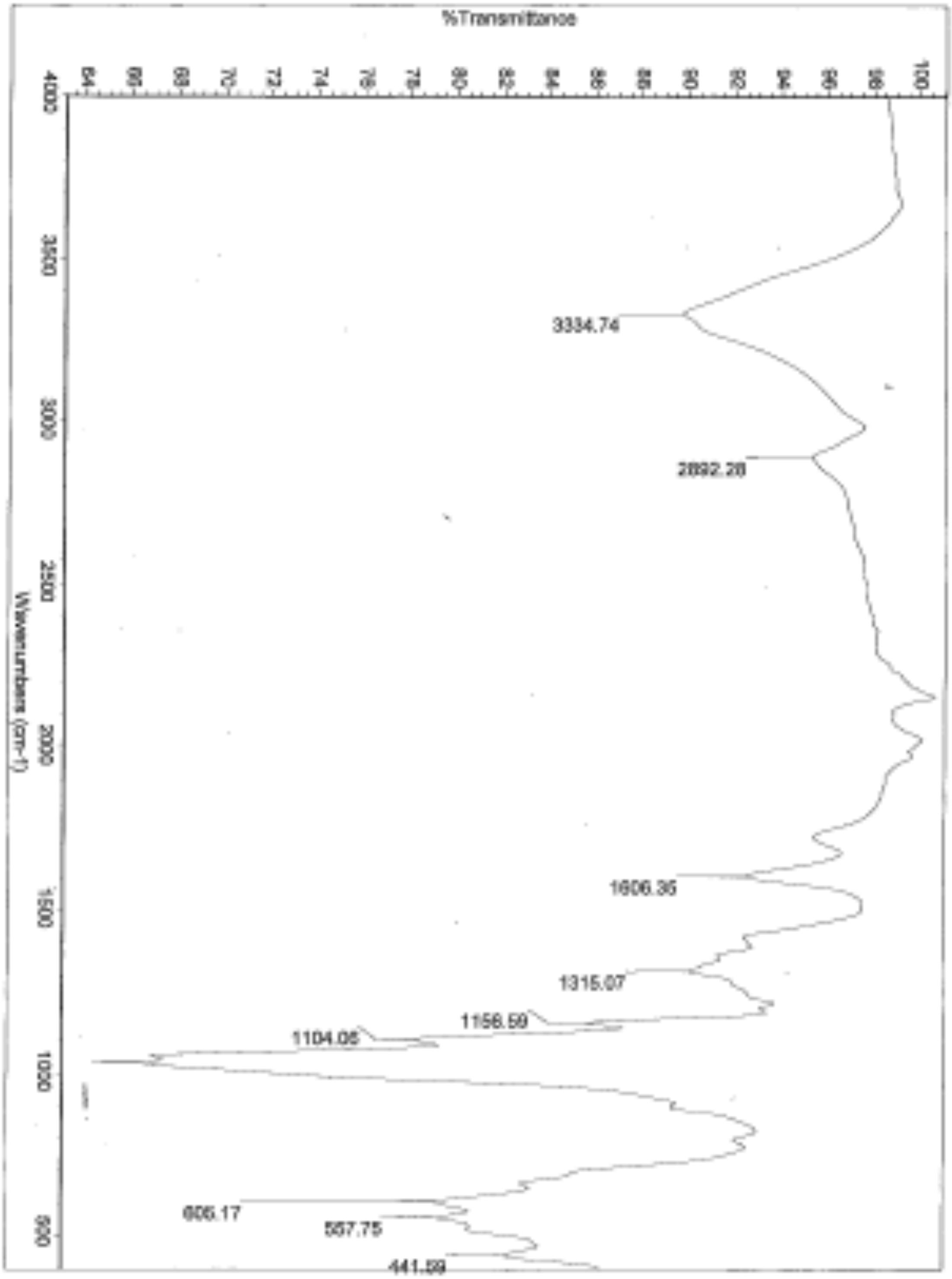

Figure S52. *FT-IR of NFC with equal amounts of **1a** and **1c** and it subsequent UV-treatment under "click" conditions. NFC derived from TEMPO-NaClO oxidation-homogenization route was used*

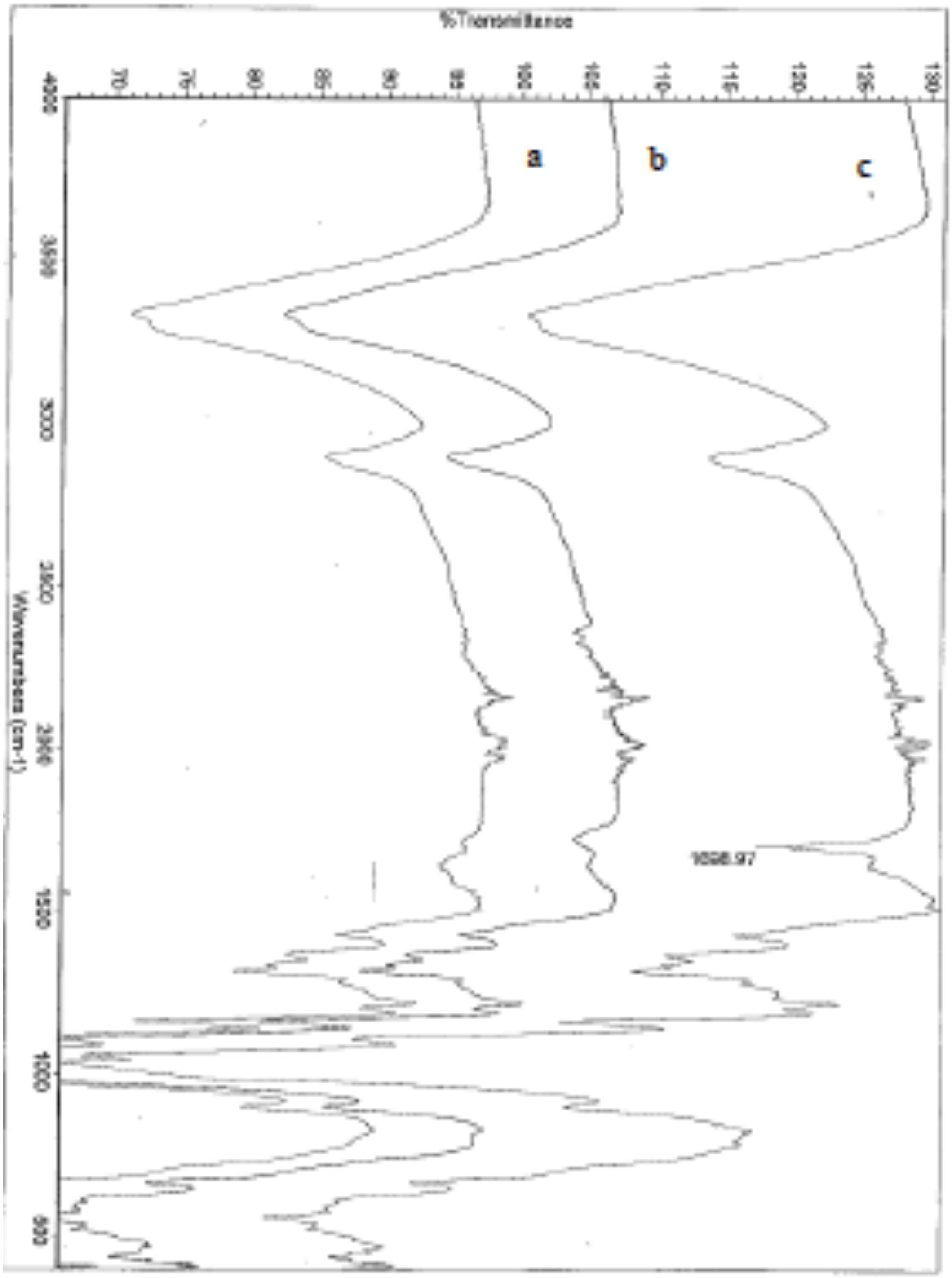

Figure S53. *FT-IR of click reaction between NFC silylated with* ***1c*** *and 6-mercaptohexan-1-ol-initated PCL:*

(a) Formic acid fabricated NFC, (b) NFC silylated with **1c**, (c) NFC after click reaction

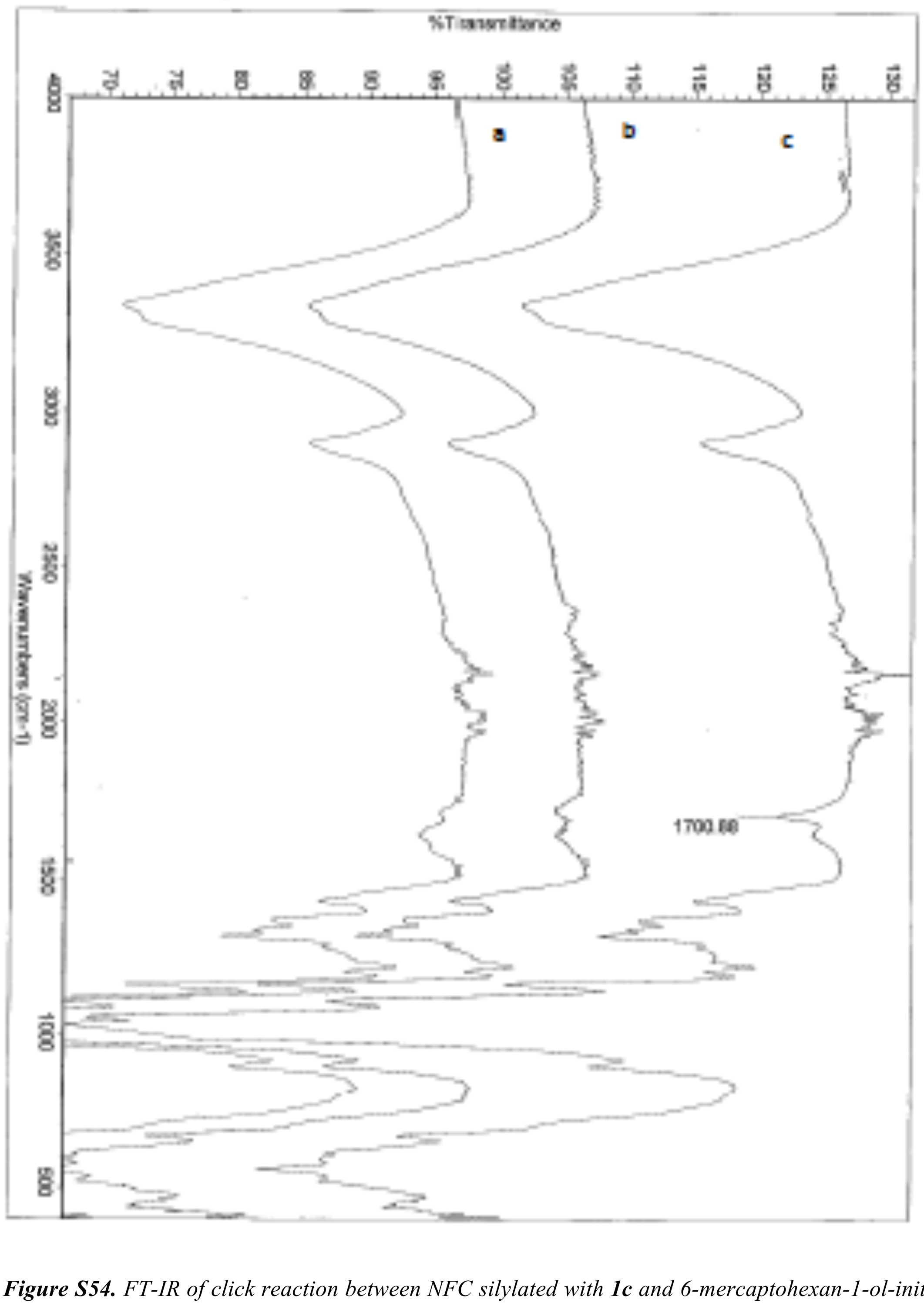

Figure S54. *FT-IR of click reaction between NFC silylated with* ***1c*** *and 6-mercaptohexan-1-ol-initated PVL:*

(a) Formic acid fabricated NFC, (b) NFC silylated with **1c**, (c) NFC after click reaction

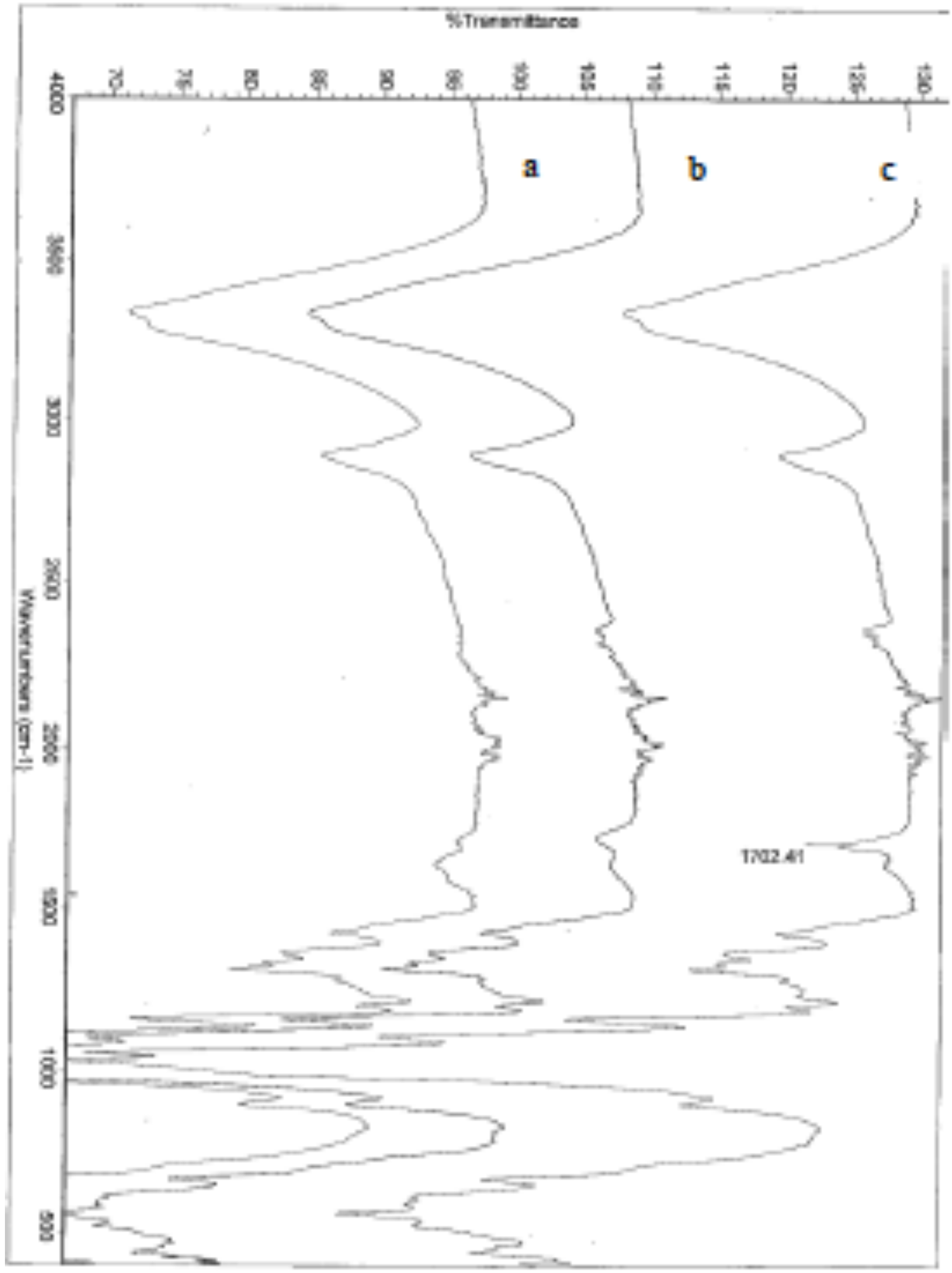

Figure S55. *FT-IR of click reaction between NFC silylated with* ***1c*** *and nonanoic acid-terminated-4-Pentene-1-ol-initiated PCL:*

(a) Formic acid fabricated NFC, (b) NFC silylated with **1c**, (c) NFC after click reaction

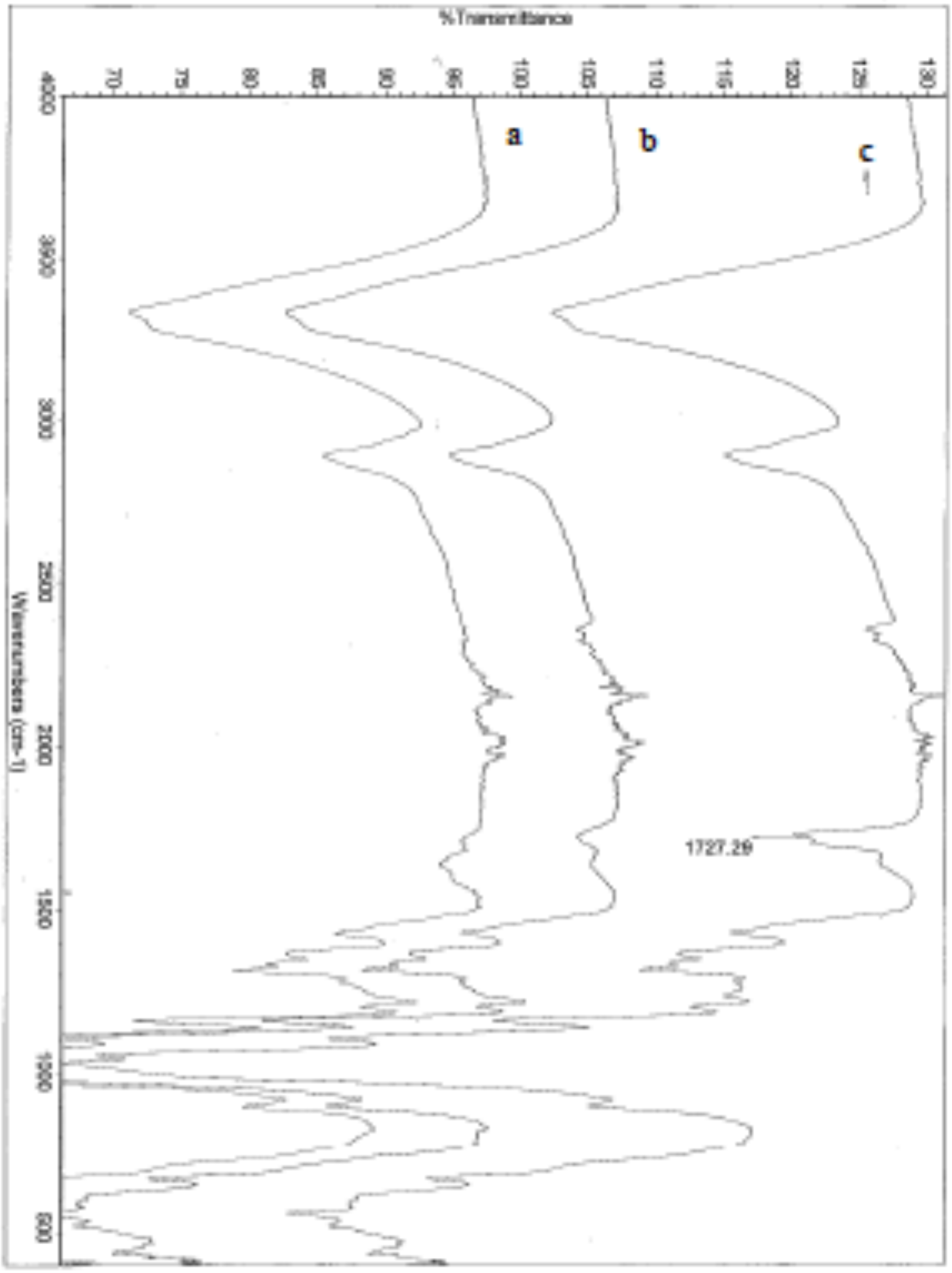

***Figure S56.** FT-IR of click reaction between NFC silylated with **1a** and 4-Pentene-1-ol-initiated-PCL:*
(a) Formic acid fabricated NFC, (b) NFC silylated with **1c**, (c) NFC after click reaction

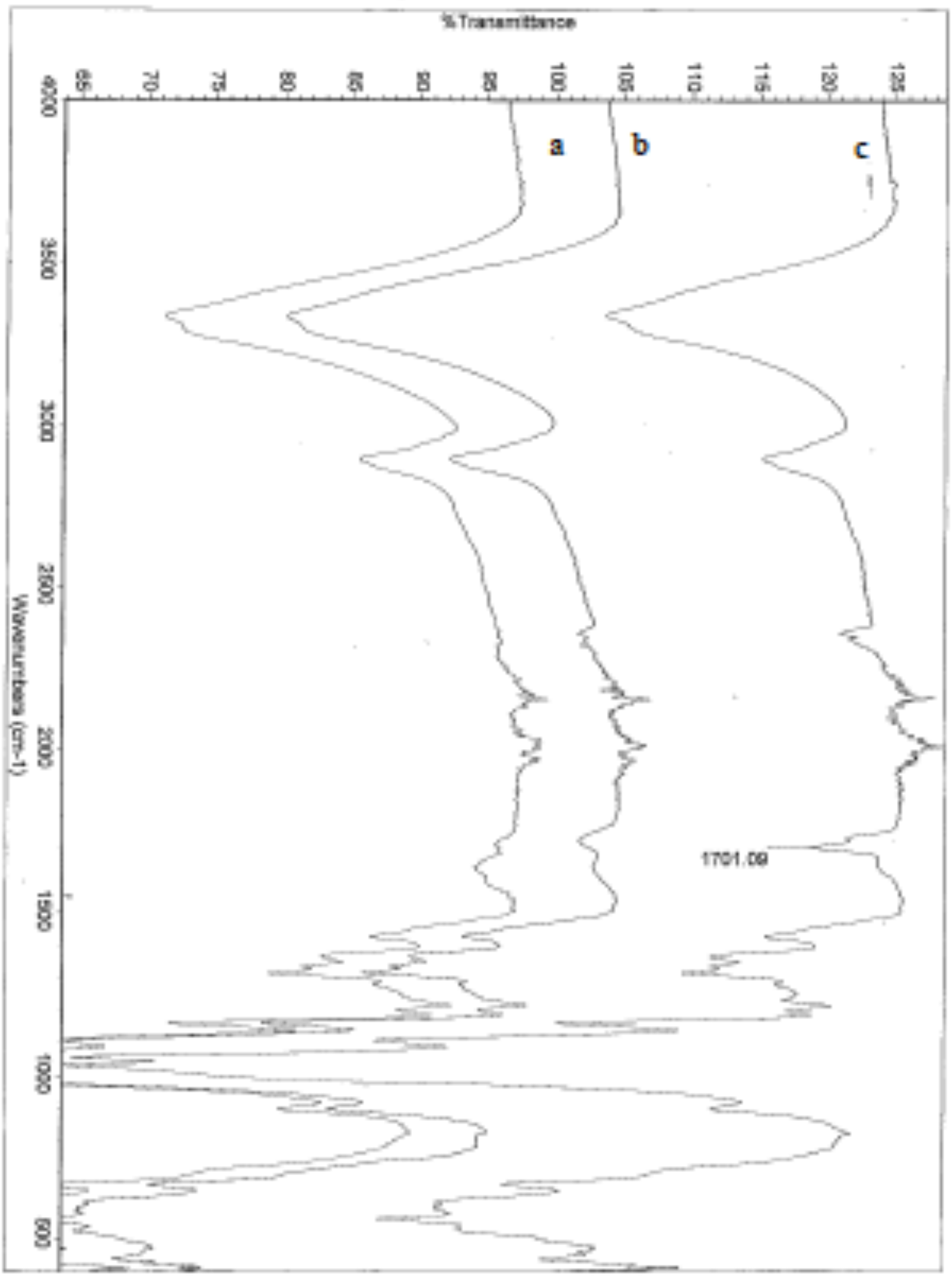

Figure S57. *FT-IR of click reaction between NFC silylated with **1a** and nonanoic acid-terminated-4-Pentene-1-ol-initiated PCL:*

(a) Formic acid fabricated NFC, (b) NFC silylated with **1c**, (c) NFC after click reaction

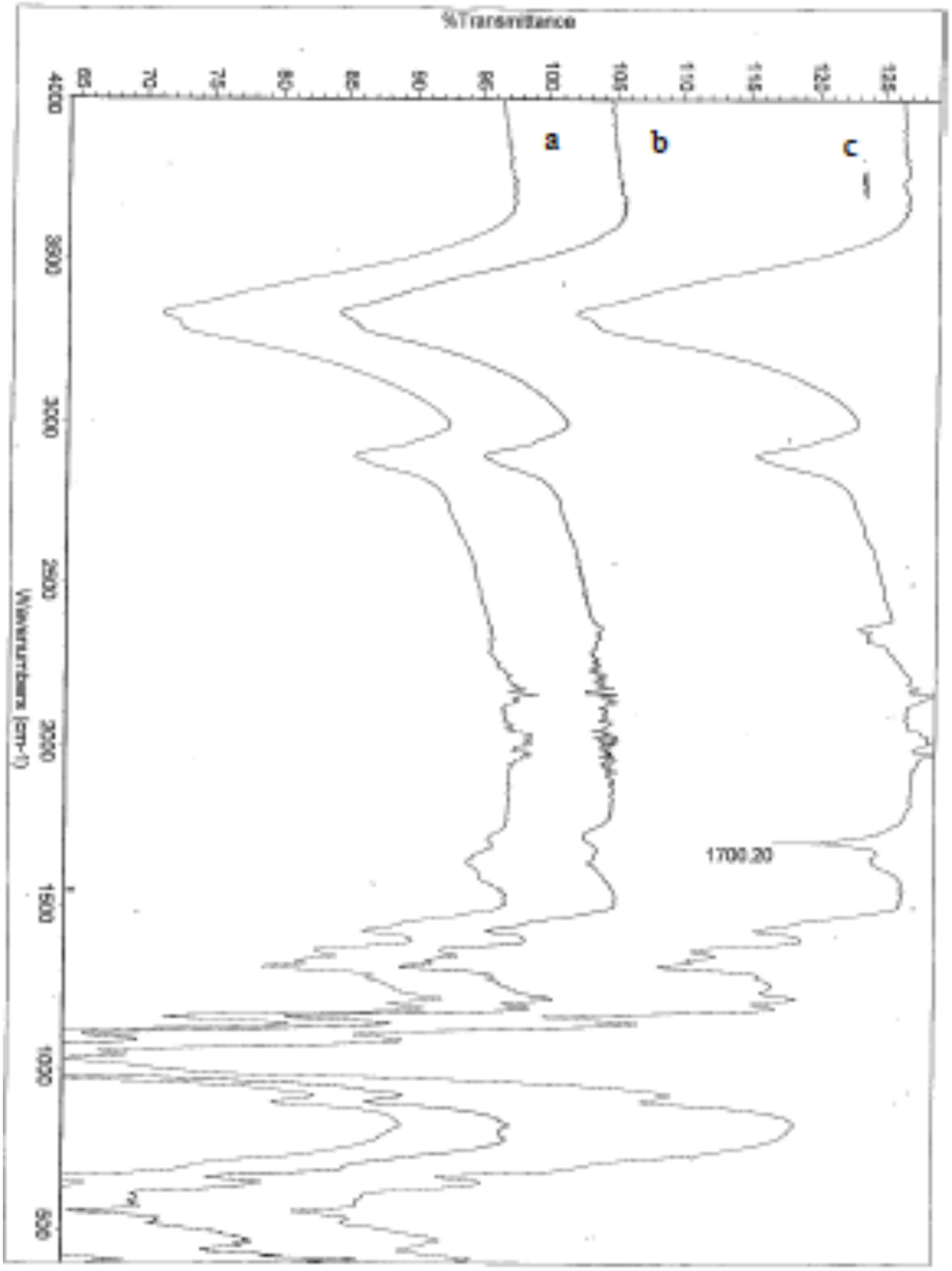

Figure S58. *FT-IR of click reaction between NFC silylated with* ***1a*** *and 4-Pentene-1-ol-initiated-PVL:*

(a) Formic acid fabricated NFC, (b) NFC silylated with **1c**, (c) NFC after click reaction

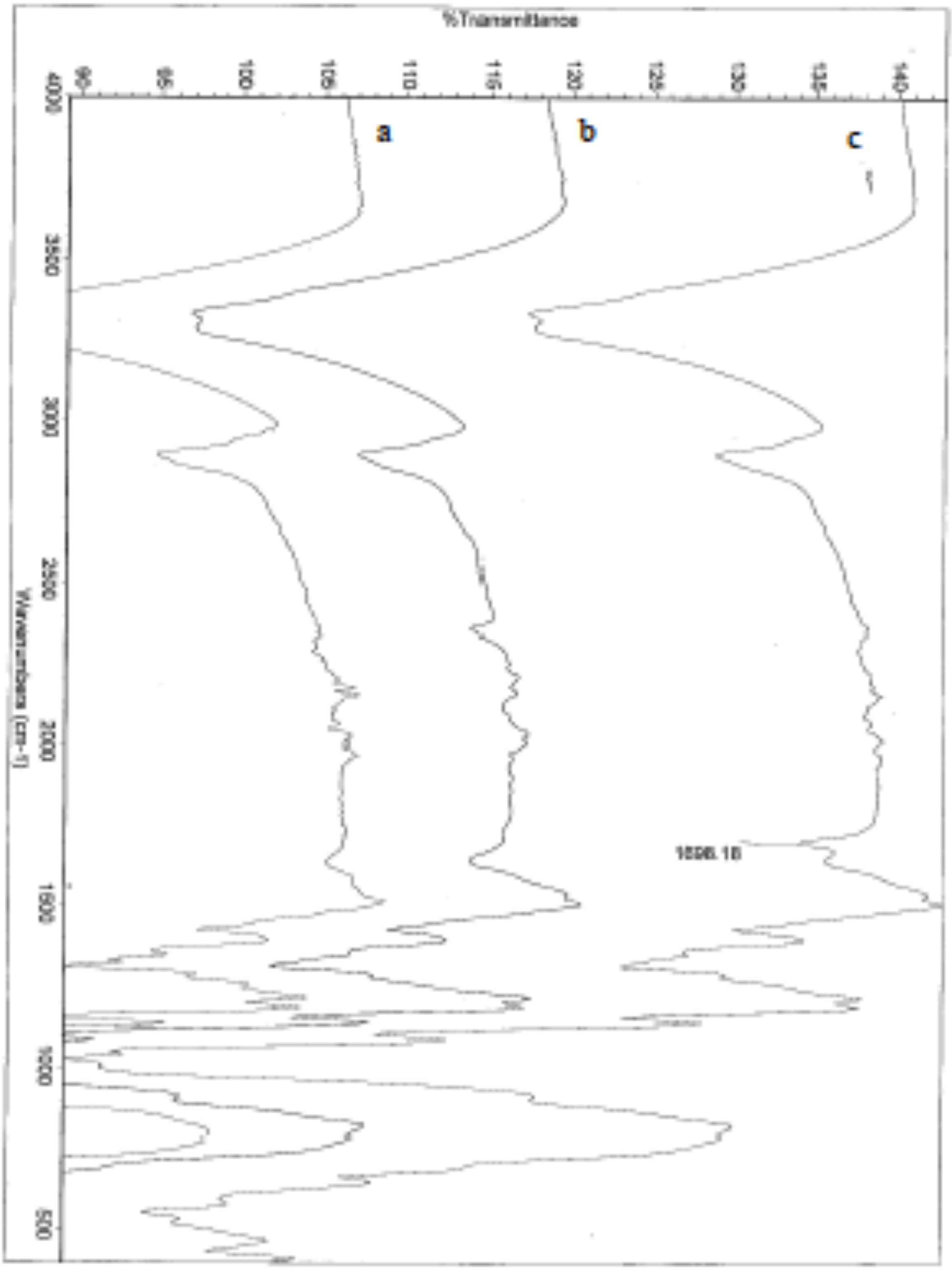

Figure S59. *FT-IR of click reaction between filter paper silylated with **1a** and 4-Pentene-1-ol-initiated-PVL:*

(a) Filter paper, (b) Filter paper silylated with **1a**, (c) Filter paper after click reaction

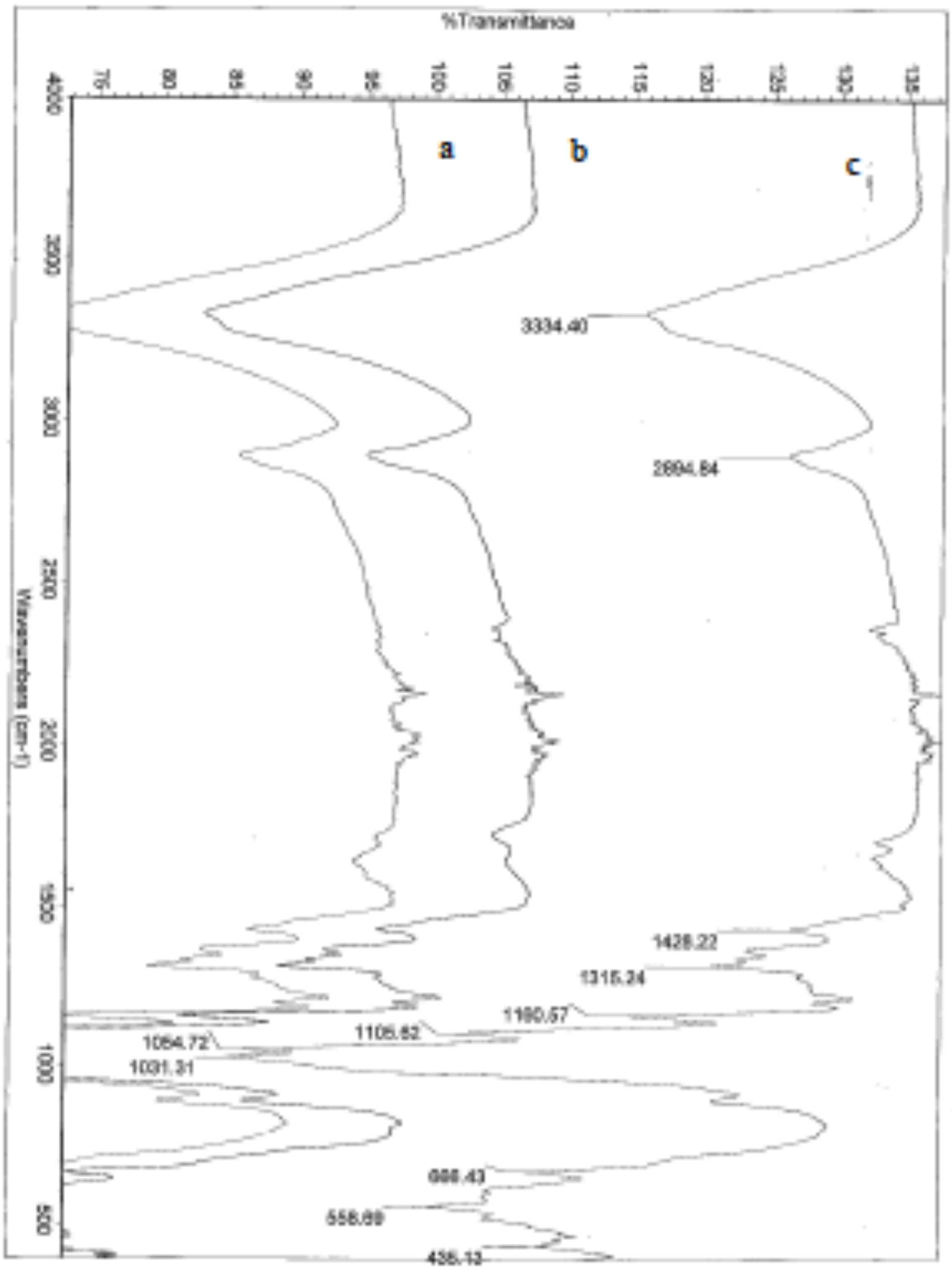

Figure S60. *FT-IR of click reaction between NFC silylated with **1a** and Quinidine:*

(a) Formic acid fabricated NFC, (b) **1a**-NFC, (c) **1a**-NFC after click reaction Quinidine.

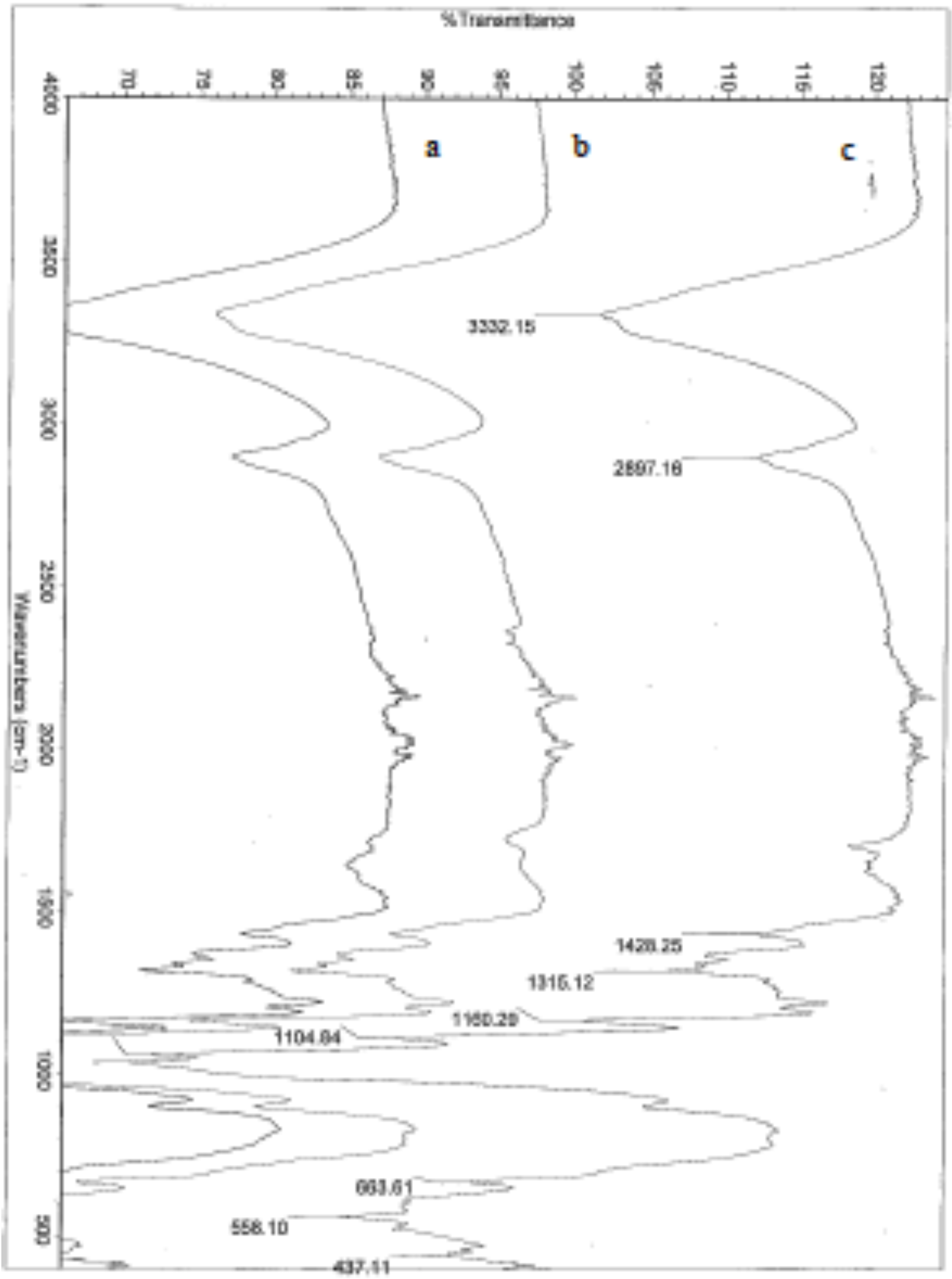

Figure S61. *FT-IR of click reaction between NFC silylated with **1a** and Quinine:*

(a) Formic acid fabricated NFC, (b) **1a**-NFC (c) **1a**-NFC after click reaction with Quinine.

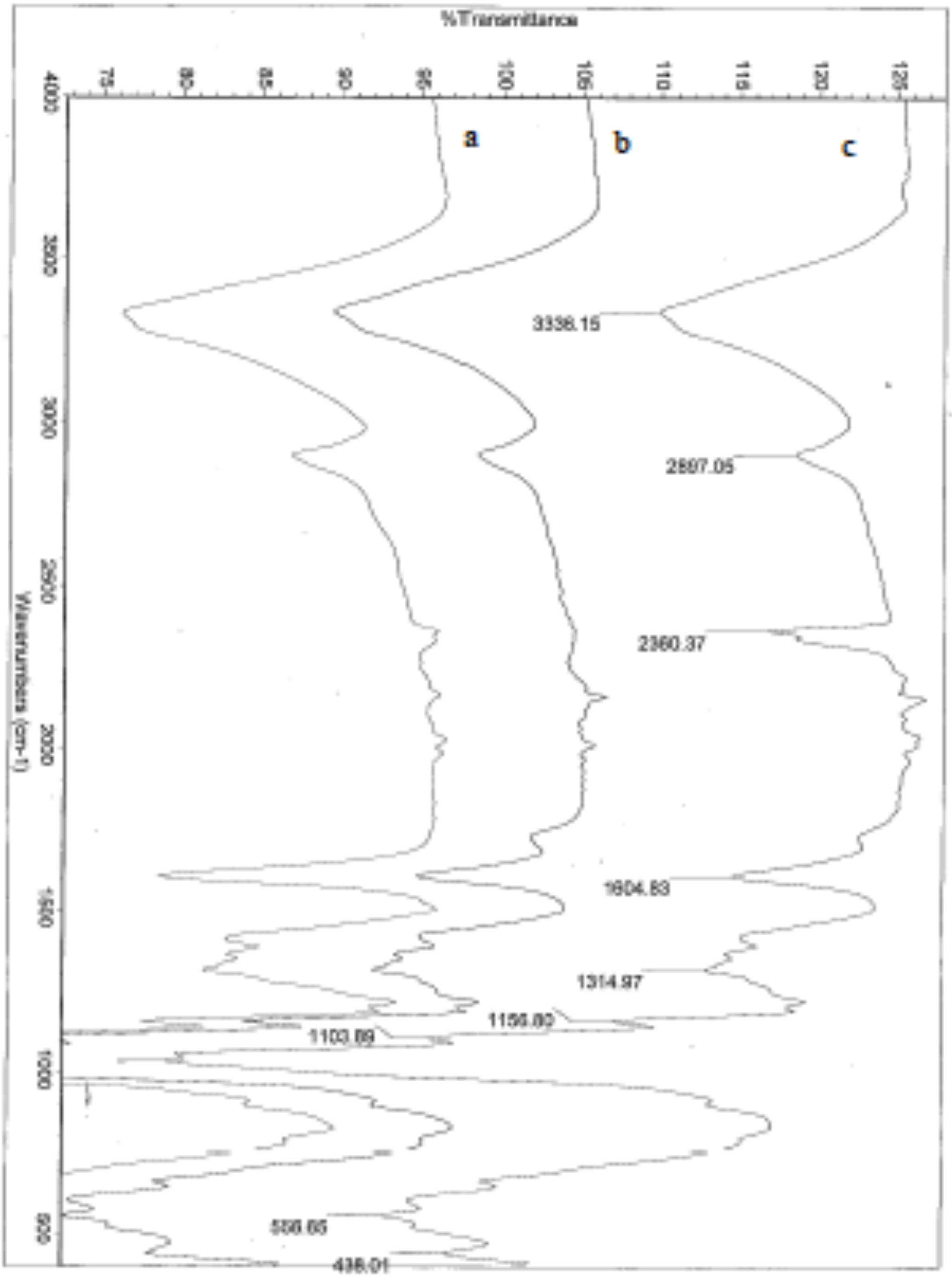

Figure S62. *FT-IR of click reaction between NFC silylated with* ***1a*** *and Quinidine:*

(a) NFC derived from TEMPO-NaClO oxidation-homogenization route Formic acid fabricated NFC, (b) **1a**-NFC (c) **1a**-NFC after click reaction Quinidine

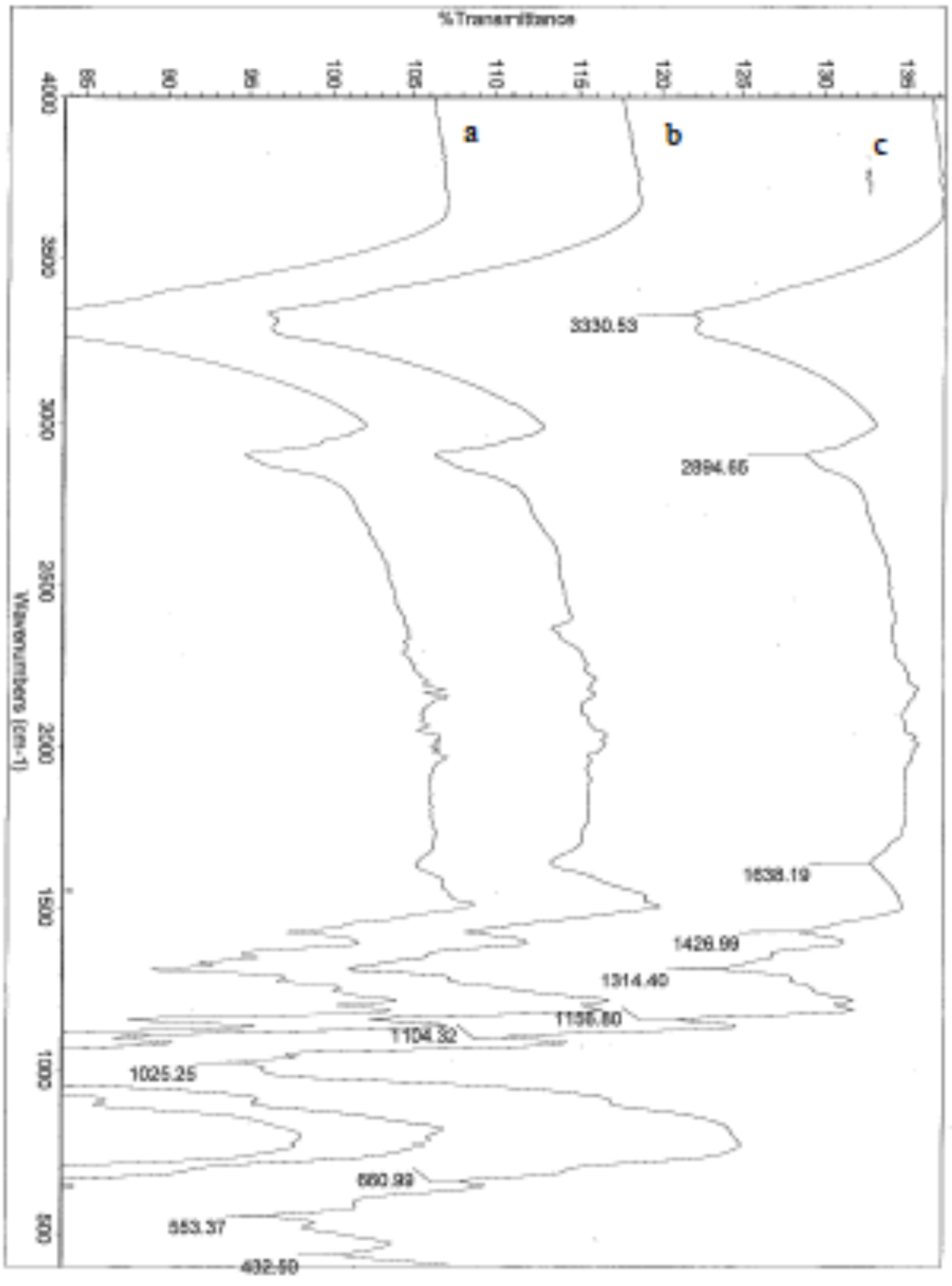

Figure S63. *FT-IR of click reaction between filter paper silylated with **1a** and Quinine:*

(a) Filter paper, (b) Filter paper silylated with **1a**, (c) Filter paper after click reaction

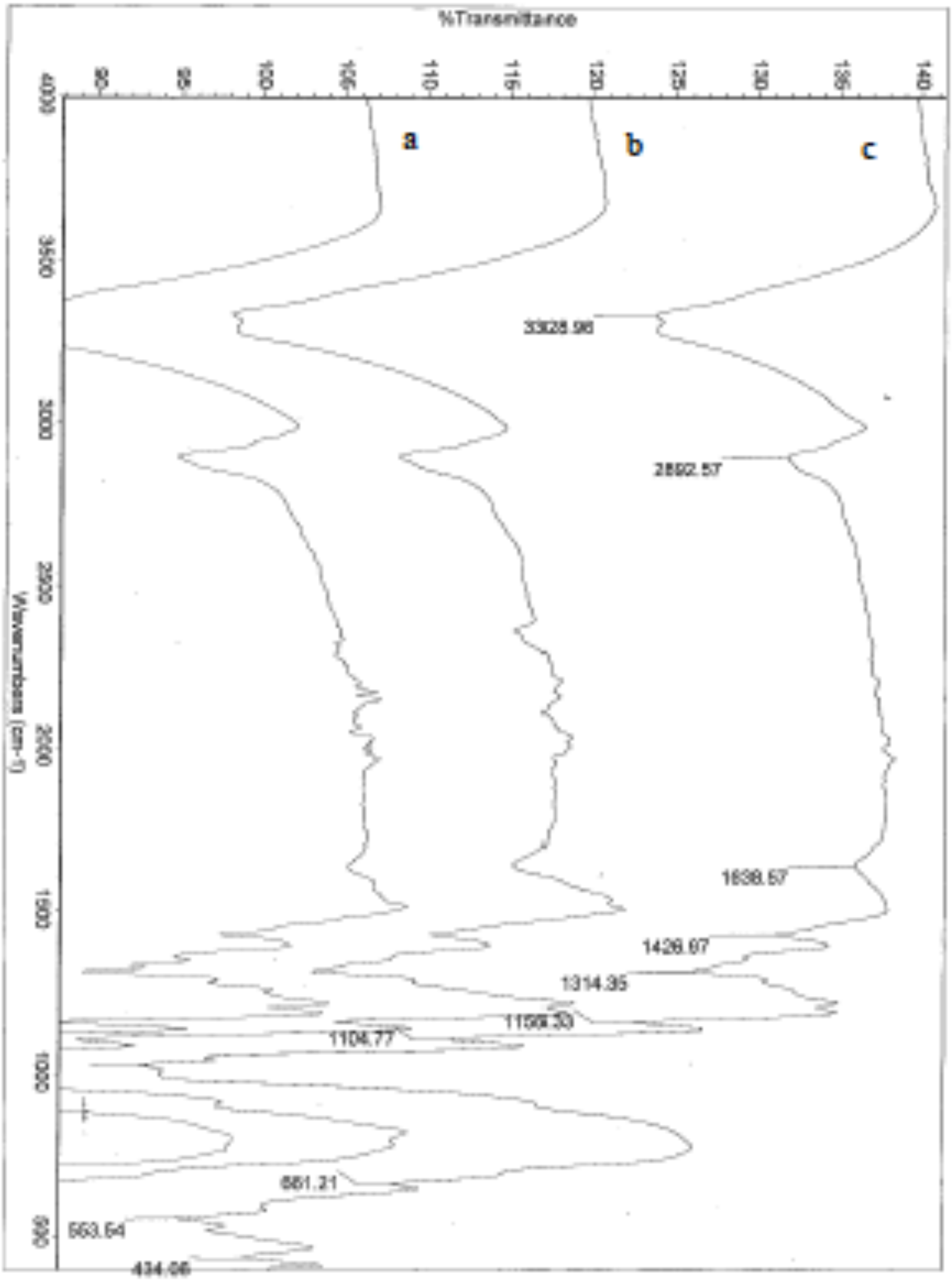

Figure S64. *FT-IR of click reaction between filter paper silylated with* ***1a*** *and Quinidine:*

(a) Filter paper, (b) Filter paper silylated with **1a**, (c) Filter paper after click reaction

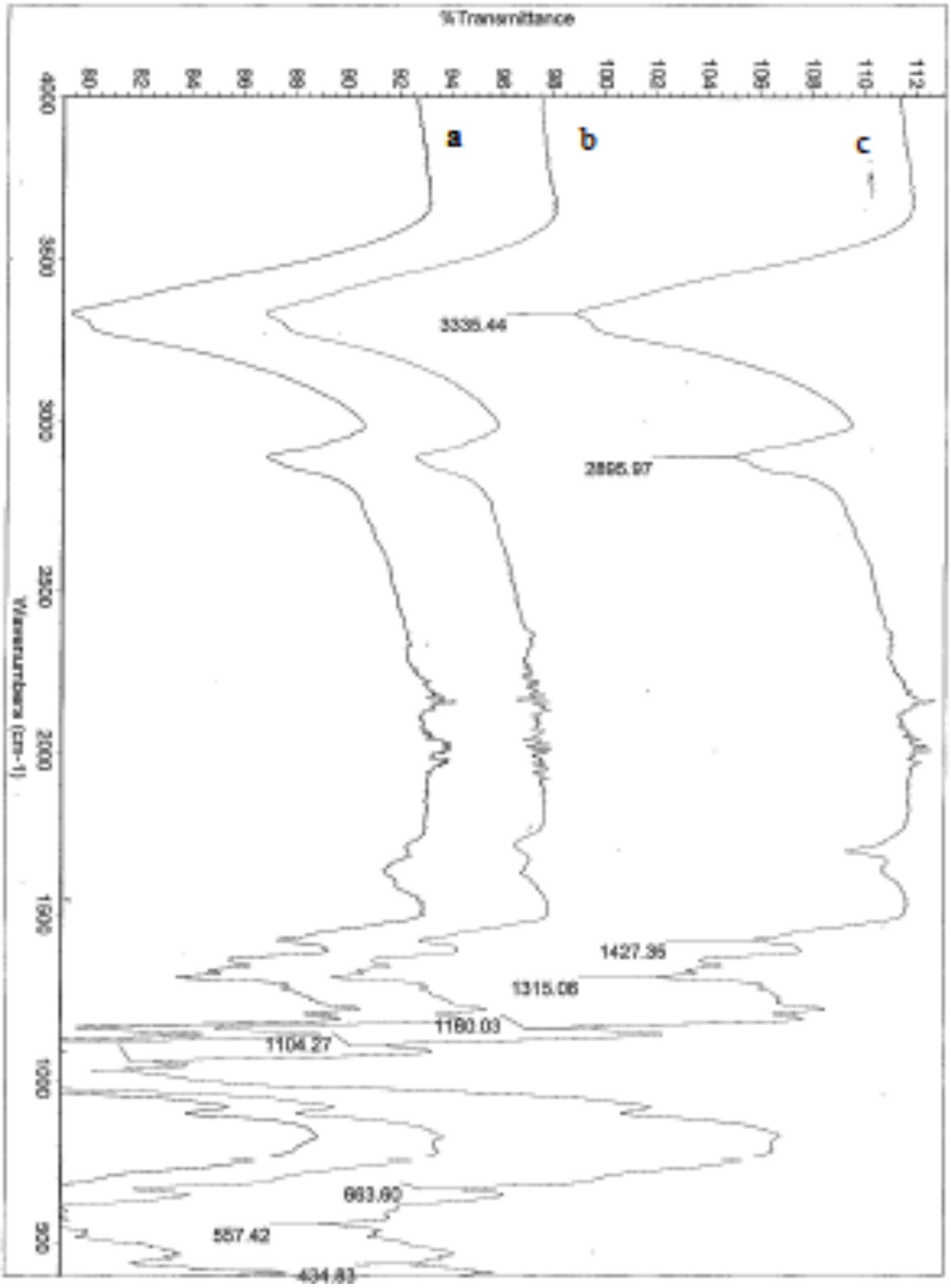

Figure S65. *FT-IR of click reaction between NFC silylated with **1c** and 1-octanethiol:*

(a) Formic acid fabricated NFC, (b) **1c**-NFC, (c) **1c**-NFC after click reaction with 1-octanethiol

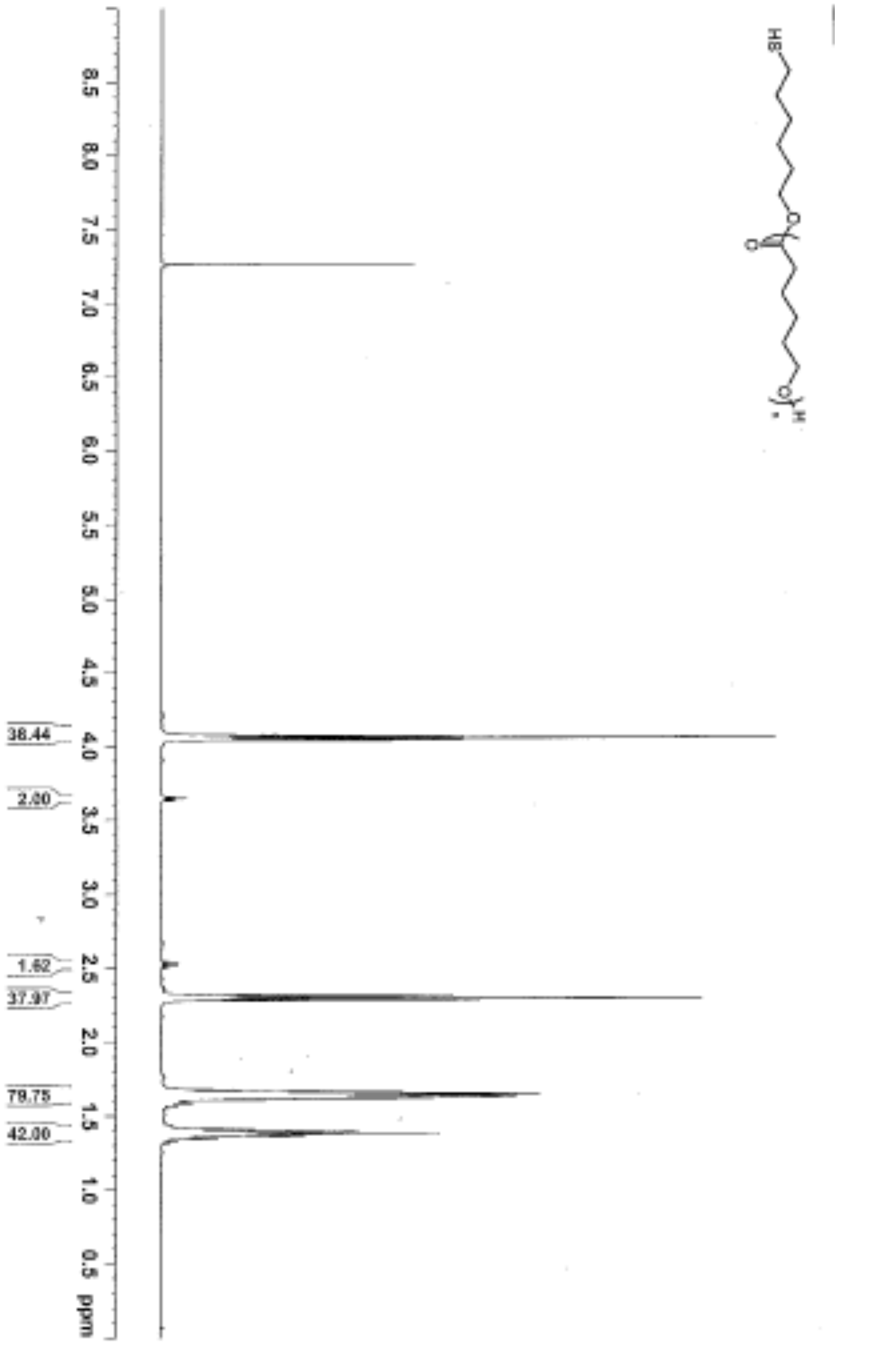

Figure S66. *^{1}H NMR spectrum of 6-Mercaptohexan-1-ol-initated-PCL*

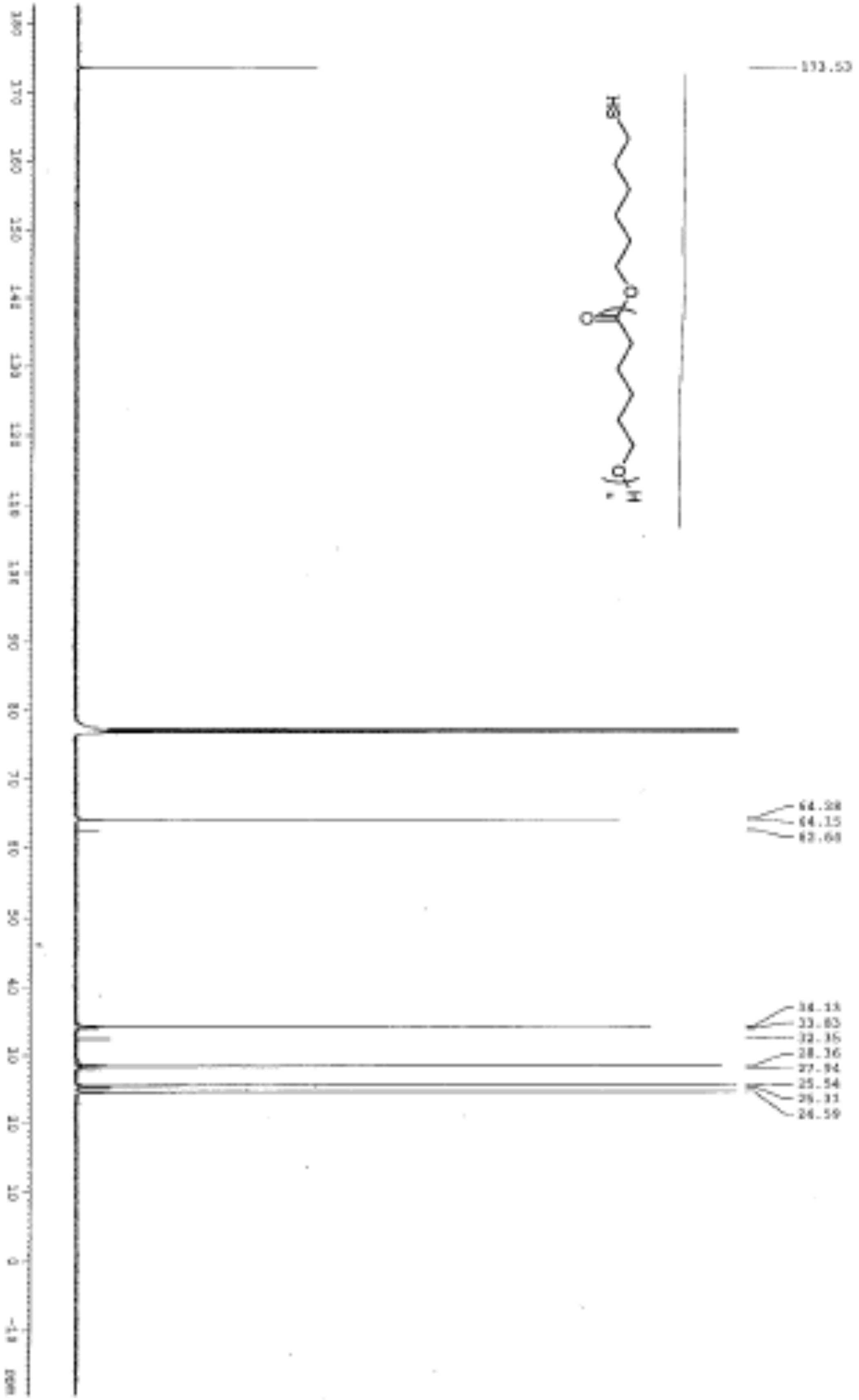

Figure S67. ^{13}C NMR spectrum of 6-Mercaptohexan-1-ol-initated-PCL

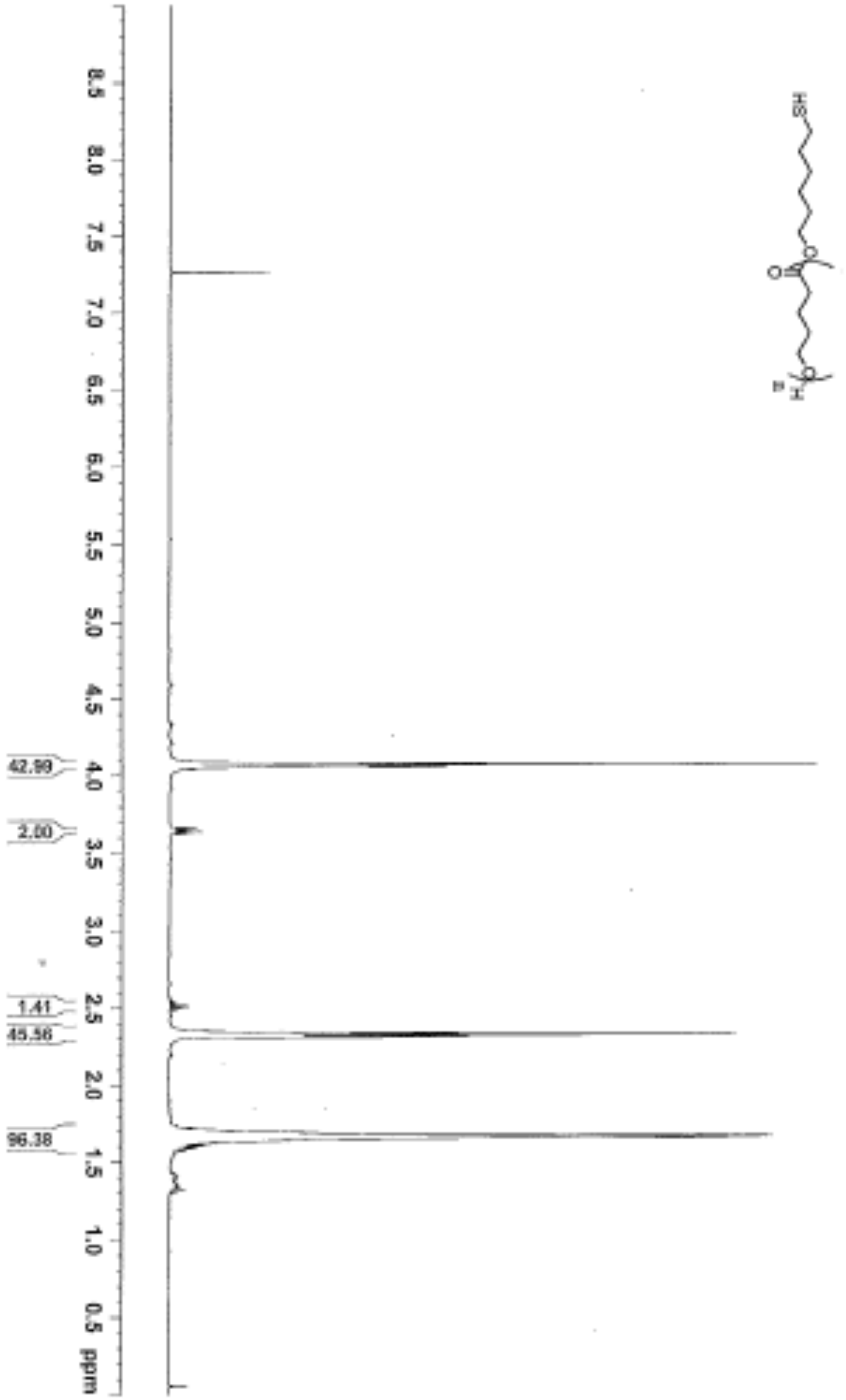

Figure S68. *^{1}H NMR spectrum of 6-Mercaptohexan-1-ol-initated-PVL*

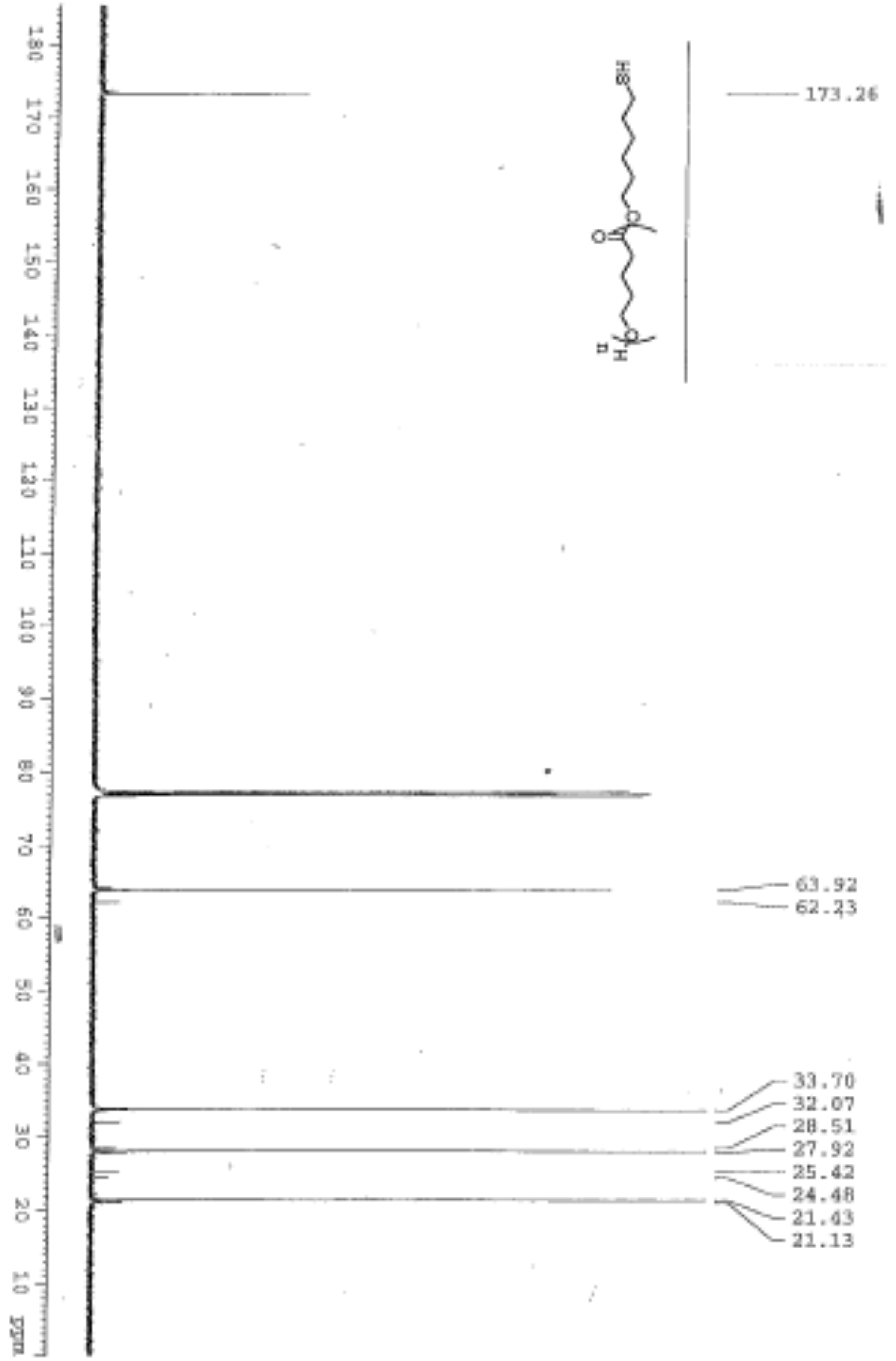

Figure S69. *^{13}C NMR spectrum of 6-Mercaptohexan-1-ol-initated-PVL*

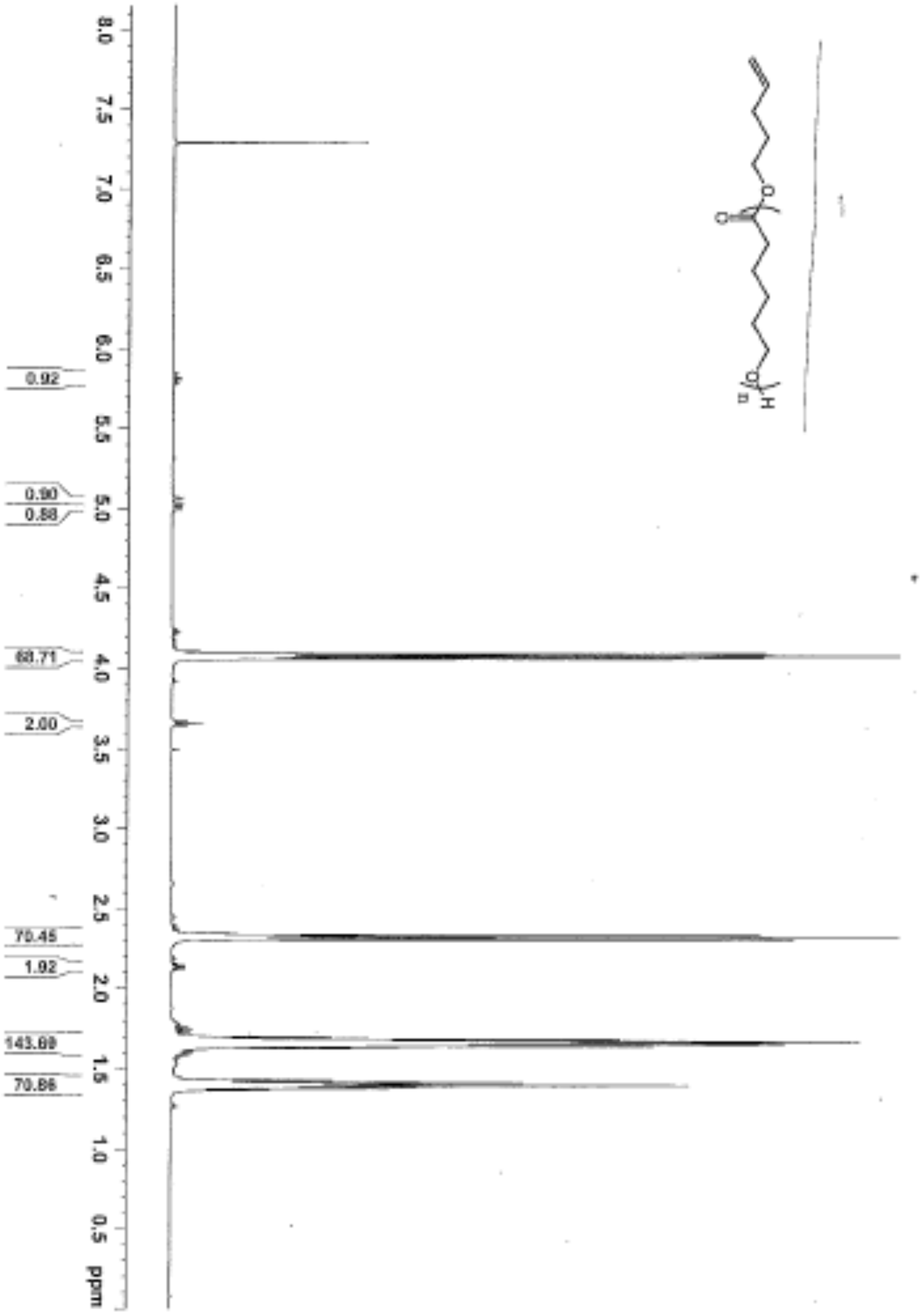

Figure S70. *^{1}H NMR spectrum of 4-Pentene-1-ol-initated-PCL*

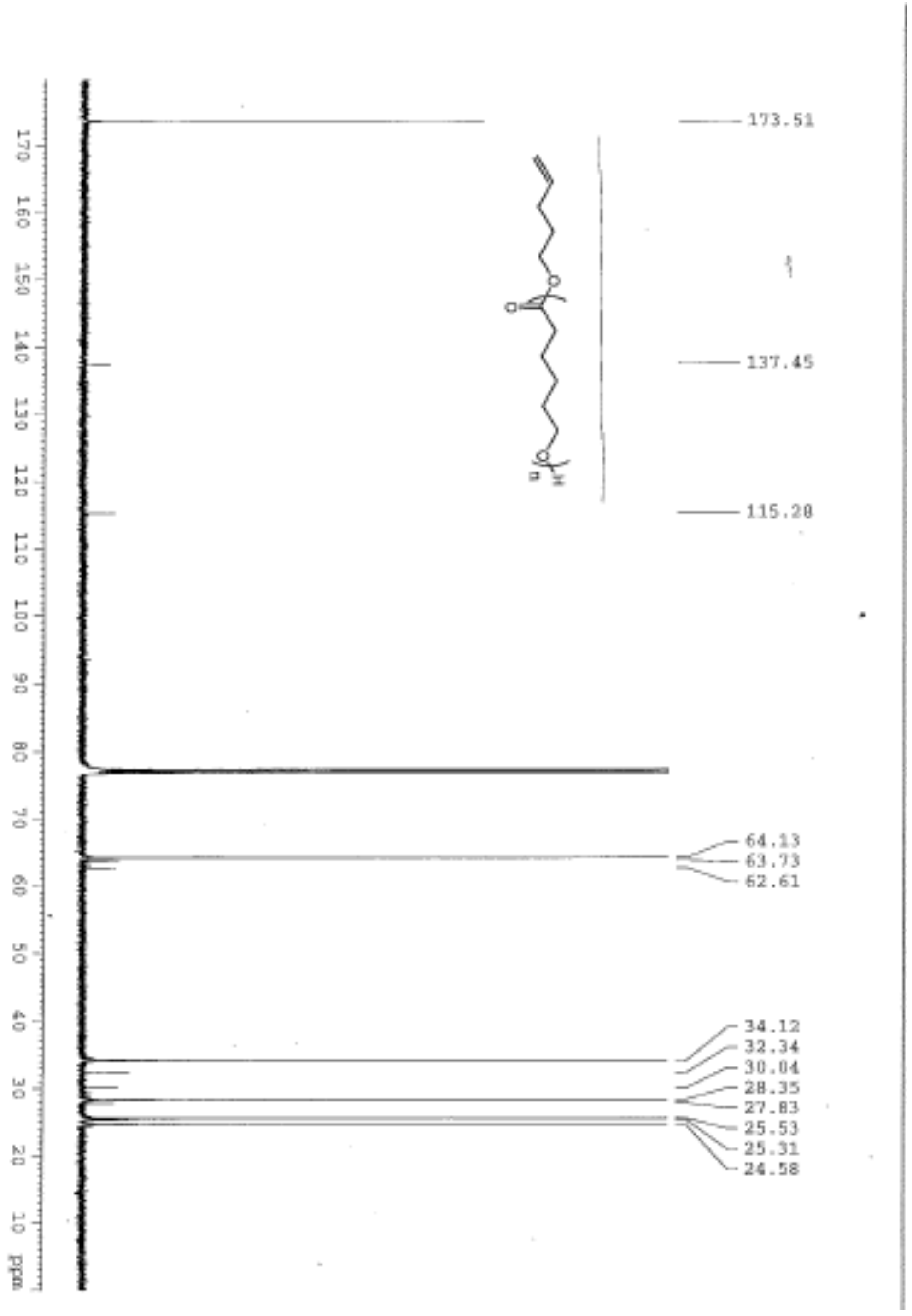

Figure S71. *^{13}C NMR spectrum of 4-Pentene-1-ol-initated-PCL*

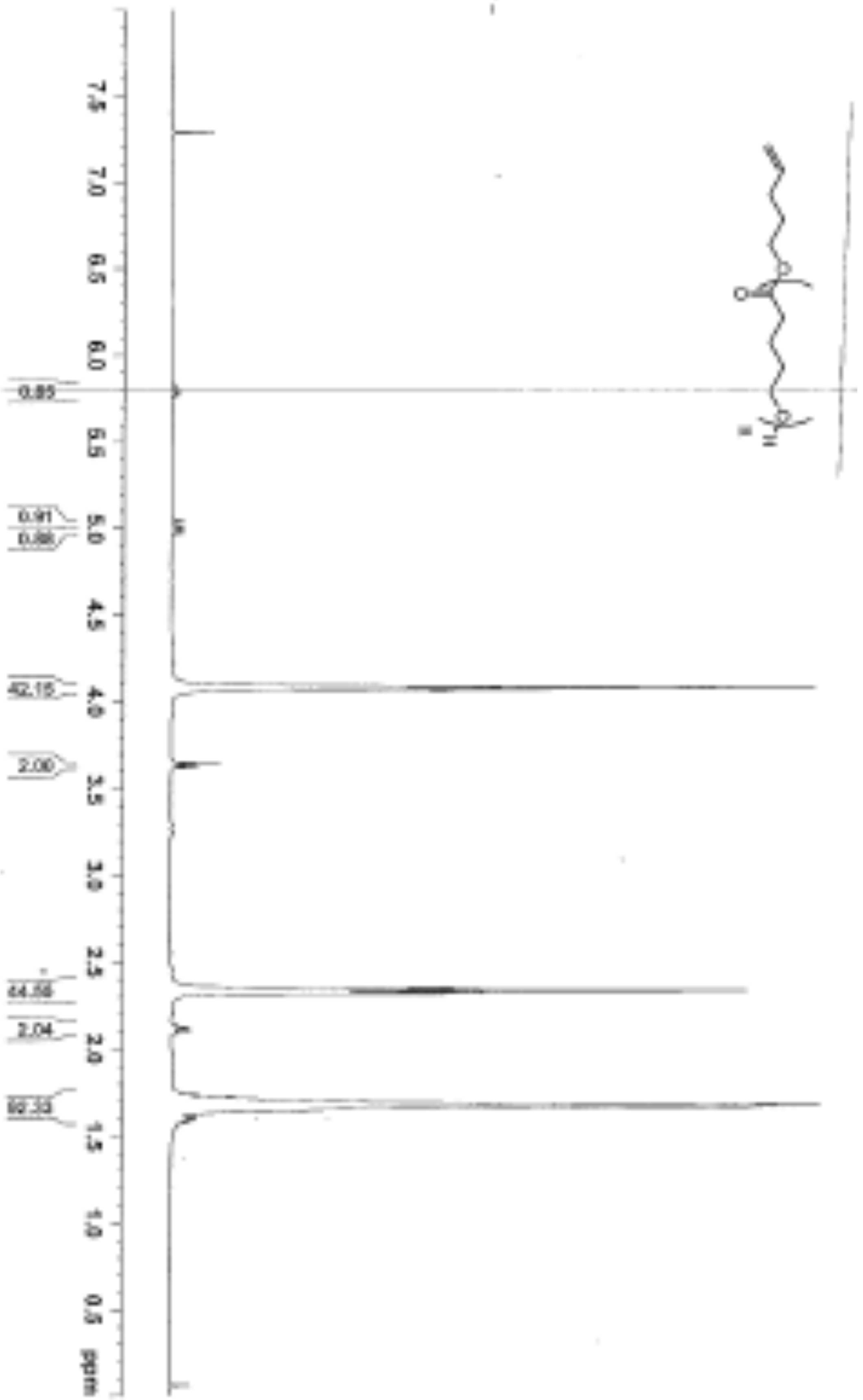

Figure S72. *^{1}H NMR spectrum of 4-Pentene-1-ol-initated-PVL*

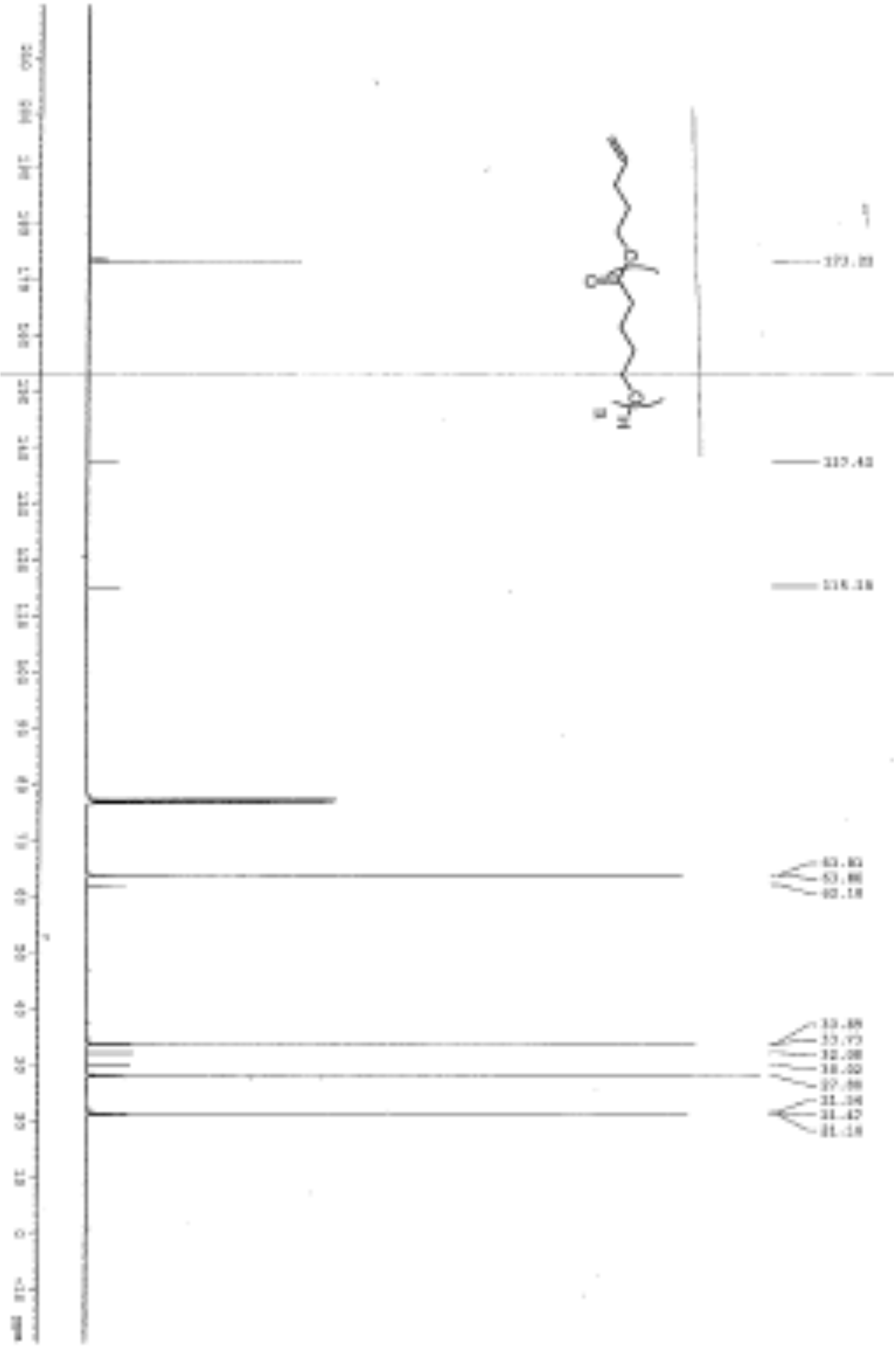

Figure S73. *^{13}C NMR spectrum of 4-Pentene-1-ol-initated-PVL*

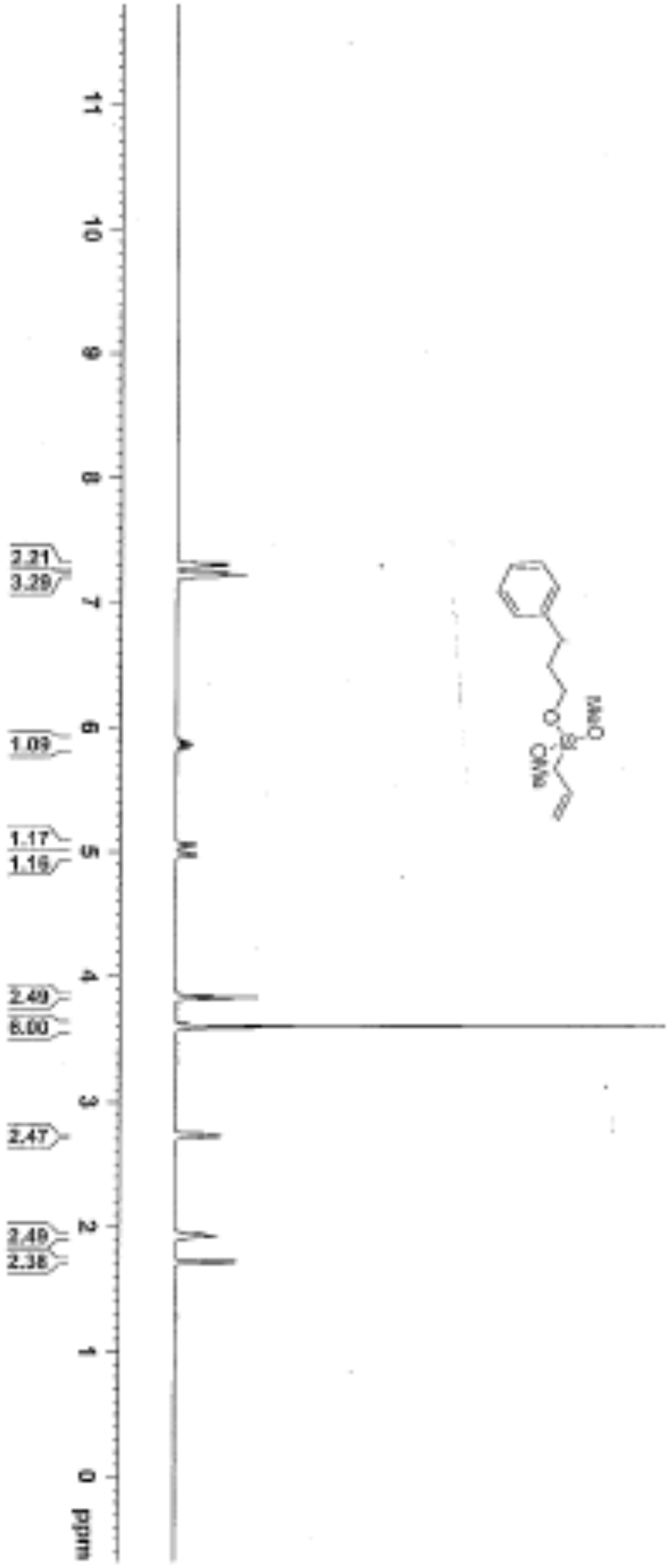

Figure S74. *^{1}H NMR spectrum of allyldimethoxy(3-phenylpropoxy)silane*

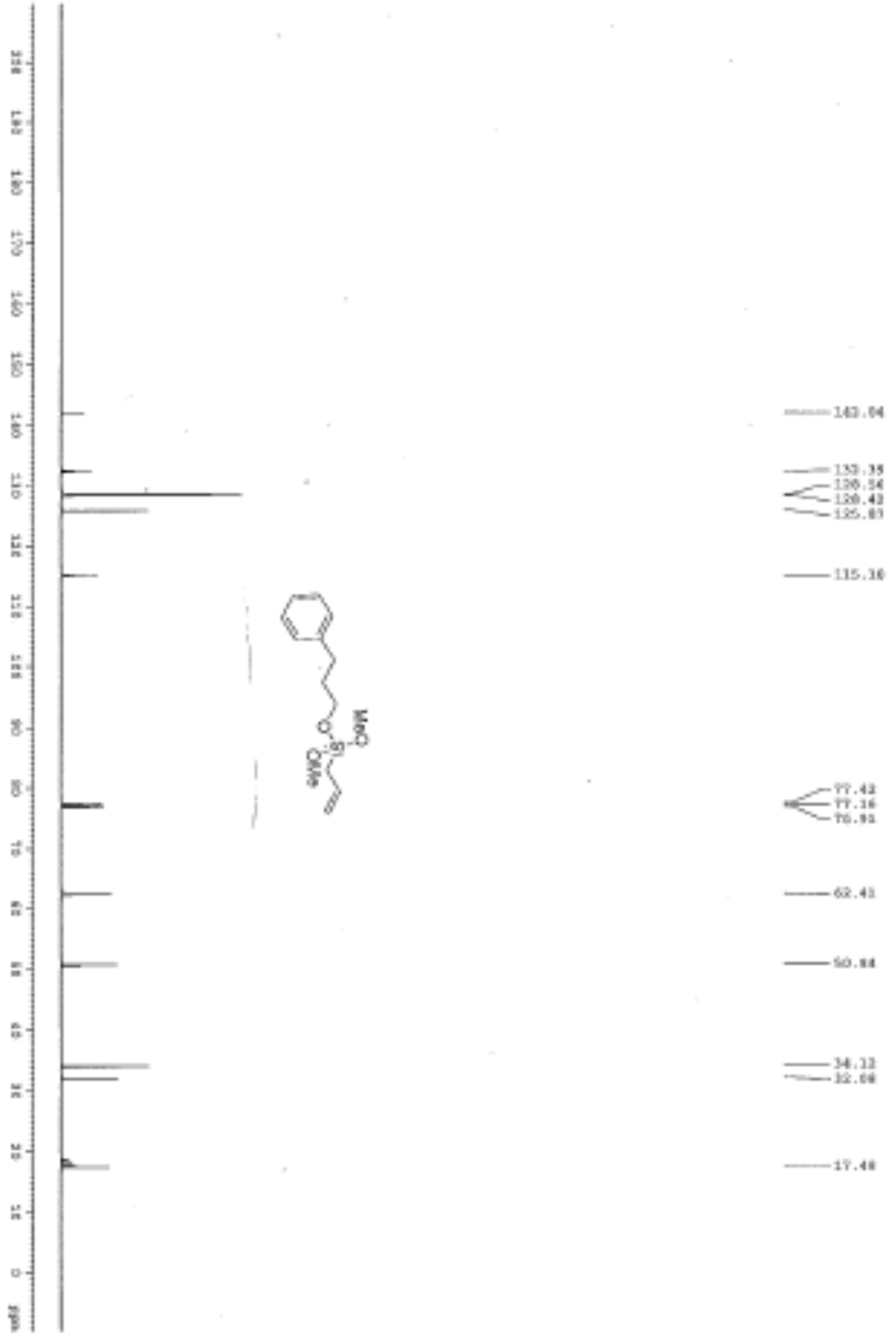

Figure S75. *^{13}C NMR spectrum of allyldimethoxy(3-phenylpropoxy)silane*

Qualitative Compound Report

Data File	[illegible]	Sample Name	[illegible]
Sample Type	Sample	Position	[illegible]
Instrument Name	Q-TOF	User Name	[illegible]
Acq Method	[illegible]	Acquired Time	[illegible]
IRM Calibration Status	[illegible]	DA Method	default.m
Comment			

Acquisition SW	[illegible]
Version	[illegible]

Compound Table

Compound Label	RT	Mass	Abund	Formula	Tgt Mass	Diff (ppm)	MFG Formula	DB Formula
Cpd 1: C14 H22 O3 Si	0.179	[illegible]	[illegible]	C14 H22 O3 Si	[illegible]	[illegible]	C14 H22 O3 Si	C14 H22 O3 Si

Compound Label	m/z	RT	Algorithm	Mass
Cpd 1: C14 H22 O3 Si	289.1235	0.179	Find By Formula	286.1344

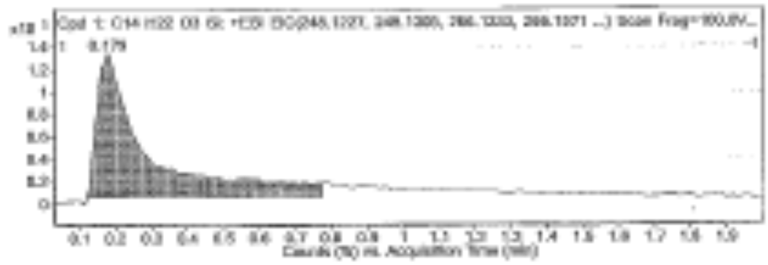

MS Spectrum

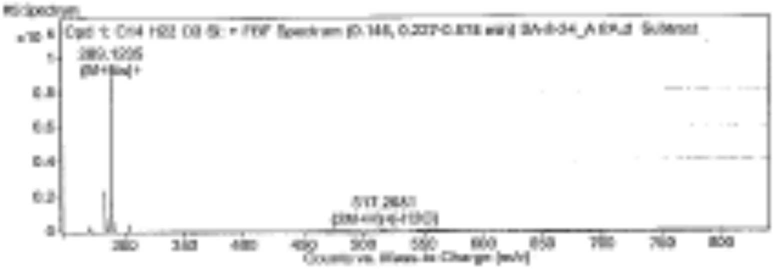

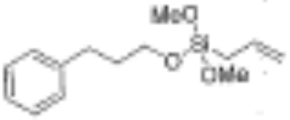

MS Zoomed Spectrum

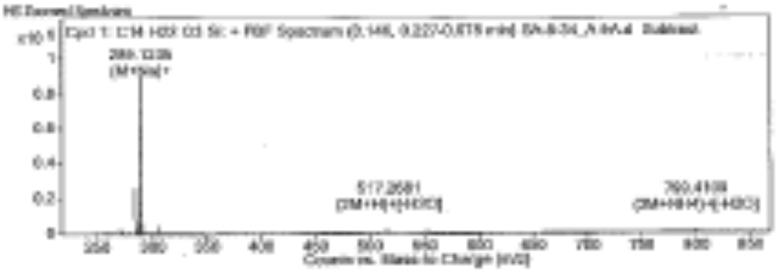

MS Spectrum Peak List

m/z	z	Abund	Formula	Ion
[illegible]	1	[illegible]	[illegible]	[illegible]
[illegible]	1	[illegible]	[illegible]	[M+NH4]+
[illegible]	1	[illegible]	[illegible]	[M+Na]+
[illegible]	1	[illegible]	[illegible]	[M+K]+
[illegible]	1	[illegible]	[illegible]	[illegible]
[illegible]	1	[illegible]	[illegible]	[2M+NH4]+
[illegible]	1	[illegible]	[illegible]	[illegible]
[illegible]	1	[illegible]	[illegible]	[2M+Na]+
[illegible]	1	[illegible]	[illegible]	[illegible]
[illegible]	1	[illegible]	[illegible]	[2M+K]+

--- End Of Report ---

Figure S76. *High resolution mass of allyldimethoxy(3-phenylpropoxy)silane*

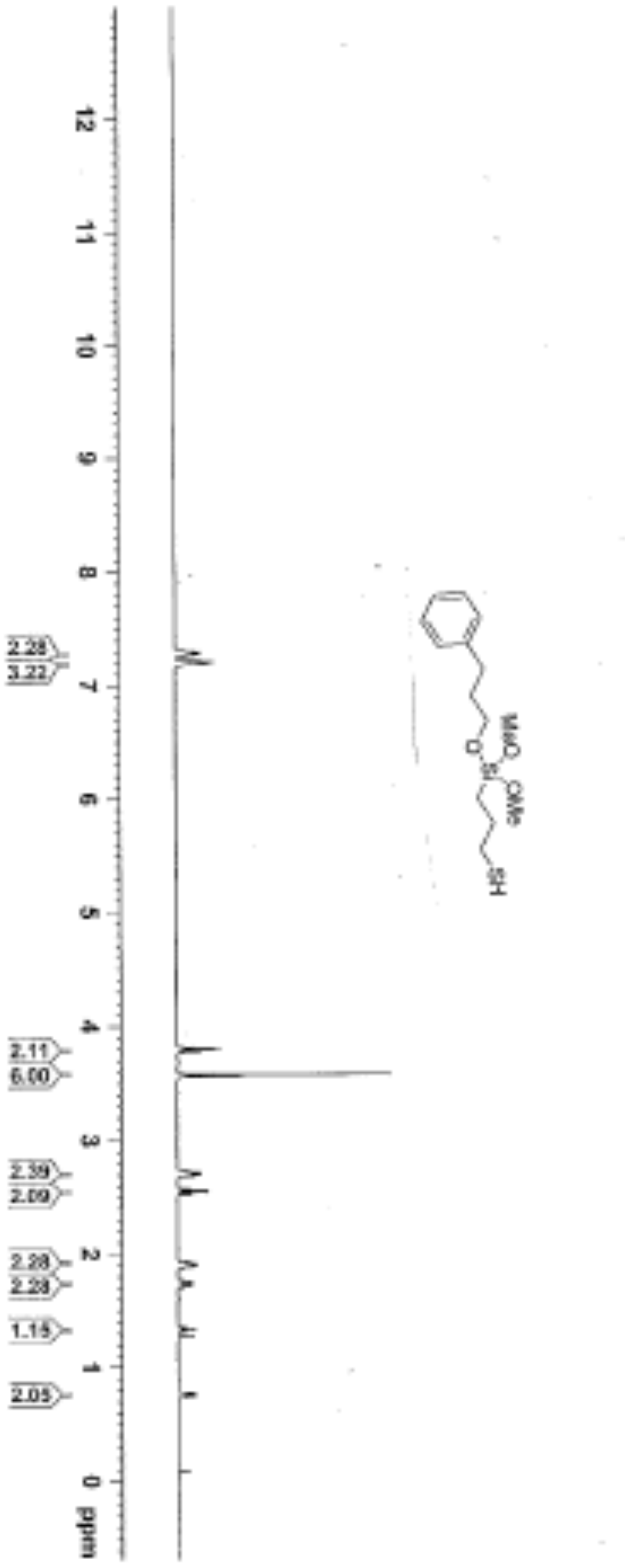

Figure S77. *^{1}H NMR spectrum of 3-(dimethoxy(3-phenylpropoxy)-propane-1-thiol*

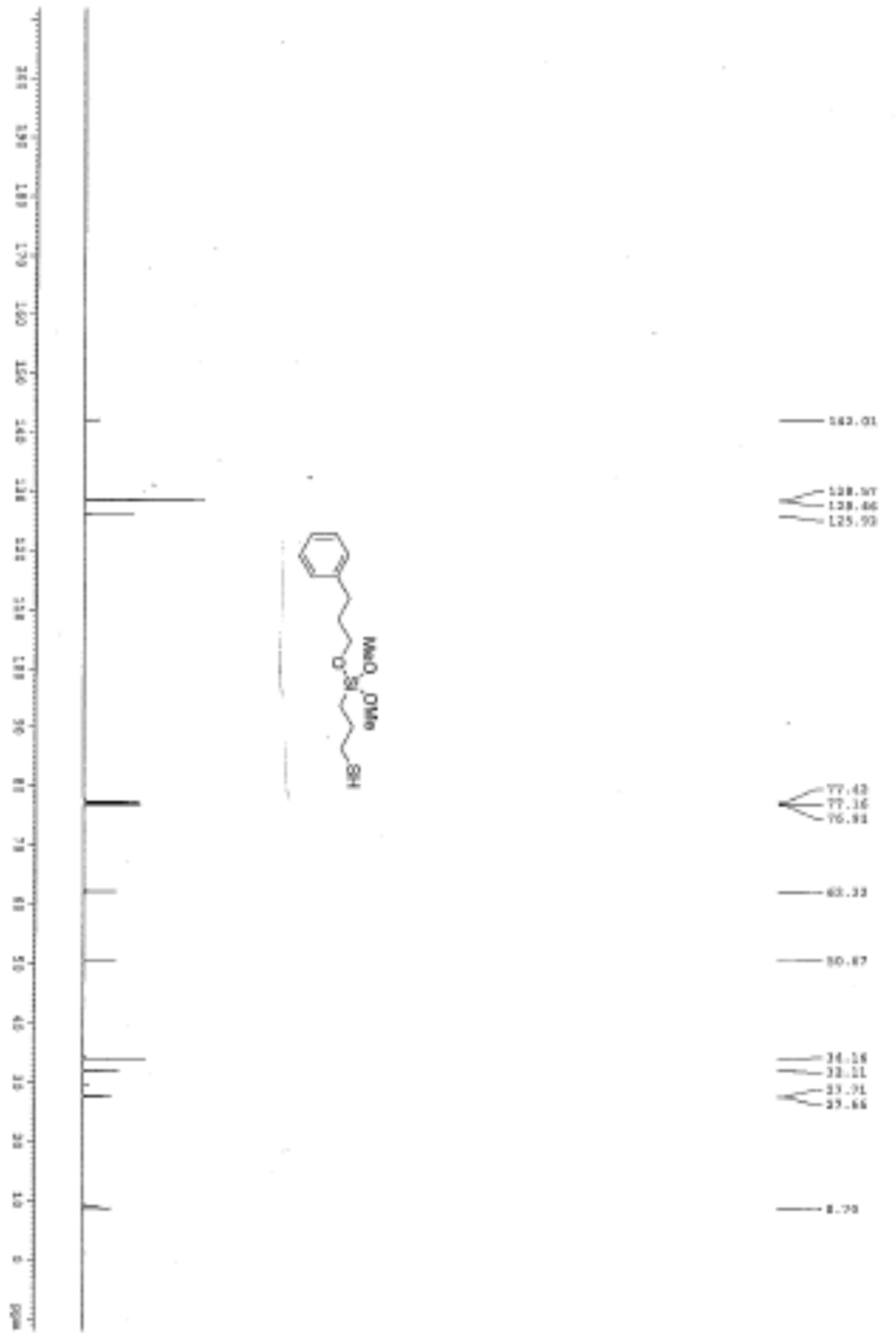

Figure S78. *^{13}C NMR spectrum of 3-(dimethoxy(3-phenylpropoxy)-propane-1-thiol*

Qualitative Compound Report

Data File	[illegible]	Sample Name	[illegible]
Sample Type	Sample	Position	[illegible]
Instrument Name	QTOF	User Name	[illegible]
Acq Method	[illegible]	Acquired Time	[illegible]
DNA Calibration Status	[redacted]	DA Method	[illegible]
Comment			

Acquisition SW Version	[illegible] series TOF/6200 series Q-TOF [illegible]

Compound Table

Compound Label	RT	Mass	Abund	Formula	Tgt Mass	Diff (ppm)	MFG Formula	DB Formula
Cpd 1: C14 H24 O3 S Si	[illegible]	[illegible]	[illegible]	C14 H24 O3 S Si	[illegible]	[illegible]	C14 H24 O3 S Si	C14 H24 O3 S Si

Compound Label	m/z	RT	Algorithm	Mass
Cpd 1: C14 H24 O3 S Si	323.1118	3.582	Find By Formula	300.1225

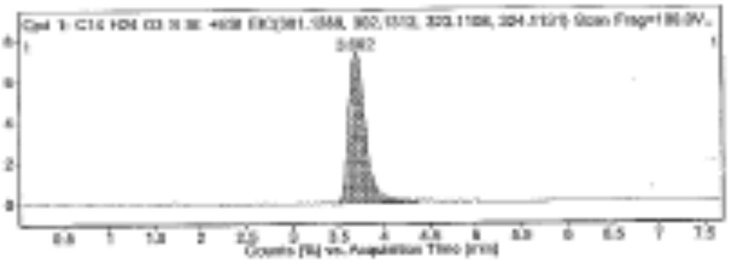

MS Spectrum

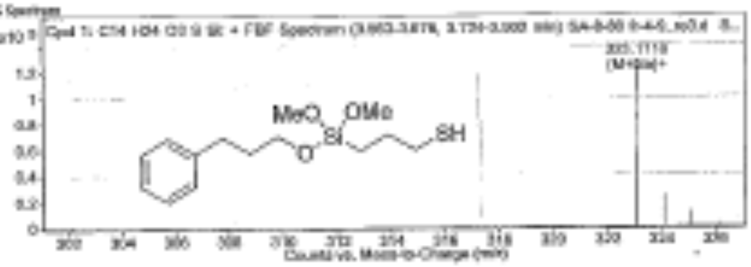

MS Zoomed Spectrum

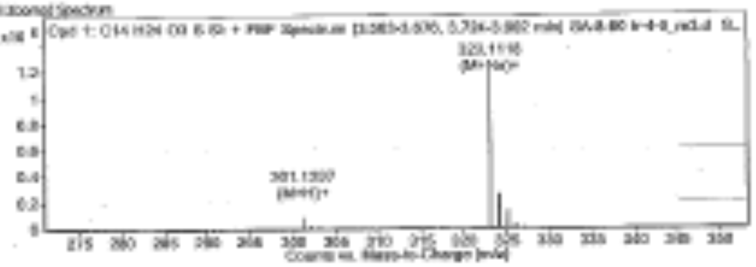

MS Spectrum Peak List

m/z	z	Abund	Formula	Ion
301.1297	1	[illegible]	[illegible]	(M+H)+
[illegible]	1	[illegible]	[illegible]	(M+H)+
[illegible]	1	[illegible]	[illegible]	(M+H)+
323.1118	1	[illegible]	[illegible]	(M+Na)+
[illegible]	1	[illegible]	[illegible]	(M+Na)+
[illegible]	1	[illegible]	[illegible]	(M+Na)+
[illegible]	1	[illegible]	[illegible]	(M+Na)+
[illegible]	1	[illegible]	[illegible]	(M+Na)+

--- End Of Report ---

Figure S79. *High resolution mass of 3-(dimethoxy(3-phenylpropoxy)-propane-1-thiol*

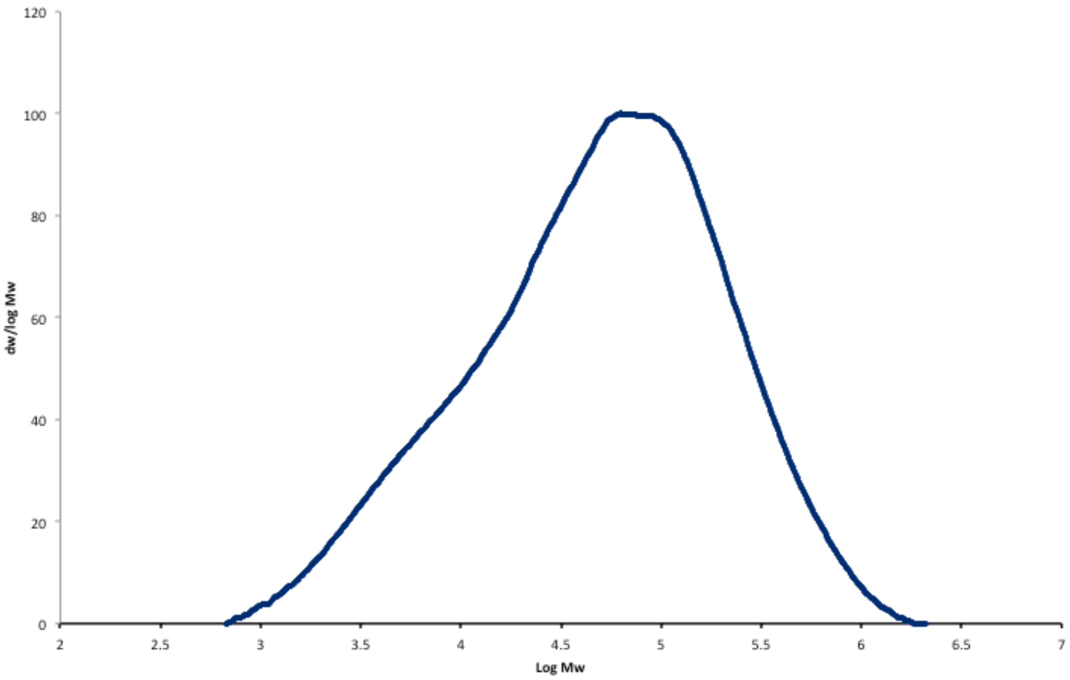

Figure S80. MWD curve (by SEC in 05% LiCl/DMAC) of the formic acid route fabricated NFC.

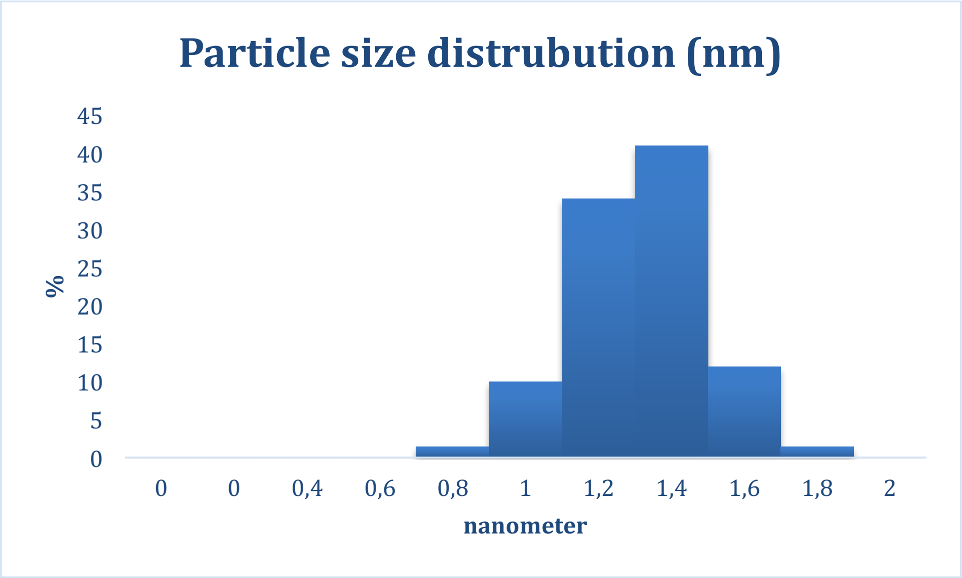

Figure S81. Pd Size distribution.
